# Pathogen Screening for Possible Causes of Meningitis/Encephalitis in Wild Carnivores From Saxony-Anhalt

**DOI:** 10.3389/fvets.2022.826355

**Published:** 2022-04-07

**Authors:** Jennifer Höche, Robert Valerio House, Anja Heinrich, Annette Schliephake, Kerstin Albrecht, Martin Pfeffer, Christin Ellenberger

**Affiliations:** ^1^Department of Veterinary Medicine, State Office for Consumer Protection Saxony-Anhalt, Stendal, Germany; ^2^Centre of Veterinary Public Health, Institute of Animal Hygiene and Veterinary Public Health, University of Leipzig, Leipzig, Germany

**Keywords:** canine distemper virus (CDV), canine parvovirus (CPV-2), fox circovirus, red fox (*Vulpes vulpes*), meningoencephalitis, zoonosis, wildlife, viral infection

## Abstract

Inflammation in meninges and/or brain is regularly noticed in red foxes and other wild carnivores during rabies control programs. Despite negative rabies virus (RABV) results, the etiologies of these cases remain unknown. Thus, the aim of this study was to provide an overview of the occurrence of pathogens that may cause diseases in the brains of wild carnivores and pose a risk to humans and other animals. In addition to RABV and canine distemper virus (CDV), a variety of pathogens, including members of *Flaviviridae, Bornaviridae, Herpesviridae, Circoviridae*, as well as bacteria and parasites can also cause brain lesions. In 2016 and 2017, brain samples of 1,124 wild carnivores were examined by direct fluorescent antibody test for RABV as well as (reverse-transcriptase) quantitative polymerase chain reaction (PCR) for the presence of CDV as part of a monitoring program in Saxony-Anhalt, Germany. Here, we applied similar methods to specifically detect suid herpesvirus 1 (SuHV-1), West Nile virus (WNV), Borna disease virus 1 (BoDV-1), canid alphaherpesvirus 1 (CaHV-1), canine parvovirus type 2 (CPV-2), fox circovirus (FoxCV), and *Neospora caninum* (*N. caninum*). Further, bacteriogical examination for the existence of *Listeria monocytogenes* (*L. monocytogenes*) and immunohistochemistry of selected cases to detect *Toxoplasma gondii* (*T. gondii*) antigen were performed. Of all pathogens studied, CDV was found most frequently (31.05%), followed by FoxCV (6.80%), CPV-2 (6.41%), *T. gondii* (4/15; 26.67%), nematode larvae (1.51%), *L. monocytogenes* (0.3%), and various other bacterial pathogens (1.42%). In 68 of these cases (6.05%), multiple pathogen combinations were present simultaneously. However, RABV, WNV, BoDV-1, SuHV-1, CaHV-1, and *N. caninum* were not detected. The majority of the histopathological changes in 440 animals were inflammation (320/440; 72.73%), predominantly non-suppurative in character (280/320; 87.50%), and in many cases in combination with gliosis, satellitosis, neuronophagia, neuronal necrosis, and/or vacuolization/demyelination, or in single cases with malacia. Thus, it could be shown that wild carnivores in Saxony-Anhalt are carriers mainly for CDV and sometimes also for other, partly zoonotic pathogens. Therefore, the existing monitoring program should be expanded to assess the spill-over risk from wild carnivores to humans and other animals and to demonstrate the role of wild carnivores in the epidemiology of these zoonotic pathogens.

## 1. Introduction

Inflammatory processes in the brain are mainly triggered by a variety of infectious agents. Known viruses in carnivores, in addition to rabies virus (RABV) (family *Rhabdoviridae*) (1) and canine distemper virus (CDV) (family *Paramyxoviridae*) (2, 3), include members of the families of *Flaviviridae* (4), *Herpesviridae* (5, 6), *Bornaviridae* (7, 8), *Parvoviridae* (9), and *Circoviridae* (10). Non-viral infectious agents that can also cause inflammations in the brain are for instance *Listeria monocytogenes (L. monocytogenes)* (11–13), *T. gondii* (14), and *N. caninum* (15). This portfolio is not exhaustive and depends on the geographical region and the carnivore fauna present. For Germany, as for many other countries, nowadays, this is not a fixed status quo, rather a dynamic process with invasive species intruding, e.g., raccoons (*Procyon lotor*) from North America or raccoon dogs (*Nyctereutes procyonoides*) more recently from Asia. A change in the carnivore fauna necessarily goes along with a change of endo- and ecto-parasites and the spectrum of bacterial and viral diseases associated with the “new” species becoming endemic (16, 17). However, besides monitoring programs targeting the status “free of terrestrial rabies” according to the German legislation (18), wild carnivores are not monitored for any etiological pathogen causing encephalitis or meningitis, whether zoonotic or not.

Our first objective was to judge the potential impact of invasive species, as raccoons, in particular, outnumber the ubiquitous red fox (*Vulpes vulpes*) in certain regions of Germany, including Saxony-Anhalt. In the period 2015–2017 alone, about 19,500 red foxes and 26,100 raccoons were shot per year in Saxony-Anhalt (19). Thus, these two carnivore species also represent the majority of animals sent in for rabies-testing. Other submitted animals are usually a few individual animals, such as raccoon dogs, martens of different species, and badgers (*Meles meles*). Many of the animals listed above live in close proximity to humans, making them a potential risk factor for transmission of zoonotic or emerging diseases (20–22).

The second motivation for our study stems from the histopathological findings acquired during the routine rabies testing every year (18). In 2015, histopathological examinations of brain samples revealed inflammatory processes in the meninges and/or brain in about 10% of the animals. While RABV was never detected, in two-third of the cases in 2015 these inflammatory processes could not be etiologically explained. In the case of meningitis and/or encephalitis, the monitoring program in Saxony-Anhalt only involves virus isolation in the cell culture for RABV and molecular biological examination for CDV (23). Other possible etiologies are not further considered. Therefore, we wanted to investigate which etiologies could cause the inflammatory processes in the central nervous system in all animals with meningitis and/or encephalitis, especially if RABV and CDV could not be detected. In Saxony-Anhalt little is known about the occurrence and outcome of any encephalitis-causing pathogens in the population of wild carnivores. In addition, we wanted to identify especially pathogens that have a zoonotic potential and therefore can be a threat to humans, domestic and zoo animals, or the wildlife population. If specific diseases occurred, an attempt was made to identify local epidemiological reservoirs of pathogens based on the distribution of diseases in the wildlife population studied. Potential transmission pathways and/or sources of infection should be considered and discussed on the basis of the results. This would help to protect persons (e.g., veterinarians, farmers, and hunters) who have regular contact with wild carnivores and, where appropriate, domestic and zoo animals. In case of occurrence of zoonotically important pathogens, a permanent integration of these pathogens into monitoring programs could be intended. Special emphasis was set on viral infections.

## 2. Materials and Methods

### 2.1. Study Area

We conducted a cross-sectional study on the occurrence of meningitis and/or encephalitis in wild carnivores in Saxony-Anhalt, a federal state in the eastern part of Germany with an area of 20.451,74 km^2^. The state consists of 14 administrative districts including 218 municipalities with about 2.2 million inhabitants (24).

### 2.2. Animals

**Figure 1:**
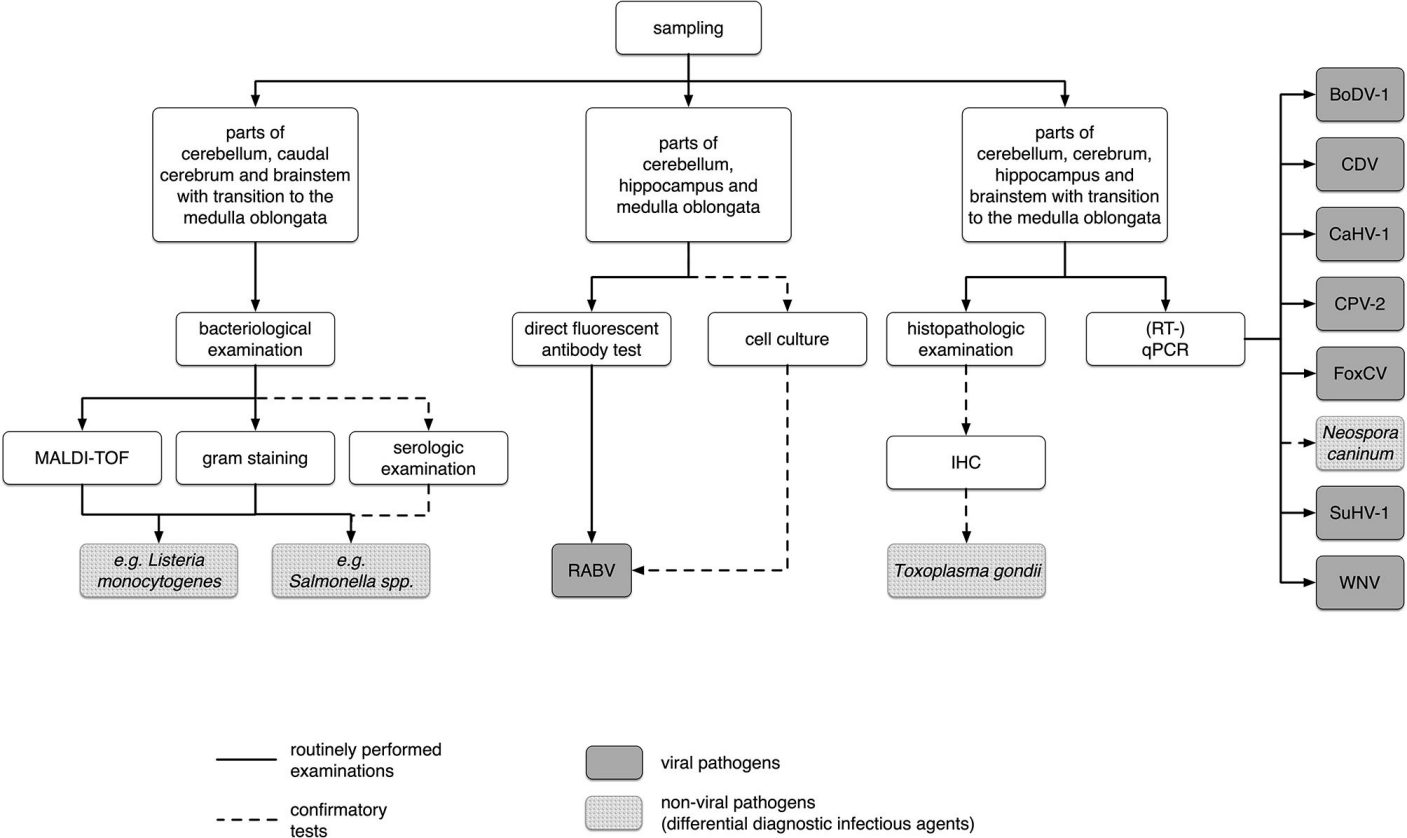
Scheme of all samples used and examinations performed in this study. BoDV-1, Borna disease virus 1; CDV, canine distemper virus; CaHV-1, canid alphaherpesvirus 1; CPV-2, canine parvovirus type 2, 2a, 2b, or 2c; FoxCV, fox circovirus; IHC, immunohistochemistry; MALDI-TOFF, matrix-assisted laser desorption/ionization-time of flight mass spectrometry; (RT-)qPCR, (reverse transcription) quantitative polymerase chain reaction; RABV, rabies virus; SuHV-1, suid herpesvirus 1; WNV, West Nile virus.

In 2016 and 2017, 1,124wild carnivores [860red foxes, 204raccoons, 34raccoon dogs, 12badgers, and 14martens (11stone martens (*Martes foina*) and three martens without further characterization)] were sent to the Department of Veterinary Medicine in the State Office for Consumer Protection Saxony-Anhalt for RABV testing. Animals were either found dead or shot by hunters or rangers. No animals were killed just for the purpose of this study. The animals were usually sent in at a time-span not exceeding one week after detection or hunting and were dissected immediately. Species, gender, approximate dental age [juvenile <0.5 years and adult >0.5 years, according to Habermehl (25)], date of hunting or finding, locality, and, if indicated by the hunter, behavior of each animal were recorded. Abnormal behavior was defined if the senders reported lack of shyness, lack of escape reflex, staggering gaits, or if animals have bitten people or pets. Data can be found in Supplementary Table S1 and a scheme of all examinations performed is given in Figure 1.

### 2.3. Direct Fluorescent Antibody Test for Detection of Rabies Virus

For detection of RABV, impression smears of the cerebellum, hippocampus, and medulla oblongata were examined in a direct fluorescent antibody test as stated in the German official methods collection (26). A monoclonal anti-rabies FITC-labeled conjugate (sifin diagnostics GmbH, Berlin, Germany) in a working dilution of 1:20 was used. As a positive control, we applied baby hamster kidney (BHK) 21 C13 cells (Collection of Cell Lines in Veterinary Medicine 194, Friedrich-Loeffler-Institute, Greifswald-Insel Riems, Germany) infected with laboratory RABV strain CVS-11 (Riems Virus Collection, VR 959, Friedrich-Loeffler-Institute, Greifswald-Insel Riems, Germany). Non-infected BHK 21 C13 cells served as a negative control.

### 2.4. Bacteriological Examination

Samples of the cerebellum, caudal cerebrum, and brain stem with a transition to medulla oblongata were freshly taken and cultured on blood agar with the addition of 5% sheep blood (Thermo Fisher Diagnostics GmbH Microbiology, Wesel, Germany). As selective culture media, we used Gassner and Brilliance Listeria Agar (Thermo Fisher Diagnostics GmbH Microbiology, Wesel, Germany). These agars were incubated for 48 h at 37 °C under aerobic conditions. Bacterial growth was controlled after 24 and 48 h. Additionally, microaerophilic incubation took place on blood agar with the addition of 5% sheep blood for 96 h at 37 °C and 12% CO2. Here, bacterial growth was controlled after 96 h.

Plates were considered negative if no potentially pathogenic bacterial colonies were present. Otherwise, potentially pathogenic bacteria were subcultured on agar plates for further examinations.

To determine the bacterial species, gram-staining according to standard procedures followed (27). Additionally, matrix-assisted laser desorption/ionization-time of flight mass spectrometry (MALDI-TOF) analysis was conducted using IVD MALDI Biotyper 2.3 (Bruker Daltonik GmbH, Bremen, Germany) with direct transfer method following instructions in manufacturer's user manual.

In case of occurrence of suspicious colonies of *Salmonella* spp., we performed a direct slide agglutination test according to standard procedures for further characterization (28). Here, the results were confirmed and strains were further subtyped by the National Reference Laboratory for the Analysis and Testing of Zoonoses (*Salmonella*) in the Federal Institute for Risk Assessment (BfR) Berlin.

### 2.5. Histopathological Examination

For histopathological examination, samples from the cerebellum, cerebrum, hippocampus, and brain stem with the transition to the medulla oblongata were fixed in 10% formalin. Next, the fixed samples were embedded in paraffin (Medite GmbH, Burgdorf, Germany) according to standard procedures using an automatic tissue embedder (Medite GmbH, Burgdorf, Germany). Subsequently, 2–4 μm thick paraffin sections were stained with haemalaun and eosin (HE) following a standard protocol (29).

Light microscopic examinations were performed using an Olympus CX21FS2 microscope (OLYMPUS EUROPA SE & CO. KG, Hamburg, Germany), and the histopathological findings were documented for each animal according to a uniform evaluation scheme developed for this study (Supplementary Figure S1). Exceptions were made with animals with acute craniocerebral trauma in which parts of the brain were unusable for histopathological examination due to severe tissue destruction (*n* = 98). In these cases, only the histopathologically evaluable brain areas were included in the light microscopic examination.

In principle, a distinction was made between reactive and degenerative changes. In addition, the occurrence of viral inclusion bodies (intranuclear (cowdry A or B type) and/or intracytoplasmic localizations) was recorded.

The reactive changes included inflammation in the leptomeninx and in the brain, gliosis, satellitosis, neuronophagia, and neuronal necrosis which we defined according to Baumgärtner (30), Zachary (31), Baumgärtner and Schmidt (32), and Wohlsein et al. (33). Vacuolization of the neuropil, demyelination, and malacia were summarized as degenerative changes (30–35).

Some animals showed postmortem artifacts (autolysis, putrefaction) and/or were in a deep-frozen state (*n* = 240). Due to these artifacts, degenerative changes were not assessed (36–38). Since the *vacuolization* (occurrence of optically empty (hollow) spaces) in the HE-stained section could be the result of edema, demyelination, or both processes, the terms were used together descriptively.

Furthermore, by using HE-stain only, a differentiation of the single cells (macro-, microglia, macrophages) involved in the processes mentioned above was not possible. Therefore, the terms activated microglial cells and macrophages are used synonymously.

Regarding inflammation, we distinguished suppurative, non-suppurative, granulomatous, and eosinophilic forms (30). Coexisting inflammatory forms were defined as mixed. Additionally, the distribution pattern was also considered. The inflammation was graded according to the criteria listed in Supplementary Table S2. The indicated cell numbers refer to a High Power Field (HPF = one visual field at X400 magnification).

### 2.6. Immunohistochemistry for Detection of *Toxoplasma gondii* Antigen

In selected cases (*n* = 15), immunohistochemistry (IHC) for detection of *T. gondii* antigen was performed. Thus, animals with typical inflammatory patterns or the occurrence of suspicious parasitic structures in the brain in the HE-stained sections were further examined, and the peroxidase antiperoxidase (PAP) method was used. For this purpose, the sections made by the paraffin-blocked tissues were mounted on Superfrost Ultra Plus slides (Thermo Fisher Scientific Inc., Waltham, USA). Subsequently, the sections were dewaxed in grading alcohol series followed by pretreatment with heat-induced epitope retrieval-Tris EDTA buffer (Zytomed Systems GmbH, Berlin, Germany). As primary antibody, we used rabbit-anti *T. gondii* antibody (Zytomed Systems GmbH, Berlin, Germany) in a dilution of 1:100. The incubation time for this step was 1 h at room temperature followed by incubation with a PAP complex according to recommendations in ZytoChem-Plus HRP Polymer-Kit description (Zytomed Systems GmbH, Berlin, Germany) for 30 min at room temperature. After signal detection with a freshly prepared solution of 300 μl of 3,3′-diaminobenzidinetetrahydrochloride (DAB Substrate Kit, Zytomed Systems GmbH, Berlin, Germany) for 15 min at room temperature, the sections were counterstained with Mayers' Haemalaun solution (Carl Roth GmbH + Co. KG, Karlsruhe, Germany), finally dehydrated and mounted with Thermo Scientific™ Richard-Allan Scientific™ Cytoseal^TM^ XYL mounting media (Thermo Fisher Scientific Inc., Kalamazoo, MI, USA). Sections were rinsed thoroughly with Tris-buffered saline between each step.

As a positive control, liver and spleen sections from a cat naturally infected with *T. gondii* were used. Positive reaction products were obtained as strong brown and fine-granular (parasitic stages) or homogeneously membranous (wall of parasitic cysts) structures. These reactions were not detected in the negative controls. Two distinct negative controls were included in the study. The first one was the positive control section from the infected cat and the second one was the test material. Both were treated like the positive control but without using the primary antibody. None of the negative controls showed any brown reaction products.

### 2.7. Molecular Methods

#### 2.7.1. Preparation of Sample Material

Approximately pea-sized pieces of the cerebellum, cerebrum, hippocampus, and brain stem with a transition to medulla oblongata were pooled and homogenized in phosphate-buffered salt solution (Biochrom GmbH, Berlin, Germany) with the addition of 1% Gentamycin Sulfate (Merck Chemicals GmbH, Darmstadt, Germany) in PrioGENIZER™ Homogenization Device (Thermo Fisher Scientific Inc., Waltham, USA) for molecular detection of suid herpesvirus 1 (SuHV-1), West nile virus (WNV), Borna disease virus 1 (BoDV-1), CDV, canid alphaherpesvirus 1 (CaHV-1), canine parvovirus type 2, 2a, 2b, or 2c (CPV-2), without differentiating the antigenic variants, fox circovirus (FoxCV), a member of species *Canine circovirus*, and *N. caninum*. Until examination, material was frozen at –80 °C.

#### 2.7.2. Nucleic Acid Extraction

For parallel RNA- and DNA-extraction, 100 μl of homogenate was purified using KingFisher™ Flex purification system (Thermo Fisher Scientific Inc., Vantaa, Finland) in combination with MagMAX CORE Nucleic Acid Purification Kit (Life Technologies Corporation, Austin, TX, USA) according to Digestion Workflow in the manufacturer's instructions. The correct nucleic acid extraction and the lack of inhibition were confirmed by detection of the beta-actin gene in a qPCR according to Wernike et al. (39).

#### 2.7.3. Nucleic Acid Amplification

For amplification of DNA from SuHV-1, CaHV-1, CPV-2, FoxCV, and *N. caninum*, QuantiTect^®^ Multiplex PCR NoROX Kit (Qiagen, Hilden, Germany), and for amplification of RNA from WNV, BoDV-1, and CDV QuantiFast^®^ Pathogen RT-PCR + IC Kit (Qiagen, Hilden, Germany) were used according to the manufacturer's instructions. Detailed (RT-)qPCR conditions for each pathogen are listed in Supplementary Table S3. All (RT-)qPCR reactions were run on AriaMx Real-time PCR Systems (Agilent Technologies, Santa Clara, USA). Samples were considered positive if the cycle threshold was less than or equal to 40. Results considered positive were repeated individually using a new sample from the individual animal for confirmation. In the following, these positive samples are referred to as *infection* as described in Pschyrembel (40) even if there was no histomorphological correlate in the examined brain sections. Positive controls included material from infected cell cultures (BoDV-1 and WNV, kindly provided by colleagues from the Friedrich-Loeffler-Institute, Germany; CaHV-1 and CPV-2, kindly provided by colleagues from the University of Leipzig, Germany) or organ samples from naturally infected animals (FoxCV, kindly provided by colleagues Department of Virology, Erasmus MC, Rotterdam, Netherlands Virology Department of the Netherlands as well as the Virology Department of the Istituto Zooprofilattico Sperimentale dell'Abruzzo e Molise (IZSAM), Teramo, Italy; CDV, SuHV-1, and *N. caninum*, own sample material whose validity was confirmed by other laboratories). Two individually confirmed negative brain samples and RNase-free water were used as no template control in each individual (RT-)qPCR run.

### 2.8. Statistical Analysis

The 95% confidence intervals (CIs) for determination of the apparent prevalence were calculated with the formula according to Clopper-Pearson using package Statsmodels in Python (41). Chi-squared test with Yates' correction for continuity was performed using package SciPy in Python (42) for testing independency of prevalence and age, gender or season. A *p*-value of < 0.05 was chosen as threshold for statistical significance. Maps for prevalence in administrative districts and independent cities were created using material from http://opendatalab.de/projects/geojson-utilities/ (accessed on 2022/01/12).

## 3. Results

### 3.1. Animal Data

In 2016 and 2017, 1,124animals were sent to our department. Of these, 1,044animals (92.88%) were adults and 80juveniles (7.12%), 644were male (57.30%) and 480were female (42.70%), 1006 (89.50%) were shot and 97 (8.63%) were found dead or were killed in an accident. For 21animals (1.87%), the senders did not provide details of the cause of death. There was information on behavior for 136animals (12.10%). Abnormal behavior was reported for 121 (88.97%) of these animals. The remaining 15individuals showed normal behavior (15/136; 11.03%). The interested reader will find a summary of behavioral data in context with the pathogens detected in Supplementary Section 1. Animals were sent in from every administrative district of Saxony-Anhalt. Due to hunting season, we received about two-third of the animals in the autumn and winter months [365/1,124in autumn (32.47%) and 373/1,124in winter (33.19%)]. For details of the individual animals, please refer to Supplementary Table S1.

### 3.2. Histopathological Results

Histopathological changes were observed independently of pathogen detection in 440of the 1,124animals (39.15%). Among these, 320 (72.73%) showed an inflammation with or without various combinations of gliosis, satellitosis, neuronophagia, neuronal necrosis, vacuolization/demyelination, and/or in rare cases with malacia.

**Figure 2:**
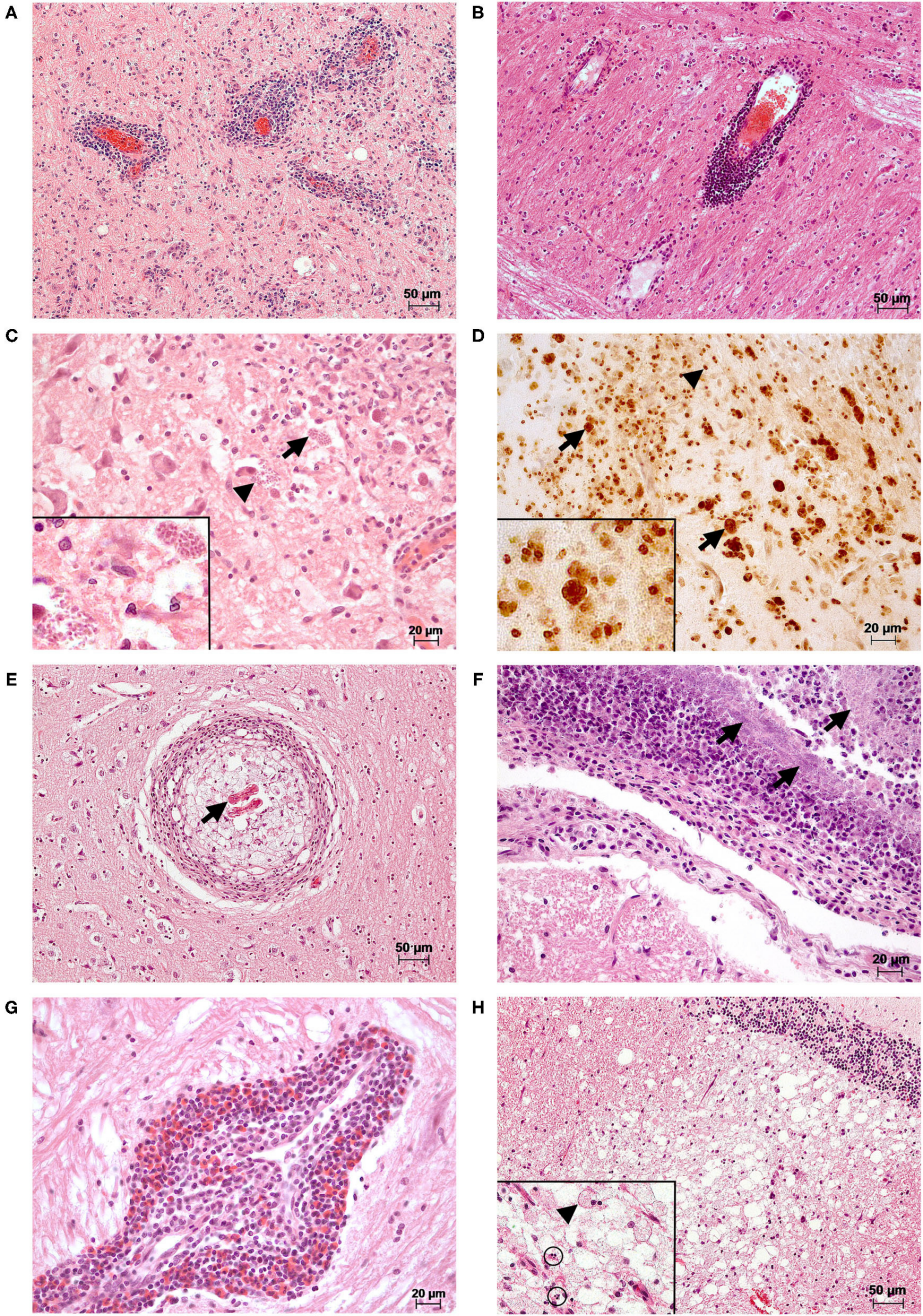
Histopathological changes observed in the brains of selected cases. **(A)** A marked multifocal, mainly perivascular lymphoplasmacellular meningoencephalitis was present in the brain stem of a female adult red fox with detection of CDV (RT-qPCR; Lab-ID: 17410254906). Changes were observed in all examined localizations of the brain (meninges not shown). HE **(B)** There is a marked multifocal perivascular lymphocytic encephalitis in the brain stem of a male adult red fox with CDV (RT-qPCR; Lab-ID: 16410421068). The animal also showed lymphopasmacellular meningitis and gliosis in the cerebrum and brain stem (data not shown). HE **(C)** A marked multifocal granulomatous encephalitis was seen in the brain stem of a male adult red fox with abnormal behavior with detection of CDV (RT-qPCR) and *T. gondii* antigen (immunohistochemistry (IHC); Lab-ID: 17410039063). In the brain tissue, there are intralesional protozoal cysts (arrows) and free parasitic development stages (arrowheads). Inset: higher magnification of parasites. HE **(D)** Immunohistochemical detection of *T. gondii* antigen of section **(C)**. Positive reaction products were obtained as strong brown and fine-granular (parasitic stages, arrowheads) or homogeneous membranous (wall of parasitic cysts, arrows) structures. Inset: higher magnification of positive parasitic structures. IHC **(E)** Nematode larvae (arrow) are located in the center of a marked focal granulomatous encephalitis found in a section of the cerebrum of a male adult red fox (Lab-ID: 17410444086). HE **(F)** In the brain stem of a male adult red fox infected with *Streptococcus canis* (Lab-ID: 17410316653), a marked suppurative meningitis with intralesional detection of myriads of coccoid bacteria (arrows) could be observed. Lesions were also found in the cerebrum and hippocampus, in both the brain tissue and meninges (data not shown). HE **(G)** A marked focal eosinophilic and perivascular dominated encephalitis was observed in the brain stem of a female adult raccoon with detection of CDV and CPV-2 [(RT)-qPCR; Lab-ID: 17410189710]. The eosinophilic inflammation is part of a mixed form together with plasma cells and lymphocytes. Additionally, other reactive changes such as gliosis, satellitosis, and neuronophagia were present in all examined localizations (data not shown). HE **(H)** A male adult red fox with detection of CDV (RT-qPCR; Lab-ID: 17410137421) showed marked vacuolization/demyelination and malacia in the white matter of the cerebellum. In all examined brain areas, there was additionally moderate multifocal lymphoplasmacellular encephalitis (data not shown). Inset: higher magnification of malacia with gitter cell (arrowhead) and single cell necrosis (circles) HE.

Inflammatory processes were mainly non-suppurative in character (280/320; 87.50%; Figures 2A,B). Furthermore, we found granulomatous (22/320; 6.88%, Figures 2C–E), suppurative (1/320; 0.31%; Figure 2F), eosinophilic (1/320; 0.31%) inflammation, and mixed (16/320; 5.00%; Figure 2G) forms.

In the remaining 120animals (27.27%), we observed gliosis, satellitosis, neuronophagia, neuronal necrosis, and/or vacuolization/demyelination (Figure 2H) either as single reactions or in a wide range of different combinations. These are described for the respective pathogen in the following chapters and findings of positive animals are listed in Tables 1–**3**. All histopathological findings for each animal are found in Supplementary Table S1.

**Table 1:** Histopathological findings in animals with canine distemper virus (CDV), canine parvovirus type 2 (CPV-2), or fox circovirus (FoxCV), irrespective of possible pathogen combinations.

	**CDV**		**CPV-2**		**FoxCV**	
	**Positive animals (*n*)**	**in % (CI)**	**Positive animals (*n*)**	**in % (CI)**	**Positive animals (*n*)**	**in % (CI)**
**Total number with histopathological changes**	205/349	58.74 (53.37–63.95)	23/72	31.94 (21.44–43.99)	42/77	54.55 (42.79–65.94)
**Inflammation**	161/205	78.54 (72.28–83.95)	18/23	78.26 (56.30–92.54)	30/42	71.43 (55.42–84.28)
Meningitis	63/161	39.13 (31.55–47.12)	9/18	50.00 (26.02–73.98)	13/30	43.33 (25.46–62.57)
Meningoencephalitis	60/161	37.27 (29.79–45.23)	4/18	22.22 (6.41–47.64)	11/30	36.67 (19.93–56.14)
Encephalitis	38/161	23.60 (17.28–30.93)	5/18	27.78 (9.69–53.48)	6/30	20.00 (7.71–38.57)
**Inflammation character**						
Non-suppurative	141/161	87.58 (81.47–92.24)	14/18	77.78 (52.36–93.59)	27/30	90.00 (73.47–97.89)
Granulomatous	13/161	8.07 (4.37–13.41)	3/18	16.67 (3.58–41.42)	1/30	3.33 (0.08–17.22)
Mixed	7/161	4.35 (1.77–8.75)	1/18	5.56 (0.14–27.29)	2/30	6.67 (0.82-22.07)
**Degree of inflammation**						
Minimal	49/161	30.43 (23.44–38.17)	3/18	16.67 (3.58–41.42)	11/30	36.67 (19.93–56.14)
Mild	88/161	54.66 (46.63–62.51)	13/18	72.22 (46.52–90.31)	14/30	46.67 (28.34–65.67)
Moderate	11/161	6.83 (3.46–11.90)				
Marked	13/161	8.07 (4.37–13.41)	2/18	11.11 (1.38–34.71)	5/30	16.67 (5.64–34.72)
**Localization of inflammation**						
Focal	18/161	11.18 (6.76–17.09)	5/18	27.78 (9.69–53.48)	3/30	10.00 (2.11–26.53)
Multifocal	143/161	88.82 (82.91–93.24)	13/18	72.22 (46.52–90.31)	27/30	90.00 (73.47–97.89)
Affected brain area^*^						
Cerebrum	137/161	85.09 (78.64–90.21)	14/18	77.78 (52.36–93.59)	24/30	80.00 (61.43–92.29)
Cerebellum	73/161	45.34 (37.49–53.37)	7/18	38.89 (17.30–64.25)	13/30	43.33 (25.46–62.57)
Hippocampus	25/161	15.53 (10.31–22.06)	3/18	16.67 (3.58–41.42)	5/30	16.67 (5.64–34.72)
Brain stem	65/161	40.37 (32.72–48.38)	6/18	33.33 (13.34–59.01)	12/30	40.00 (22.66–59.40)
Other reactive changes ^**^	114/205	55.61 (48.53–62.53)	12/23	52.17 (30.59–73.18)	19/42	45.24 (29.85–61.33)
Gliosis	113/114	99.12 (95.21–99.98)	12/12	100.00 (73.54–100.00)	19/19	100.00 (82.35–100.00)
Satellitosis	39/114	34.21 (25.58–43.68)	3/12	25.00 (5.49–57.19)	5/19	26.32 (9.15–51.20)
Neuronophagia	17/114	14.91 (8.93–22.80)	1/12	8.33 (0.21–38.48)	4/19	21.05 (6.05–45.57)
Neuronal necrosis	10/114	8.77 (4.29–15.54)			3/19	15.79 (3.38–39.58)
Other reactive changes with inflammation ^**^						
Gliosis	88/113	77.88 (69.10–85.14)	8/12	66.67 (34.89–90.08)	11/19	57.89 (33.50–79.75)
Satellitosis	28/39	71.79 (55.13–85.00)	2/3	66.67 (9.43–99.16)	2/5	40.00 (5.27–85.34)
Neuronophagia	11/17	64.71 (38.33–85.79)	1/1	100.00 (2.50–100.00)	1/4	25.00 (0.63–80.59)
Neuronal necrosis	6/10	60.00 (26.24–87.84)			1/3	33.33 (0.84–90.57)
Degenerative changes ^**^	89/205	43.41 (36.53–50.50)	7/23	30.43 (13.21–52.92)	14/42	33.33 (19.57–49.55)
Vacuolization/demyelination	88/89	98.88 (93.90–99.97)	7/7	100.00 (59.04–100.00)	14/14	100.00 (76.84–100.00)
Malacia	3/89	3.37 (0.70–9.54)				
Degenerative changes with inflammation ^**^						
Vacuolization/demyelination	59/88	67.05 (56.21–76.70)	5/7	71.43 (29.04–96.33)	7/14	50.00 (23.04–76.96)
Malacia	3/3	100.00 (29.24–100.00)				
**Combination of other reactive and degenerative changes**	44/205	21.46 (16.05–27.72)	3/23	13.04 (2.78–33.59)	7/42	16.67 (6.97–31.36)
With inflammation	34/44	77.27 (62.16–88.53)	2/3	66.67 (9.43–99.16)	4/7	57.14 (18.41–90.10)
**Total number without histopathological changes**	144/349	41.26 (36.05–46.63)	49/72	68.06 (56.01–78.56)	35/77	45.45 (34.06–57.21)

Number of affected animals (n), 95% confidence interval (CI), combinations of different affected brain areas possible (^*^), combinations of different findings possible (^**^).

### 3.3. Pathogens and Corresponding Findings

We detected CDV, CPV-2, and FoxCV alone, in various combinations together or with other non-viral infectious agents. In none of the animals we could find RABV, SuHV-1, WNV, BoDV-1, CaHV-1 or *N. caninum*. Bacterial species were present in the brains of 20animals, and *T. gondii* antigen (Figure 2D) was found in fouranimals. In the following, the results for the individual pathogens are described in more detail.

In general, the majority of animals with viral infections showed non-suppurative inflammation. However, occasionally granulomatous inflammation or mixed forms were noted. In approximately half of the cases with viral detection, inflammatory changes were associated with gliosis alone or in various combinations with satellitosis, neuronophagia, neuronal necrosis, and/or vacuolization/demyelination. Malacia occurred solely in threecases when CDV was detected (Figure 2H). In individual animals, different combinations of gliosis, satellitosis, neuronophagia, neuronal necrosis, and/or vacuolization/demyelination without inflammation were observed. A detailed listing of histopathological findings in virus-positive animals is provided in Table 1 and Supplementary Figures S2A–C.

#### 3.3.1. Canine Distemper Virus

**Figure 3:**
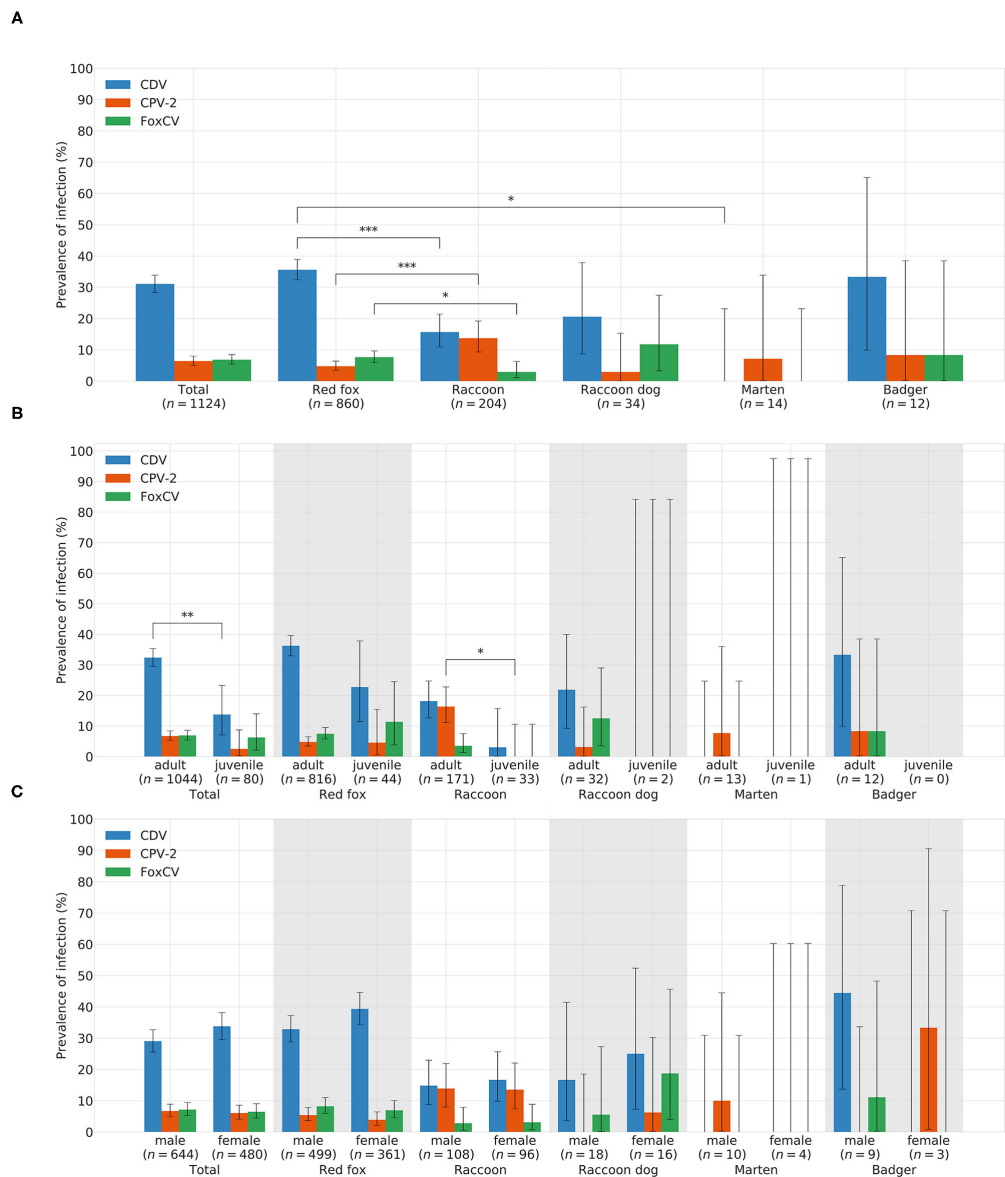
Prevelances of CDV, CPV-2, and FoxCV by **(A)** species, **(B)** age, and **(C)** gender. Error bars indicating 95% confidence intervals (CIs). Significant *p*-values are indicated as follows: *p ≤ 0.05; **p ≤ 0.01; ***p ≤ 0.001. Number of animals sent in (*n*).

**Figure 4:**
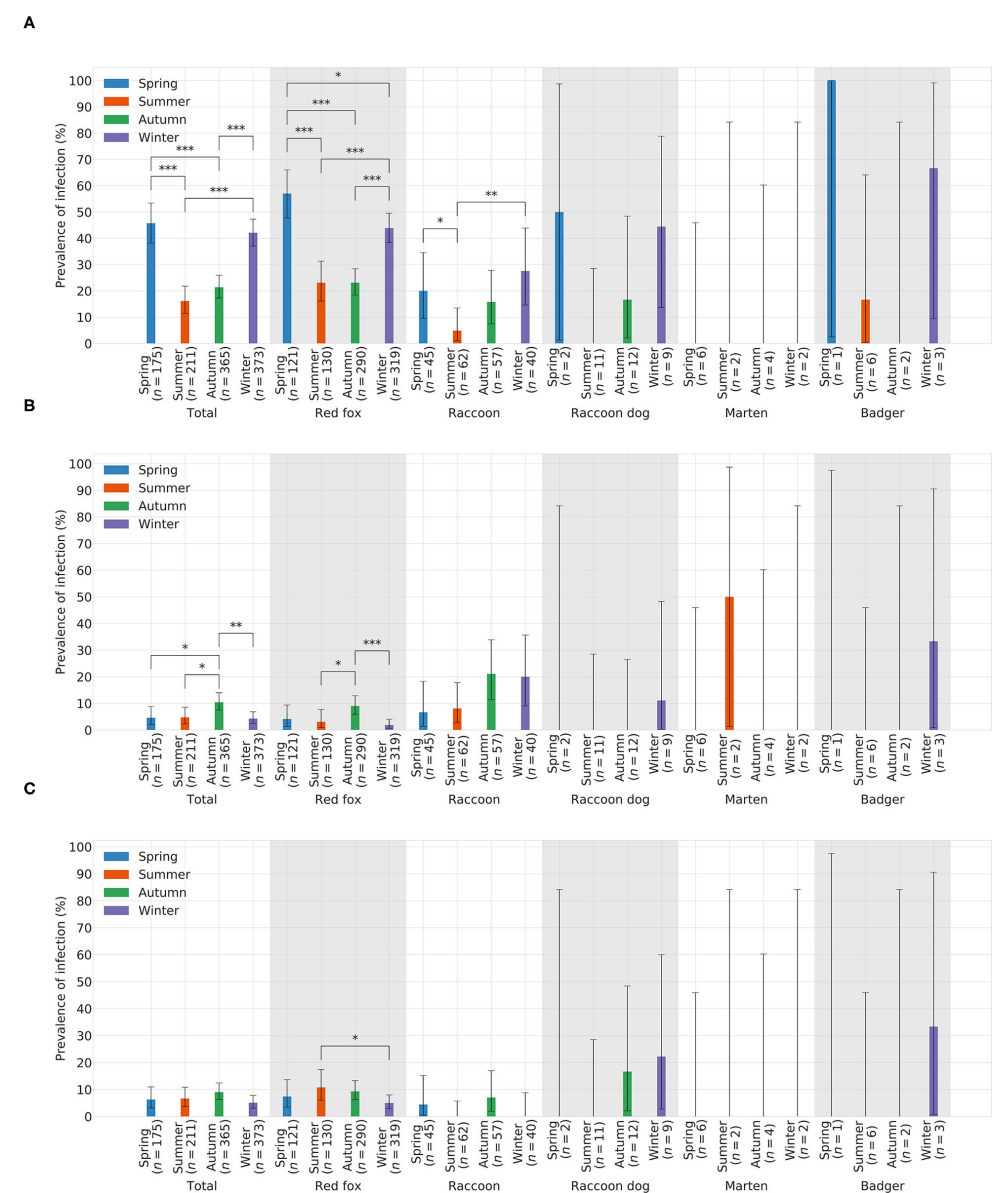
Prevelances of **(A)** CDV, **(B)** CPV-2, and **(C)** FoxCV by season. Error bars indicating 95% confidence intervals (CIs). Significant *p*-values are indicated as follows: *p ≤ 0.05; **p ≤ 0.01; ***p ≤ 0.001. Number of animals sent in (*n*).

**Figure 5:**
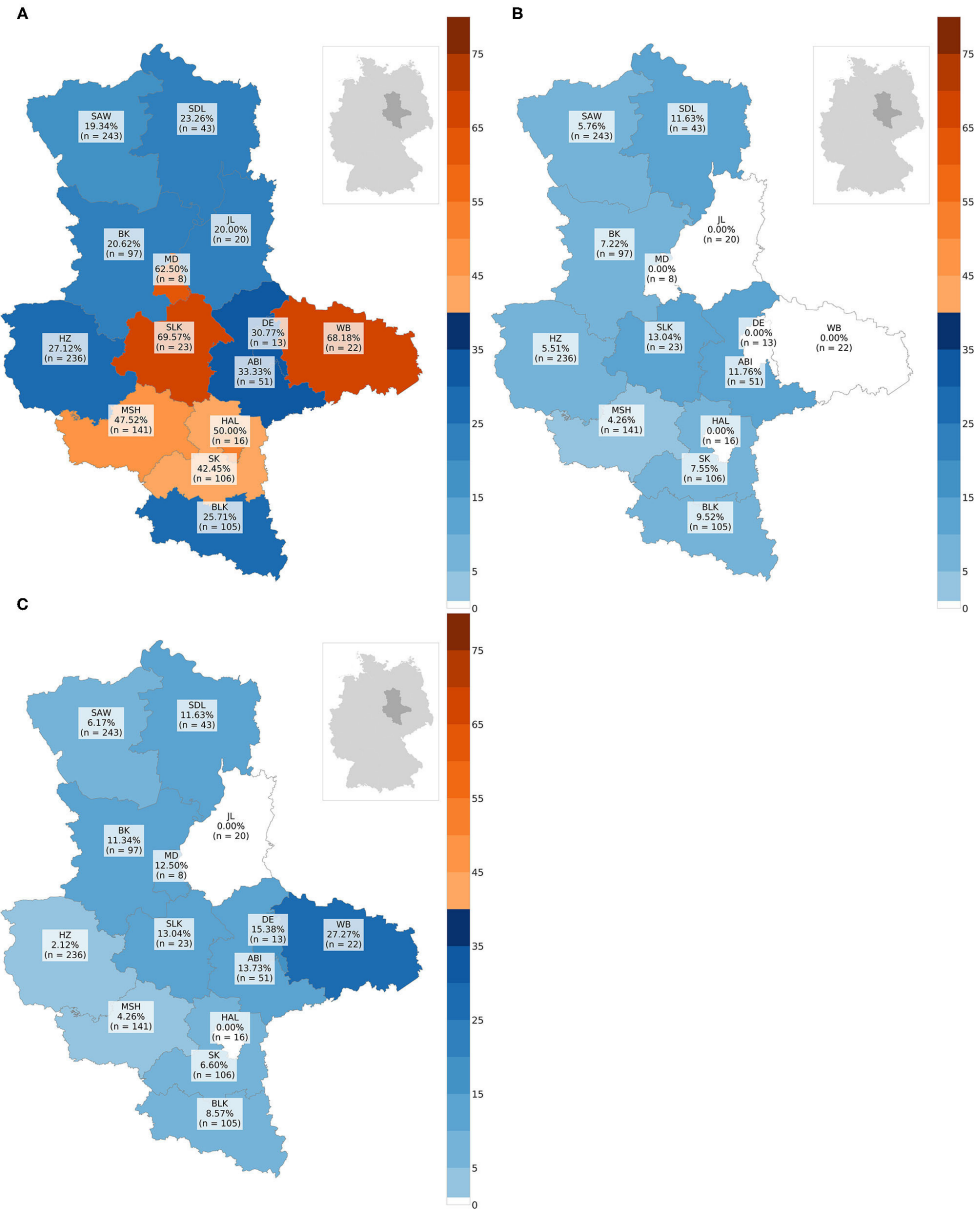
Maps with prevalences in % of every administrative district: **(A)** prevalence of CDV, **(B)** prevalence of CPV-2, **(C)** prevalence of FoxCV. Number of animals sent in (*n*); ABI, Landkreis Anhalt-Bitterfeld; BK, Landkreis Boerde; BLK, Burgenlandkreis; DE, Dessau-Rosslau; HAL, Halle (Saale); HZ, Landkreis Harz; JL, Landkreis Jerichower-Land; MD, Magdeburg; MSH, Landkreis Mansfeld-Suedharz; SAW, Altmarkkreis Salzwedel; SDL, Landkreis Stendal; SK, Landkreis Saalekreis; SLK, Salzlandkreis; WB, Landkreis Wittenberg.

In total, CDV was found in 349animals. The overall prevalence was 31.05% (349/1,124). Thus, CDV was the most frequently detected pathogen in all animal species, except in martens (Figure 3A). Species-independent prevalence in adults (338/1044; 32.38%) was significantly higher than in juveniles (11/80; 13.75%; χ2= 11.19; *p* = 0.0008; Figure 3B). There was no statistical significant difference between males (187/644; 29.04%) and females (162/480; 33.75%) neither across (χ2= 2.64; *p* = 0.1044) nor within each species (Figure 3C). Independent of species, the prevalence of CDV in spring (80/175; 45.71%) and winter (157/373; 42.09%) was significantly higher (χ2= 76.78; *p* < 0.0001) than in summer (34/211; 16.11%) and autumn (78/365; 21.37%; Figure 4A). The same was observed within each individual species, but only for red foxes and raccoons, there were significant differences between seasons (χ2= 62.44; *p* < 0.0001or χ2= 10.37; *p* = 0.0157, respectively). CDV was found in animals from all administrative districts with higher prevalences in the middle and south (Figure 5A).

Histopathological changes were noted in 205of the 349 CDV-positive animals (58.74%). None of these animals had inclusion bodies in the brain. In the remaining 144animals (41.26%), no pathomorphological changes were obvious.

#### 3.3.2. Canine Parvovirus Type 2

Altogether, CPV-2 and its antigenic variants were detected in 72animals. The overall prevalence was 6.41% (Figure 3A)with the highest value in raccoons (18/204; 13.73%; Figure 3A). Neither between adults (70/1044; 6.70%) and juveniles (2/80; 2.5%; χ2= 1.55; *p* = 0.2137; Figure 3B) nor between males (43/644; 6.68%) and females (29/480; 6.04%; χ2= 0.09; *p* = 0.7587; Figure 3C) there was a difference in prevalence of CPV-2 across species. The nucleic acid of CPV-2 was found only in adult animals, except in red foxes, where young animals were also affected. Prevalence was the highest in autumn (38/365; 10.41%, Figure 4B) independent of species. Similar results were seen in red foxes and raccoons if examined individually. In raccoon dogs and badgers, CPV-2 could only be detected in winter and in martens, only in summer. Here, due to the small number of animals, no significance test was performed. Low prevalences of CPV-2 were evident in affected administrative districts (Figure 5B).

Histopathological findings occurred in 23of the 72 CPV-2-positive animals (31.94%). In total, 49 animals (68.06%) had no pathomorphologically recognizable changes.

#### 3.3.3. Fox Circovirus

Fox circovirus was found in total in 77animals with an overall prevalence of 6.80% (Figure 3A). The highest prevalence value was obtained in raccoon dogs (4/34; 11.76%; Figure 3A). Neither across species nor within each species there was a statistically significant difference in prevalence between adults (72/1044; 6.90%) and juveniles (5/80; 6.25%; χ2= 0.00; *p* = 0.9928; Figure 3B). Among species, FoxCV could only be found in juvenile red foxes (Figure 3B). There were no differences (χ2= 0.11; *p* = 0.7414) between prevalences in males (46/644; 7.14%) and females (31/480; 6.46%; Figure 3C) independent of species. Prevalence was the highest in autumn (33/365; 9.04%; Figure 4C) across species. In contrast, the prevalence of FoxCV in red foxes was the highest in summer (14/130; 10.77%; Figure 4C) and in raccoon dogs and badgers, in winter (2/9; 22.22%and 1/3; 33.33%, respectively; Figure 4C). Differences were not significant (χ2= 4.65; *p* = 0.1992) except in red foxes between summer and winter (χ2= 4.02; *p* = 0.0449). The prevalence of FoxCV in administrative districts was low (Figure 5C).

Histopathological changes were seen in 42animals (54.55%) and absent in the remaining 35individuals (45.45%).

#### 3.3.4. Bacteria

Bacterial pathogens of different species were isolated from the brains of 20animals (1.78%; for more details, see **Table 4**): *Streptococcus canis* (*S. canis*) (6/20; 30.00%), *L. monocytogenes* (4/20; 20.00%), non-hemolytic *Escherichia coli* (*E. coli*) (3/20; 15.00%), *Salmonella enterica* (*S. enterica*) subsp. *enterica* (2/20; 10.00%), beta-hemolytic *E. coli* (1/20; 5.00%), *Pasteurella canis* (*P. canis*) (1/20; 5.00%), *S. enterica* subsp. *diarizonae* (1/20; 5.00%), *S. enterica* subsp. *enterica* ser. Enteritidis (1/20; 5.00%), and *Yersinia enterocolitica* (*Y. enterocolitica*) (1/20; 5.00%).

All animals were adults. Seven individuals were females (35.00%) and 13were males (65.00%). They originated from almost all administrative districts (Supplementary Table S1).

A suppurative meningoencephalitis indicating bacterial infection (13) was found in a red fox with *S. canis* (Figure 2F). All others either showed no histopathological changes (9/20; 45.00%) or changes suggestive of the concomitant presence of viral pathogens (9/20; 45.00%; **Table 3**).

#### 3.3.5. Parasites

Fifteen animals were investigated by IHC. ***T. gondii*** antigen (Figure 2D) was found in fourred foxes (4/15; 26.67%). Threewere adults and onejuvenile. Twowere males and twofemales. Animals originated from three administrative districts. The antigen of *T. gondii* was always detected in combination with the nucleic acid of viruses (details in **Table 4** and chapter 3.4.3). *N. caninum* was excluded using PCR.

**Nematode larvae** were found alone (*n* = 11) or in combination with CDV and/or CPV-2 (*n*=6) in the center of the granulomatous brain lesions (Figure 2E) of 17 adult animals (17/1,124; 1.51%). Of these, tenwere males and sevenwere females. Most of the animals originated from the middle (*n* = 5) or south (*n* = 11) of Saxony-Anhalt (Supplementary Table S1). All affected animals showed granulomatous inflammation, in two cases as mixed forms. Single animals with exclusive detection of nematode larvae showed additonal neuronal necrosis (*n* = 1) or vacuolization/demyelination (*n* = 3). For histopathological findings of animals with concomitant virus detection, see chapter 3.4.3.

### 3.4. Combinations of Pathogens and Corresponding Findings

More than one pathogen was detected in 68of the 1,124animals (6.05%). An overview of all combinations found is given in **Table 4**. For the purposes of better understanding, viruses, bacteria, and parasites are described separately in the following.

#### 3.4.1. Combinations of Viruses

Nucleic acids of more than one virus were evident in 46adults (4.09%). The combination of CDV and FoxCV was found most frequently (26/46; 56.52%), followed by the combination of CDV and CPV-2 (12/46; 26.09%), and last of CPV-2 and FoxCV (7/46; 15.22%). The combination of all three viruses occurred in a red fox (1/46; 2.17%). Detailed histopathological findings are listed in Table 2 and diagnoses for each individual animal are given in Supplementary Table S1.

**Table 2:** Histopathological findings in animals with the various combinations of canine distemper virus (CDV), canine parvovirus type 2 (CPV-2), and/or fox circovirus (FoxCV).

	**CDV/**	**CDV/**	**CPV-2/**	**CDV/CPV-2/**
	**FoxCV**	**CPV-2**	**FoxCV**	**FoxCV**
**Total number with histopathological changes**	16/26	6/12	6/7	1/1
**Inflammation**	13/16	6/6	4/6	1/1
Meningitis	6/13	3/6	3/4	
Meningoencephalitis	6/13	2/6		
Encephalitis	1/13	1/6	1/4	1/1
**Inflammation character**				
Non-suppurative	13/13	4/6	4/4	
Granulomatous		1/6		1/1
Mixed		1/6		
**Degree of inflammation**				
Minimal	5/13	1/6	1/4	
Mild	6/13	4/6	2/4	1/1
Marked	2/13	1/6	1/4	
**Localization of inflammation**				
Focal		2/6	1/4	
Multifocal	13/13	4/6	3/4	1/1
Affected brain area ^*^				
Cerebrum	10/13	5/6	3/4	1/1
Cerebellum	8/13	2/6	2/4	
Hippocampus	1/13	1/6	1/4	1/1
Brain stem	6/13	3/6		1/1
Other reactive changes ^**^	6/16	4/6	3/6	1/1
Gliosis	6/6	4/4	3/3	1/1
Satellitosis	2/6	1/4		
Neuronophagia	2/6	1/4		
Neuronal necrosis	1/6			
Other reactive changes with inflammation ^**^				
Gliosis	4/6	4/4	1/3	1/1
Satellitosis	1/2	1/1		
Neuronophagia	1/2	1/1		
Neuronal necrosis	1/1			
Degenerative changes ^**^	7/16	3/6		
Vacuolization/demyelination	7/7	3/3		
Degenerative changes with inflammation ^**^				
Vacuolization/demyelination	5/7	3/3		
**Combination of other reactive and degenerative changes**	4/16	2/6		
With inflammation	3/4	2/2		
**Total number without histopathological changes**	10/26	6/12	1/7	

*The numbers listed refer to affected animals*.

Combinations of different affected brain areas possible (^*^), combinations of different findings possible (^**^).

Of the 26 **CDV- and FoxCV**-positive animals, 13exhibited inflammation (13/26; 50.00%). Threeother (3/26; 11.54%) individuals showed gliosis, satellitosis, and neuronophagia (*n* = 1); vacuolization/demyelination (*n* = 1); and gliosis and vacuolization/demyelination (*n* = 1), respectively. In tenanimals (10/26; 38.46%), we did not find histopathological changes.

The combination of **CDV and CPV-2** was found in 12individuals (12/46; 26.09%). In sixof them (50.00%), inflammation was noted, usually combined with gliosis, satellitosis, neuronophagia, and/or vacuolization/demyelination. No histopathological changes were detected in the remaining six (50.00%).

In seven of the 46animals (7/46; 15.22%), we detected **CPV-2 and FoxCV**. Sixout of these (85.71%) exhibited histopathological changes. Inflammation was found in fourof them, one had additional gliosis. Twoanimals showed only gliosis. The last one did not show any histopathological changes.

A red fox (1/46; 2.17%) was even carrier of three viruses (**CDV**, **CPV-2, and FoxCV**). Histopathological examination revealed encephalitis with gliosis.

#### 3.4.2. Bacteria in Combination With Viruses

In eightof the 20bacteriological positive animals (40.00%), we detected bacteria as single agents [*S. canis* (*n* = 4), non-hemolytic *E. coli* (*n* = 3), and *S. enterica* subsp. *diarizonae* (*n* = 1)]. The 12other animals (60.00%) were additionally positive for CDV (*n* = 9), CDV and FoxCV (*n* = 2), or FoxCV (*n* = 1; **Table 4** and Supplementary Table S1). All of these animals had no changes in the brain typical for a manifest bacterial infection (13). Histopathological findings are listed in Table 3.

**Table 3:** Histopathological findings in animals with the combinations of bacteria or parasites and canine distemper virus (CDV), canine parvovirus type 2 (CPV-2), and/or fox circovirus (FoxCV).

	**Bacteria ^a^/**	**Bacteria ^b^/**	**Bacteria ^c^/**	***T. gondii*/**	***T. gondii*/**	**Nematode larvae/**	**Nematode larvae/**
	**CDV**	**CDV/FoxCV**	**FoxCV**	**CDV**	**CDV/FoxCV**	**CDV**	**CDV/CPV-2**
**Total number with histopathological changes**	7/9	2/2		2/2	2/2	5/5	1/1
**Inflammation**	6/7	1/2		2/2	2/2	5/5	1/1
Meningitis	3/6						
Meningoencephalitis	2/6	1/1			2/2		
Encephalitis	1/6			2/2		5/5	1/1
**Inflammation character**							
Non-suppurative	6/6	1/1					
Granulomatous				2/2		5/5	1/1
Mixed					2/2		
**Degree of inflammation**							
Minimal	3/6				1/2		
Mild	3/6			1/2	1/2	5/5	1/1
Marked		1/1		1/2			
**Localization of inflammation**							
Focal	1/6					4/5	1/1
Multifocal	5/6	1/1		2/2	2/2	1/5	
Affected brain area ^*^							
Cerebrum	5/6	1/1		2/2	2/2	3/5	1/1
Cerebellum	3/6			1/2	2/2	3/5	
Hippocampus				1/2	1/2		
Brain stem	1/6			2/2	1/2	1/5	
Other reactive changes ^*^	4/7	2/2		2/2		2/5	
Gliosis	4/4	2/2		2/2		2/2	
Satellitosis		1/2		1/2			
Neuronophagia		1/2					
Neuronal necrosis		1/2					
Other reactive changes with inflammation ^**^							
Gliosis	3/4	1/2		2/2		2/2	
Satellitosis				1/1			
Degenerative changes ^**^	2/7				1/2	3/5	1/1
Vacuolization/demyelination	2/2				1/1	3/3	1/1
Degenerative changes with inflammation ^**^							
Vacuolization/demyelination	1/2				1/1	3/3	1/1
**Combination of other reactive and degenerative changes**	2/7					2/5	
With inflammation	1/2					2/2	
**Total number without histopathological changes**	2/9		1/1				

*The numbers listed refer to affected animals*.

Combinations of different affected brain areas possible (^*^), combinations of different findings possible (^**^), bacteria including Listeria monocytogenes, Salmonella enterica subsp. enterica, Salmonella enterica subsp. enterica ser. Enteritidis, Streptococcus canis, beta-hemolytic Escherichia coli or Pasteurella canis (^a^), bacteria including L. monocytogenes or S. canis (^b^), bacteria including Yersinia enterocolitica (^c^).

*Listeria monocytogenes* (*n* = 4) was detected in combination with CDV in threered foxes and with CDV and FoxCV in oneraccoon.

Both, *S. enterica* subsp. *enterica* (2/12; 16.67%) and *S. enterica* subsp. *enterica* ser. Enteritidis (1/12; 8.33%) were found in combination with CDV in red foxes.

The two red foxes with *S. canis* had simultaneously either an infection with CDV (1/12; 8.33%) or with CDV and FoxCV (1/12; 8.33%).

Beta-hemolytic *E. coli* and CDV (1/12; 8.33%) were present in only one raccoon dog.

Furthermore, a red fox was simultaneously infected with *P. canis* and CDV (1/12; 8.33%) and another with *Y. enterocolitica* and FoxCV (1/12; 8.33%).

#### 3.4.3. Parasites in Combination With Viruses

All four red foxes with the presence of ***T. gondii*** antigen (Figure 2D) additionally were positively tested for the nucleic acid of at least one virus (Table 4). Two were positive for CDV, one an adult, the other a juvenile. Another two adults were positive for CDV and FoxCV. Histopathologically, all individuals showed a granulomatous inflammation and other findings typical of viral infection (see Table 3).

**Table 4:** Animals with detected infectious pathogens including combinations of different pathogens.

**Pathogen**	**Red Fox (*****n*** = **860)**		**Raccoon (*****n*** = **204)**		**Raccoon Dog (*****n*** = **34)**		**Marten (*****n*** = **14)**		**Badger (*****n*** = **12)**		**Total (*****n*** = **1,124)**	
	**Samples**	**in % (CI)**	**Samples**	**in % (CI)**	**Samples**	**in % (CI)**	**Samples**	**in % (CI)**	**Samples**	**in % (CI)**	**Samples**	**in % (CI)**
CDV	257	29.88 (26.84–33.07)	24	11.76 (7.69–17.00)	5	14.71 (4.95–31.06)	0	0.00 (0.00–23.16)	3	25.00 (5.49–57.19)	289	25.71 (23.18–28.37)
CPV-2	24	2.79 (1.80–4.12)	24	11.76 (7.69–17.00)	1	2.94 (0.07–15.33)	1	7.14 (0.18–33.87)	1	8.33 (0.21–38.48)	51	4.54 (3.40–5.92)
FoxCV	32	3.72 (2.56–5.21)	3	1.47 (0.30–4.24)	3	8.82 (1.86–23.68)	0	0.00 (0.00–23.16)	0	0.00 (0.00–26.46)	38	3.38 (2.40–4.61)
Nonhemolytic *E. coli*	2	0.23 (0.03–0.84)	1	0.49 (0.01–2.70)	0	0.00 (0.00–10.28)	0	0.00 (0.00–23.16)	0	0.00 (0.00–26.46)	3	0.27 (0.06–0.78)
*S. enterica* subsp. *diarizonae*	1	0.12 (0.00–0.65)	0	0.00 (0.00–1.79)	0	0.00 (0.00–10.28)	0	0.00 (0.00–23.16)	0	0.00 (0.00–26.46)	1	0.09 (0.00–0.49)
*S. canis*	2	0.23 (0.03–0.84)	1	0.49 (0.01–2.70)	0	0.00 (0.00–10.28)	1	7.14 (0.18–33.87)	0	0.00 (0.00–26.46)	4	0.36 (0.10–0.91)
Larvae of nematodes	9	1.05 (0.48–1.98)	1	0.49 (0.01–2.70)	1	2.94 (0.07–15.33)	0	0.00 (0.00–23.16)	0	0.00 (0.00–26.46)	11	0.98 (0.49–1.74)
**Total single pathogen**	327	38.02 (34.77–41.36)	54	26.47 (20.55–33.08)	10	29.41 (15.10–47.48)	2	14.29 (1.78–42.81)	4	33.33 (9.92–65.11)	397	35.32 (32.52–38.19)
CDV, CPV-2	8	0.93 (0.40–1.82)	4	1.96 (0.54–4.94)	0	0.00 (0.00–10.28)	0	0.00 (0.00–23.16)	0	0.00 (0.00–26.46)	12	1.07 (0.55–1.86)
CDV, FoxCV	22	2.56 (1.61–3.85)	2	0.98 (0.12–3.50)	1	2.94 (0.07–15.33)	0	0.00 (0.00–23.16)	1	8.33 (0.21–38.48)	26	2.31 (1.52–3.37)
CPV-2, FoxCV	7	0.81 (0.33–1.67)	0	0.00 (0.00–1.79)	0	0.00 (0.00–10.28)	0	0.00 (0.00–23.16)	0	0.00 (0.00–26.46)	7	0.62 (0.25–1.28)
CDV, beta-hemolytic *E. coli*	0	0.00 (0.00–0.43)	0	0.00 (0.00–1.79)	1	2.94 (0.07–15.33)	0	0.00 (0.00–23.16)	0	0.00 (0.00–26.46)	1	0.09 (0.00–0.49)
CDV, *L. monocytogenes*	3	0.35 (0.07–1.02)	0	0.00 (0.00–1.79)	0	0.00 (0.00–10.28)	0	0.00 (0.00–23.16)	0	0.00 (0.00–26.46)	3	0.27 (0.06–0.78)
CDV, *P. canis*	1	0.12 (0.00–0.65)	0	0.00 (0.00–1.79)	0	0.00 (0.00–10.28)	0	0.00 (0.00–23.16)	0	0.00 (0.00–26.46)	1	0.09 (0.00–0.49)
CDV, *S. enterica* subsp. *enterica*	2	0.23 (0.03–0.84)	0	0.00 (0.00–1.79)	0	0.00 (0.00–10.28)	0	0.00 (0.00–23.16)	0	0.00 (0.00–26.46)	2	0.18 (0.02–0.64)
CDV, *S. enterica* subsp. *enterica* ser. Enteritidis	1	0.12 (0.00–0.65)	0	0.00 (0.00–1.79)	0	0.00 (0.00–10.28)	0	0.00 (0.00–23.16)	0	0.00 (0.00–26.46)	1	0.09 (0.00–0.49)
CDV, *S. canis*	1	0.12 (0.00–0.65)	0	0.00 (0.00–1.79)	0	0.00 (0.00–10.28)	0	0.00 (0.00–23.16)	0	0.00 (0.00–26.46)	1	0.09 (0.00–0.49)
FoxCV, *Y. enterocolitica*	1	0.12 (0.00–0.65)	0	0.00 (0.00–1.79)	0	0.00 (0.00–10.28)	0	0.00 (0.00–23.16)	0	0.00 (0.00–26.46)	1	0.09 (0.00–0.49)
CDV, *T. gondii*	2	0.23 (0.03–0.84)	0	0.00 (0.00–1.79)	0	0.00 (0.00–10.28)	0	0.00 (0.00–23.16)	0	0.00 (0.00–26.46)	2	0.18 (0.02–0.64)
CDV, larvae of nematodes	4	0.47 (0.13–1.19)	1	0.49 (0.01–2.70)	0	0.00 (0.00–10.28)	0	0.00 (0.00–23.16)	0	0.00 (0.00–26.46)	5	0.44 (0.14–1.04)
**Total two pathogens**	52	6.05 (4.55–7.85)	7	3.43 (1.39–6.94)	2	5.88 (0.72–19.68)	0	0.00 (0.00–23.16)	1	8.33 (0.21–38.48)	62	5.52 (4.25–7.02)
CDV, CPV-2, FoxCV	1	0.12 (0.00–0.65)	0	0.00 (0.00–1.79)	0	0.00 (0.00–10.28)	0	0.00 (0.00–23.16)	0	0.00 (0.00–26.46)	1	0.09 (0.00–0.49)
CDV, FoxCV, *L. monocytogenes*	0	0.00 (0.00– 0.43)	1	0.49 (0.01–2.70)	0	0.00 (0.00–10.28)	0	0.00 (0.00–23.16)	0	0.00 (0.00–26.46)	1	0.09 (0.00– 0.49)
CDV, FoxCV, *S. canis*	1	0.12 (0.00– 0.65)	0	0.00 (0.00– 1.79)	0	0.00 (0.00– 10.28)	0	0.00 (0.00–23.16)	0	0.00 (0.00–26.46)	1	0.09 (0.00–0.49)
CDV, FoxCV, *T. gondii*	2	0.23 (0.03– 0.84)	0	0.00 (0.00–1.79)	0	0.00 (0.00–10.28)	0	0.00 (0.00–23.16)	0	0.00 (0.00–26.46)	2	0.18 (0.02–0.64)
CDV, CPV- 2, larvae of nematodes	1	0.12 (0.00– 0.65)	0	0.00 (0.00–1.79)	0	0.00 (0.00–10.28)	0	0.00 (0.00–23.16)	0	0.00 (0.00–26.46)	1	0.09 (0.00–0.49)
**Total three pathogens**	5	0.58 (0.19–1.35)	1	0.49 (0.01–2.70)	0	0.00 (0.00–10.28)	0	0.00 (0.00–23.16)	0	0.00 (0.00–26.46)	6	0.53 (0.20–1.16)
Negative	476	55.35 (51.95–58.71)	142	69.61 (62.80–75.84)	22	64.71 (46.49–80.25)	12	85.71 (57.19–98.22)	7	58.33 (27.67–84.83)	659	58.63 (55.69–61.53)

*Number of animals sent in (n), 95% confidence interval (CI)*.

**Nematode larvae** in combination with viral nucleic acid were detected in sixof 17animals (35.29%, Table 4). Five animals had CDV (4 red foxes, 1 raccoon) and one red fox had CDV and CPV-2. All of them showed larval granulomas, with additional changes of viral infection in four of them. The remaining two exhibited no further histopathological changes (refer to Table 3).

### 3.5. Pathogen Discovery Correlated With Histopathological Findings

In 659animals, we failed to detect infectious pathogens with the methods used (659/1,124; 58.63%, see Table 5). Of these animals, 467 (70.86%) were completely considered negative, we neither found infectious pathogens nor histomorphological changes indicating an infection.

**Table 5:** Pathogen discovery correlated with main histopathological findings in each animal species.

**Pathogen findings**	**Main histopathological findings**	**Red Fox (*****n*** = **860)**		**Raccoon (*****n*** = **204)**		**Raccoon Dog (*****n*** = **34)**		**Marten (*****n*** = **14)**		**Badger (*****n*** = **12)**		**Total (*****n*** = **1,124)**	
		**Samples**	**in % (CI)**	**Samples**	**in % (CI)**	**Samples**	**in % (CI)**	**Samples**	**in % (CI)**	**Samples**	**in % (CI)**	**Samples**	**in % (CI)**
Negative	Inflammation	47	5.47 (4.04–7.20)	10	4.90 (2.38–8.83)	5	14.71 (4.95–31.06)	2	14.29 (1.78–42.81)	1	8.33 (0.21-38.48)	65	5.78 (4.49–7.31)
	Inflammation and other reactive changes *^a^*	24	2.79 (1.80–4.12)	5	2.45 (0.80–5.63)	1	2.94 (0.07–15.33)	2	14.29 (1.78–42.81)	0	0.00 (0.00–26.46)	32	2.85 (1.96–4.00)
	Inflammation and degenerative changes *^b^*	14	1.63 (0.89–2.72)	2	0.98 (0.12–3.50)	0	0.00 (0.00–10.28)	0	0.00 (0.00–23.16)	0	0.00 (0.00–26.46)	16	1.42 (0.82–2.30)
	Inflammation, other reactive *^a^* and degenerative changes *^b^*	12	1.40 (0.72–2.42)	2	0.98 (0.12–3.50)	0	0.00 (0.00–10.28)	0	0.00 (0.00–23.16)	0	0.00 (0.00–26.46)	14	1.25 (0.68–2.08)
	Other reactive changes *^a^*	14	1.63 (0.89–2.72)	5	2.45 (0.80–5.63)	1	2.94 (0.07–15.33)	0	0.00 (0.00–23.16)	0	0.00 (0.00–26.46)	20	1.78 (1.09–2.73)
	Degenerative changes *^b^*	24	2.79 (1.80–4.12)	6	2.94 (1.09–6.29)	0	0.00 (0.00–10.28)	0	0.00 (0.00–23.16)	0	0.00 (0.00–26.46)	30	2.67 (1.81–3.79)
	Other reactive *^a^* and degenerative changes *^b^*	11	1.28 (0.64–2.28)	3	1.47 (0.30–4.24)	0	0.00 (0.00–10.28)	0	0.00 (0.00–23.16)	1	8.33 (0.21–38.48)	15	1.33 (0.75–2.19)
	No significant findings	330	38.37 (35.11–41.72)	109	53.43 (46.33–60.43)	15	44.12 (27.19–62.11)	8	57.14 (28.86–82.34)	5	41.67 (15.17–72.33)	467	41.55 (38.65–44.49)
Positive	Inflammation	59	6.86 (5.26–8.76)	3	1.47 (0.30–4.24)	2	5.88 (0.72–19.68)	1	7.14 (0.18–33.87)	1	8.33 (0.21–38.48)	66	5.87 (4.57–7.41)
	Inflammation and other reactive changes *^a^*	49	5.70 (4.24–7.46)	8	3.92 (1.71–7.58)	3	8.82 (1.86–23.68)	0	0.00 (0.00–23.16)	2	16.67 (2.09–48.41)	62	5.52 (4.25–7.02)
	Inflammation and degenerative changes *^b^*	29	3.37 (2.27–4.81)	0	0.00 (0.00–1.79)	1	2.94 (0.07–15.33)	0	0.00 (0.00–23.16)	0	0.00 (0.00–26.46)	30	2.67 (1.81–3.79)
	Inflammation, other reactive *^a^* and degenerative changes *^b^*	30	3.49 (2.37–4.94)	4	1.96 (0.54–4.94)	1	2.94 (0.07–15.33)	0	0.00 (0.00–23.16)	0	0.00 (0.00–26.46)	35	3.11 (2.18–4.30)
	Other reactive changes *^a^*	13	1.51 (0.81–2.57)	4	1.96 (0.54–4.94)	2	5.88 (0.72–19.68)	0	0.00 (0.00–23.16)	0	0.00 (0.00–26.46)	19	1.69 (1.02–2.63)
	Degenerative changes *^b^*	21	2.44 (1.52–3.71)	2	0.98 (0.12–3.50)	0	0.00 (0.00–10.28)	0	0.00 (0.00–23.16)	0	0.00 (0.00–26.46)	23	2.05 (1.30–3.05)
	other reactive *^a^* and degenerative changes *^b^*	12	1.40 (0.72–2.42)	1	0.49 (0.01–2.70)	0	0.00 (0.00–10.28)	0	0.00 (0.00–23.16)	0	0.00 (0.00–26.46)	13	1.16 (0.62–1.97)
	No significant findings	171	19.88 (17.26–22.71)	40	19.61 (14.39–25.73)	3	8.82 (1.86–23.68)	1	7.14 (0.18–33.87)	2	16.67 (2.09–48.41)	217	19.31 (17.04–21.74)

*Number of animals sent in (n), 95% confidence interval (CI), single occurrence or various combinations of gliosis, satellitosis, neuronophagia, and/or neuronal necrosis possible (^a^), single occurrence or various combinations of vacuolization/demyelination and/or malacia possible (^b^)*.

On the other hand, 192 of the 659 “pathogen-negative” animals (18.15%) showed histopathological changes. Inflammatory processes which were non-suppurative (118/127; 92.91%), eosinophilic (1/127; 0.79%; Figure 2G), or mixed (8/127; 6.30%) were diagnosed in 127 of the 192animals (66.15%). Furthermore, these findings were combined with gliosis, satellitosis, neuronophagia, neuronal necrosis, and/or vacuolization/demyelination.

No inflammatory processes were observed in 65 of the 192animals (33.85%). Here, we diagnosed different combinations of gliosis, satellitosis, neuronophagia, neuronal necrosis, and/or vacuolization/demyelination.

In contrast, in 217 of the 465animals (46.67%) with pathogens detected in our study, no histopathological changes were found in the brain sections (Table 5).

## 4. Discussion

In this study, we examined 1,124animals to get an overview of the occurrence of (zoonotic) pathogens that may cause diseases in the brain of wild carnivores and may pose a risk to humans, wild, domestic, and zoo animals.

We found CDV, FoxCV, CPV-2, *T. gondii*, nematode larvae, *L. monocytogenes*, and additionally other bacterial pathogens. All animals were negative for RABV, WNV, BoDV-1, SuHV-1, CaHV-1, and *N. caninum*.

With almost one-third of the **CDV**-positive animals (349/1,124; 31.05%) in our study, this observation agrees with the results of other German authors (2, 43, 44). In contrast, in Schleswig-Holstein among wild carnivores tested by IHC, not a single positive animal was found (45). Thus, prevalences seem to differ significantly due to different sampling periods, test procedures, and geographic regions. Animal and human population density varies in different geographies due to the fact, that most wild carnivores as so-called synanthropic species prefer to colonize urban regions (20, 22, 46).

Species-independent, we found CDV in more adult animals than in juveniles. Restrictively, it should be mentioned that in our study way more adults than juveniles were submitted and tested.

Consistent with the literature (47, 48) and our data, gender does not appear to have a major impact on CDV infection. Furthermore, this is also true for CPV-2 and FoxCV in our study as well as in studies of other authors (10, 48–50).

During our investigations on CDV, independent of species, especially those animals that were submitted in winter (overall prevalence 42.1%) and spring (overall prevalence 38.2%) were mainly affected which could be due to mating season (51). During this time, especially red foxes and raccoons as solitary living animals have more contact with one another and may become infected (2, 3, 52). Furthermore, in autumn and winter, juveniles could become more susceptible to infection because of loss of maternal antibodies during the first three months of life (53) and increased movement with the objective to find new territories (51). In winter and spring, reduced availability of food sources could weaken the animals and make them more susceptible to infections.

In agreement with Denzin et al. (43), we also found an inhomogeneous distribution of CDV in the administrative districts of Saxony-Anhalt.

In more than half of the CDV-positive animals in our study, histopathological changes had occured in the brains. Most frequently, we found a non-suppurative meningoencephalitis, most often combined with findings also described in literature on the nervous form (35, 54–57). However, inclusion bodies were not detected in our study animals.

Accordingly, the wide distribution of CDV poses a risk to dogs and other susceptible animals, as the virus can be easily transmitted from infected wild carnivores (3, 55).

In our study, 72animals were positive for **CPV-2** and its antigenic variants. Evidence of CPV-2 in wildlife populations in Germany was also obtained in other studies (58, 59).

About 6% of all examined animals in our study were positive for CPV-2-DNA, raccoons were significantly higher with 13.73%. This is the first description of CPV-2 in wild carnivores in Saxony-Anhalt. In the brain, neither Bourg et al. (44) nor Lempp et al. (45) could detect CPV-2-antigen in wild carnivores in Germany by IHC. Restrictively, it must be said that due to the non-standardized sample materials and different test methods, the prevalences in the literature and our results cannot be directly compared with each other. However, our data indicate a virus circulation in wild carnivores in Saxony-Anhalt.

During our study, CPV-2 was most often detected in animals that were submitted in autumn (red foxes and raccoons) and winter (raccoon dogs and badgers). This could be due to waning of maternal antibody titers in juveniles (60). Nevertheless, in our investigations, only a few animals were positive for CPV-2 at all. Thus, it is difficult to draw final conclusions about a possible influence of age or season.

Nearly one-third of the CPV-2-positive animals of our study showed histopathological changes in the brain. Mostly these were non-suppurative meningitis or meninogoencephalitis. There were often combinations with gliosis, satellitosis, neuronophagia, and/or vacuolization/demyelination. These findings are in agreement with other studies in young dogs and a cat infected with CPV-2 (9, 61).

During our examination, we detected **FoxCV** in the brains of about 7% of the animals. FoxCV and the closely related dog circovirus (DogCV) (62) have both been described mainly in dogs, but also in red foxes, arctic foxes, wolves, and badgers (49, 50). Furthermore, the virus has occasionally been observed together with other viruses, such as CPV-2, CDV, or other pathogens (63–69). Thus, it is inconclusive whether the symptoms described were caused by FoxCV/DogCV alone, by the other pathogens, or due to immunosuppression by FoxCV/DogCV and subsequent enhancement of the effect of the other pathogens.

We detected FoxCV in the brains of 77animals. As to our knowledge, this is the first description of FoxCV in Germany. In Europe, the prevalence in wild carnivores ranges from 0 to 76.5% (10, 49, 50, 70). This may be the result of different test methods, samples, and geographic regions.

In our study, no differences in prevalence were observed in adult (6.90%) and juvenile (6.25%) animals. However, due to the low numbers of FoxCV-positive animals here, a possible influence of age could not be determined.

Most often, we detected the pathogen in autumn, independent of species. When species were considered individually, in red foxes, FoxCV was detected most often in summer and in raccoon dogs and badgers, most often in winter. There are no data available on the seasonality of the virus.

In Saxony-Anhalt, the pathogen was not found in all administrative districts, and if so, the prevalence was low. In order to gain further knowledge about the epidemiology in wild and domestic animals, further and especially long-term studies are required.

In half of the FoxCV-positive animals, we observed most often non-suppurative inflammation in the brain, frequently combined with gliosis, satellitosis, neuronophagia, neuronal necrosis, and/or vacuolization/demyelination. Bexton et al. (10) reported similar findings. Based on the limited data available, it remains elusive whether the brain or other organs show morphological changes during FoxCV infection. Notably, the other half of the FoxCV-positive animals did not show histomorphological brain lesions.

During our study, in addition to viruses, the animals were also examined for the presence of **bacteria**. In nine of 20animals, we isolated bacteria with zoonotic potential that can pose a possible threat to human health. Almost all of these nine were also positive for viral nucleic acid (*L. monocytogenes* and CDV *n* = 3; *L. monocytogenes*, CDV and FoxCV *n* = 1; *S. enterica* subsp. *enterica* and CDV *n* = 3; *S. enterica* subsp. *diarizonae n* = 1; *Y. enterocolitica* and FoxCV *n* = 1). Foxes and other wild carnivores are reservoirs of zoonotic bacterial pathogens and can be involved in their epidemiology, mostly as subclinical carriers (71–74). In Poland, in wild carnivores, *L. monocytogenes, Salmonella* spp., and *Y. enterocolitica* were detected (75). Considering literature data, our findings also demonstrate that wild carnivores in Saxony-Anhalt are indeed carriers of these pathogens and apparently can spread them.

Cases with typical listerial meningoencephalitis caused by *L. monocytogenes* as reported in raccoon dogs or cougars (76, 77) were not found in our animals. Therefore, the detection of *L. monocytogenes* seems to be only an incidental finding here. Our results are more likely to support lesions caused by viruses which we found simultaneously.

Animals that were positive for the other bacteria may have shown either no histopathological changes due to septicemia with sudden death or non-suppurative inflammation due to a concurrent CDV and/or FoxCV infection. Furthermore, either the infections had not progressed far enough or the lesions were in another area of the brain not examined by us. Another explanation could be contamination during brain preparation. Most of the bacteria found are ubiquitous in the environment or are commensals and therefore could have been attached to the furs (11, 73, 74, 78–80). Here, further investigations of other organs would have been necessary to confirm generalized bacterial infections. However, those were not included in our study design.

*Streptococcus canis* was detected in one animal with typical suppurative meningoencephalitis (80, 81). Similar cases were described in dogs (82). No case of streptococcal meningoencephalitis has been reported in wild carnivores in the literature.

Infections with ***T. gondii***, a zoonotic protozoan in wild carnivores, are common with prevalences ranging from 1.32 to 100%, as those are the intermediate hosts (83–87). In this study, the antigen was found in only four animals using IHC. All affected animals were additionally positive for CDV and/or FoxCV. The detection of *T. gondii* antigen is in line with data from a previous study in Saxony-Anhalt (84).

Further parasitic organisms found in 17animals were identified as **nematode larvae** in granulomatous inflammations. During somatic migration, they can cause cerebrospinal nematodiasis with granulomatous, or eosinophilic (meningo-)encephalitis, or hemorrhagic malacia in the central nervous system (13, 88–91). Nematode species may include larvae of *Baylisascaris procyonis* (92, 93), *Angiostrongylus vasorum* (88–90), or ascarid larvae (91). Since no histopathological determination of the species was performed in our study, the larval granulomas were most likely an incidental finding.

In our study, **combinations of infectious pathogens** occurred in 68cases. As a known immunosuppressive agent, CDV was involved in 60of these 68cases (55, 94). In wild carnivores, opportunistic infections of CDV with various pathogens are described in the literature, for example, with FoxCV (49) and/or CPV-2 (49), *L. monocytogenes* (76), *T. gondii* (14), and *E. coli* (95). Therefore, CDV may have been the agent that paved the way for infection with other pathogens or may have progressed the disease. Furthermore, ubiquitous bacteria and/or parasites are also potential secondary pathogens (11, 96, 97).

During our examinations, combinations of other pathogens occurred in the minority of cases. In contrast to previous studies, the combination of CPV-2/FoxCV was noticeable here due to the fact that six out of seven positive animals showed histopathological changes. Keeping in mind that FoxCV and DogCV are closely related, Li et al. (63) reported that DogCV-positive animals were co-infected with various other pathogens. Other authors described DogCV in combination with CDV and CPV-2 in wolves, dogs, and badgers (49) and mentioned a potential immunosuppressive effect of circoviruses (49, 63). Anderson et al. (65) described co-infections of CPV-2 and canine circovirus in dogs. The authors assumed that a CPV-2 infection can be a predisposing factor for a canine circovirus infection and may lead to more severe disease, similarly to porcine circovirus 2 in pigs (98). Thus, the importance of FoxCV is still unclear and should be addressed by further studies.

The results congest that our study has **limitations**. In nearly 40% of our cases, histopathological changes were found. On the one hand, in 192 of the 659animals, we observed histopathological findings without pathogen detection. This is consistent with other studies (44, 45). Various reasons should be taken into account. First, we screened the carnivores only for the presence of selected pathogens. There are much more infectious agents which can cause meningitis and/or encephalitis, for example, tick-borne encephalitis virus (99), canine adenovirus (100), canine parainfluenza virus (101), or *Encephalitozoon cuniculi* (102). In domestic carnivores, predominantly dogs, non-infectious causes of meningitis and/or encephalitis are also described, for example, canine necrotizing meningoencephalitis, granulomatous meningoencephalomyelitis (103), and idiopathic eosinophilic meningoencephalitis (13). However, not only in inflammation but also cases with vacuolization may be of non-infectious origin (33, 104). Second, some of the animals showed postmortem and freezing artifacts (*n* = 240) at the time of the investigation. This could possibly have complicated or limited the detection of specific pathogens (49). Third, for some of the pathogens chosen, as WNV, a short viremia with just a few days and with low titers has been described (105). As a result, viral RNA might not have been reliably detected. In addition, WNV was apparently not present in Germany until the first detection in 2018 (106), the year following our sampling. Furthermore, we did not use more generic methods, like next-generation sequencing in combination with metagenomic analysis, which may have identified the cause of meningitis/encephalitis in these so-called “unclear” cases. This could be part of further investigations.

On the other hand, several CDV-, CPV-2- and FoxCV-positive cases and animals with combined infections, respectively, lacked histopathological changes (*n* = 211). Either this may have been in the viremic phase of the infection (107–109) or the infection has already been cleared despite positive nucleic acid detection (108, 110, 111).

The diagnostic value of histopathology alone is limited. We did not examine serial brain sections. Thus, changes in the adjacent tissue might be overseen. This is also true for the selection of certain brain areas. Furthermore, without using special methods, we cannot distinguish in all cases between true pathologic changes and reproducible histotechnological artifacts as described for vacuolization by Wohlsein et al. (33). Moreover, we cannot exclude that some of the reactive changes, such as gliosis and satellitosis may only be incidental findings (33). Additionally, especially in the inflammatory areas, a coexistence of neuronal necrosis and neuronophagia might be masked. Also, the possibility cannot be excluded that in animals with diagnosed satellitosis already transitions to neuronophagia with early stages of neuronal degeneration were present but not detected by us using HE-stain only.

For the differentiation of local or systemic infections, the sole examination of the brain with neglection of other organs limits the diagnostic value. Besides, it is impossible to draw a conclusion on the causative agent(s) based on the histopathological findings alone. If viruses were found, in general, a non-suppurative inflammation possibly combined with gliosis, satellitosis, neuronophagia, neuronal necrosis, and/or vacuolization/demyelination, or malacia could be observed. Sometimes the non-inflammatory changes simply occurred alone. However, granulomatous inflammation was diagnosed only in the presence of *T. gondii* antigen, nematode larvae, and/or when CDV was involved.

Furthermore, especially in the case of *T. gondii* with only few tissue cysts, a combination of histopathology/IHC with serological data in future studies is reasonable. Based on the fact that serology is a more sensitive method for detecting carrier animals (112).

In summary, if no structures of pathogenic agents were detected, the histomorphological pattern could neither be used to draw conclusions on a specific pathogen nor could the virus be used to draw conclusions on a specific histopathological pattern.

In **conclusion**, this is the first study in Saxony-Anhalt involving more than 1,000 wild carnivores investigating different infectious agents (viruses, bacteria, and parasites) that can potentially cause meningitis/encephalitis. CDV was most frequently detected followed by CPV-2 and FoxCV, the latter being detected for the first time in Germany. However, further investigations are necessary to prove if FoxCV is a truly independent disease-causing pathogen or a cofactor for other pathogens.

In addition, we identified pathogens with potential zoonotic risk which can be a threat to humans, susceptible domestic and zoo animals, or the wildlife population. In our samples, RABV, SuHV-1, WNV, and BoDV-1 were not identified, but monitoring programs and further studies are required to investigate the role of wild carnivores in the epidemiology of specific pathogens, especially for WNV or BoDV-1.

Bacteria with zoonotic potential, such as *L. monocytogenes, Salmonella* spp. and *Yersina* spp., *T. gondii*, and nematode larvae have also been found in wild carnivores. Since these pose a particularly high risk to vulnerable people, but also persons with close contact to animals, such as hunters or farmers (113), the inclusion of these pathogens in a monitoring program would be advisable, especially considering that these pathogens can often be transmitted fecal-orally and thus contaminate the environment (72–74, 83). This would not only allow a more accurate assessment of the risk posed by infected wild carnivores but also estimate potential contamination of the surrounding environment.

## Data Availability Statement

The raw data supporting the conclusions of this article will be made available by the authors, without undue reservation.

## Author Contributions

CE, JH, and MP designed the study. JH performed the histopathological examinations, evaluated PCR data, and drafted the manuscript. AS performed the bacteriological examinations. JH and KA designed the PCR experiments/molecular analyses. JH and RH performed the statistical analysis. CE, AH, and MP edited the manuscript. All authors read and approved the final manuscript.

## Funding

The project was financed by the authors' institution. The authors were also supported by the German Research Foundation (DFG) and the University of Leipzig as part of the Open Access Publishing program.

## Conflict of Interest

The authors declare that the research was conducted in the absence of any commercial or financial relationships that could be construed as a potential conflict of interest.

## Publisher's Note

All claims expressed in this article are solely those of the authors and do not necessarily represent those of their affiliated organizations, or those of the publisher, the editors and the reviewers. Any product that may be evaluated in this article, or claim that may be made by its manufacturer, is not guaranteed or endorsed by the publisher.
