# Supplementary material for: Pathogen Screening for Possible Causes of Meningitis/Encephalitis in Wild Carnivores From Saxony-Anhalt
Source: Front Vet Sci. 2022 Apr 7;9:826355. doi: 10.3389/fvets.2022.826355 (PMC9021439; doi:10.3389/fvets.2022.826355)
Supplement: Supplementary file 1 [file Data_Sheet_1.PDF]

Supplementary Material

1 SUPPLEMENTARY DATA

Animals with Behavioral Data and Pathogens Detected

Out of the 1124 animals total, we had behavioral data for 136 individuals (12.10%). Abnormal behavior was noted in 121 individuals and normal in 15 others (Supplementary Table S1).

We could detect an infectious pathogen in 95 of the 121 animals (78.51%) with abnormal behavior. Significantly most often, canine distemper virus (CDV) alone (n=69) or in combination with other pathogens (n=19) was found (88/95; 92.63%; χ^2=7.84; p-value=0.0051). The pathogens combined with CDV were in detail: fox circovirus (FoxCV) (n=8), canine parvovirus type 2, 2a, 2b or 2c (CPV-2) (n=5), nematode larvae (n=2), *Toxoplasma gondii* (*T. gondii*) (n=1), FoxCV and *Streptococcus canis* (n=1), *Pasteurella canis* (n=1) as well as *Salmonella enterica* subsp. *enterica* (n=1). Among the other seven animals (7/95; 7.37%), FoxCV (n=6) and CPV-2 (n=1) were found. Histopathological changes were observed in 70 of the 95 animals (73.68%). It is known, that CDV-infected wild carnivores may show behavioral changes due to brain lesions (1, 2, 3, 4). This indicates a relationship between the distemper infection and the presence of the abnormal behavior, as demonstrated by Chi-squared test.

No pathogens were detected in the 26 remaining animals with abnormal behavior (26/121; 21.49%). However, histopathological changes were found in eight of them (30.77%).

Fifteen animals did show normal behavior (15/136; 11.03%). In seven of them (46.67%) an infectious pathogen was found. Again, CDV alone (n=3) or in combination with FoxCV (n=2) was most prevalent followed by FoxCV alone (n=1) and nematode larvae (n=1). Of the animals with normal behavior and pathogen detection, in five of seven (71.43%) histopathological changes were observed.

In eight of the 15 animals (53.33%) with normal behavior we failed to detect any pathogens with the tests we performed. One of them (12.50%) showed histopathological changes.

2 SUPPLEMENTARY FIGURES

	a,bp, fa	distribution			infiltrate composition				
		foc	mf	dif	ly	plc	mac	neu	eos
**cerebellum**									
meninx									
distr. infiltr.									
pva	sporadically:			partly:			dominating:		
diagnosis									
grey matter									
pva									
par									
sat									
gli									
nn									
np									
vac									
mal									
white matter									
pva									
par									
gli									
vac/dem									
mal									
diagnosis									
**cerebrum**									
meninx									
distr. infiltr.									
pva	sporadically:			partly:			dominating:		
diagnosis									
grey matter									
pva									
par									
sat									
gli									
nn									
np									
vac									
mal									
white matter									
pva									
par									
pve									
gli									
vac/dem									
mal									
diagnosis									

	a,bp, fa	distribution			infiltrate composition				
		foc	mf	dif	ly	plc	mac	neu	eos
**hippocampus**									
grey matter									
pva									
par									
sat									
gli									
nn									
np									
vac									
mal									
white matter									
pva									
par									
pve									
gli									
vac/dem									
mal									
diagnosis									
**brain stem/medulla oblongata**									
meninx									
distr. infiltr.									
pva	sporadically:			partly:			dominating:		
diagnosis									
grey matter									
pva									
par									
sat									
gli									
nn									
np									
vac									
mal									
white matter									
pva									
par									
gli									
vac/dem									
mal									
diagnosis									
ib	yes	no		loc.:					

main diagnosis:

lab-ID:

Figure S1. Evalutation scheme for histopathological examination of the brain samples. Abbreviations: autolysis (a), bacterial putrefaction (bp), freezing artifacts (fa), focal (foc), multifocal (mf), diffuse (dif), lymphocytes (ly), plasma cells (plc), macrophages (mac), neutrophil granulocytes (neu), eosinophilic granulocytes (eos), distribution of infiltrates (distr. infiltr.), perivascular (pva), parenchymatous (par), satellitosis (sat), gliosis (gli), neuronal necrosis (nn), neuronophagia (np), vacuolization (vac), malacia (mal), vacuolization/demyelination (vac/dem), periventricular (pve), inclusion bodies (ib), localization (loc)

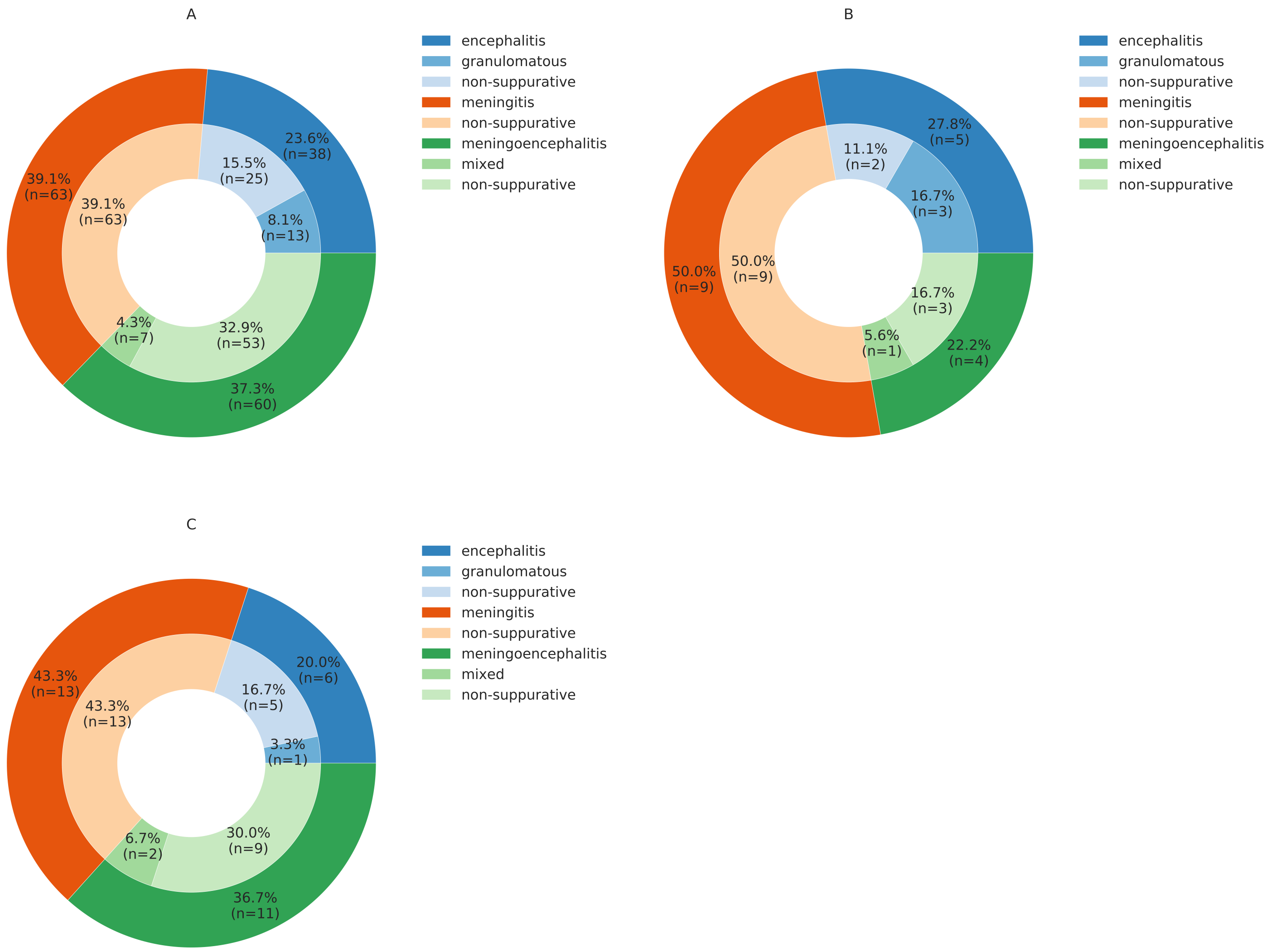

Figure S2. Localization (outer circle) and associated characterization (inner circle) of inflammatory processes in the brain of animals with detection of **(A)** CDV (*n*=161/349), **(B)** CPV-2 (*n*=18/72) and **(C)** FoxCV (*n*=30/77); Abbreviation: number of animals (*n*)

3 SUPPLEMENTARY TABLES

Table S1 Data of all animals in the study, including Lab-ID, signalment, preliminary report, main histopathological findings in the brain and pathogens detected with method. Abbreviations: Landkreis Anhalt-Bitterfeld (ABI), Landkreis Boerde (BK), Burgenlandkreis (BLK), Dessau-Rosslau (DE), Halle (Saale) (HAL), Landkreis Harz (HZ), Landkreis Jerichower-Land (JL), Magdeburg (MD), Landkreis Mansfeld-Suedharz (MSH), Altmarkkreis Salzwedel (SAW), Landkreis Stendal (SDL), Landkreis Saalekreis (SK), Salzlandkreis (SLK), Landkreis Wittenberg (WB), canine distemper virus (CDV), canine parvovirus type 2, 2a, 2b or 2c (CPV-2), fox circovirus (FoxCV), *Listeria monocytogenes* (*L. monocytogenes*), *Neospora caninum* (*N. caninum*), *Toxoplasma gondii* (*T. gondii*), (reverse transcription) quantitative polymerase chain reaction ((RT-)qPCR); bacteriological examination (BE); histopathological examination (HE); immunohistochemistry (IHC)

Lab-ID	Date of death or discovery	Species	Gender	Age	Administrative District	Cause of death	Behavior	Histopathological findings in the brain	Pathogens detected (method)
16410002955	2016-01-04	red fox	male	adult	SK	shot	abnormal	non-suppurative meningitis	none
16410002956	2016-01-06	raccoon	female	adult	JL	found dead	not specified	no significant findings	none
16410004506	2016-01-06	red fox	male	adult	ABI	found dead	not specified	no significant findings	none
16410007250	2016-01-08	red fox	male	adult	MSH	shot	not specified	non-suppurative meningitis, gliosis, vacuolization/demyelination	none
16410007251	2016-01-10	raccoon	female	adult	BK	shot	not specified	no significant findings	none
16410007252	2016-01-10	marten	male	adult	BK	shot	not specified	non-suppurative encephalitis, gliosis	none
16410007263	2016-01-11	red fox	male	adult	SAW	shot	not specified	non-suppurative encephalitis, gliosis	none
16410007276	2016-01-11	red fox	male	adult	SAW	shot	not specified	no significant findings	none
16410009150	2016-01-09	red fox	male	adult	HZ	shot	not specified	vacuolization/demyelination	none
16410009151	2016-01-07	red fox	male	adult	SK	shot	not specified	no significant findings	none
16410009254	2016-01-09	red fox	male	adult	HZ	shot	not specified	no significant findings	none
16410010780	2016-01-09	red fox	male	adult	HZ	shot	not specified	no significant findings	none
16410010781	2016-01-10	red fox	male	adult	HZ	shot	not specified	no significant findings	none
16410010782	2016-01-10	marten	male	adult	ABI	shot	not specified	no significant findings	none
16410013021	2016-01-13	red fox	male	adult	HZ	shot	abnormal	no significant findings	none
16410014321	2016-01-15	red fox	male	adult	HAL	shot	not specified	no significant findings	*Streptococcus canis* (BE)

Continued on the following page

Lab-ID	Date of death or discovery	Species	Gender	Age	Administrative District	Cause of death	Behavior	Histopathological findings in the brain	Pathogens detected (method)
16410016388	2016-01-16	red fox	female	adult	SAW	shot	not specified	no significant findings	none
16410016389	2016-01-16	raccoon	male	adult	SAW	shot	not specified	no significant findings	CPV-2 ((RT-)qPCR)
16410016390	2016-01-16	red fox	female	adult	SAW	shot	not specified	no significant findings	none
16410016391	2016-01-16	red fox	female	adult	SAW	shot	not specified	no significant findings	none
16410016392	2016-01-16	red fox	female	adult	DE	shot	not specified	non-suppurative meningitis	CDV ((RT-)qPCR)
16410016393	2016-01-17	red fox	male	adult	MSH	shot	not specified	no significant findings	none
16410016398	2016-01-16	red fox	male	adult	MSH	shot	not specified	no significant findings	none
16410017649	2016-01-17	red fox	male	adult	BLK	shot	not specified	no significant findings	none
16410017650	2016-01-19	red fox	male	adult	ABI	shot	not specified	no significant findings	FoxCV ((RT-)qPCR)
16410018322	2016-01-19	red fox	male	adult	SDL	shot	not specified	gliosis, satellitosis, vacuolization/demyelination	CDV ((RT-)qPCR)
16410022109	2016-01-21	red fox	male	adult	HZ	shot	not specified	non-suppurative meningitis, gliosis, satellitosis, neuronal necrosis	none
16410022110	2016-01-16	red fox	male	adult	HZ	shot	not specified	no significant findings	none
16410022472	2016-01-21	raccoon	male	adult	ABI	shot	not specified	non-suppurative encephalitis	none
16410023908	2016-01-21	red fox	male	adult	HAL	not specified	not specified	non-suppurative meningoencephalitis, gliosis	CDV ((RT-)qPCR), *Streptococcus canis* (BE)
16410023909	2016-01-21	red fox	male	juvenile	SAW	shot	not specified	gliosis	none
16410023910	2016-01-19	red fox	male	adult	MSH	shot	not specified	non-suppurative meningitis	none
16410024085	2016-01-23	red fox	male	adult	SDL	shot	not specified	non-suppurative meningitis	none
16410024086	2016-01-23	red fox	male	adult	SDL	shot	not specified	no significant findings	none
16410024087	2016-01-23	red fox	female	adult	SDL	shot	not specified	no significant findings	none
16410024088	2016-01-23	red fox	male	adult	SDL	shot	not specified	no significant findings	none
16410024091	2016-01-23	red fox	male	adult	SDL	shot	not specified	no significant findings	none
16410024092	2016-01-23	red fox	female	adult	SDL	shot	not specified	no significant findings	none
16410024093	2016-01-23	red fox	male	adult	SDL	shot	not specified	no significant findings	none
16410025131	2016-01-21	red fox	male	adult	ABI	shot	not specified	no significant findings	none

Continued on the following page

Lab-ID	Date of death or discovery	Species	Gender	Age	Administrative District	Cause of death	Behavior	Histopathological findings in the brain	Pathogens detected (method)
16410025132	2016-01-24	red fox	male	adult	JL	shot	not specified	non-suppurative meningitis	none
16410025133	2016-01-21	red fox	male	adult	MSH	shot	not specified	no significant findings	none
16410025138	2016-01-21	red fox	male	adult	MSH	shot	not specified	no significant findings	none
16410025139	2016-01-21	red fox	male	adult	MSH	shot	not specified	no significant findings	none
16410025144	2016-01-21	red fox	female	adult	ABI	shot	not specified	non-suppurative meningoencephalitis, gliosis, satellitosis, neuronophagia, neuronal necrosis, vacuolization/demyelination	none
16410025145	2016-01-21	red fox	female	adult	ABI	shot	not specified	no significant findings	none
16410025146	2016-01-21	red fox	male	adult	ABI	shot	not specified	no significant findings	none
16410025147	2016-01-21	red fox	male	adult	ABI	shot	not specified	no significant findings	none
16410025148	2016-01-21	red fox	female	adult	ABI	shot	not specified	non-suppurative meningitis	none
16410027120	2016-01-23	red fox	male	adult	HZ	shot	not specified	gliosis, vacuolization/demyelination	none
16410027121	2016-01-23	red fox	female	adult	HZ	shot	not specified	no significant findings	none
16410027123	2016-01-23	red fox	male	adult	BK	shot	not specified	non-suppurative meningitis	none
16410027124	2016-01-25	red fox	male	adult	MSH	shot	not specified	no significant findings	none
16410027125	2016-01-24	red fox	male	adult	MSH	shot	not specified	no significant findings	CDV ((RT-)qPCR)
16410027126	2016-01-25	red fox	female	adult	SAW	shot	not specified	non-suppurative meningoencephalitis, gliosis	none
16410027127	2016-01-26	red fox	male	adult	SAW	shot	not specified	gliosis, vacuolization/demyelination	none
16410027128	2016-01-22	red fox	male	adult	SAW	shot	not specified	no significant findings	FoxCV ((RT-)qPCR)
16410027129	2016-01-26	red fox	male	adult	SAW	shot	not specified	no significant findings	none
16410027130	2016-01-23	red fox	female	adult	HZ	shot	not specified	non-suppurative meningitis	none
16410027227	2016-01-23	red fox	female	adult	BK	shot	not specified	no significant findings	CDV ((RT-)qPCR)
16410027229	2016-01-23	red fox	male	adult	BK	shot	not specified	no significant findings	none
16410027362	2016-01-26	red fox	female	adult	SAW	shot	not specified	no significant findings	none
16410027363	2016-01-26	red fox	female	adult	SAW	shot	not specified	no significant findings	none

Continued on the following page

Lab-ID	Date of death or discovery	Species	Gender	Age	Administrative District	Cause of death	Behavior	Histopathological findings in the brain	Pathogens detected (method)
16410030211	2016-01-23	red fox	male	adult	HZ	shot	not specified	non-suppurative meningoencephalitis	none
16410030212	2016-01-23	red fox	male	adult	HZ	shot	not specified	no significant findings	none
16410030213	2016-01-23	red fox	male	adult	HZ	shot	not specified	non-suppurative meningitis	none
16410030214	2016-01-26	red fox	female	adult	HZ	shot	not specified	no significant findings	none
16410032413	2016-01-23	red fox	female	adult	HZ	shot	not specified	no significant findings	none
16410032414	2016-01-23	red fox	male	adult	HZ	shot	not specified	non-suppurative meningoencephalitis, gliosis	none
16410033948	2016-01-28	red fox	male	adult	SDL	found dead	normal	non-suppurative meningitis	CDV ((RT-)qPCR)
16410038994	2016-01-30	red fox	female	adult	HZ	shot	not specified	no significant findings	none
16410038995	2016-02-01	red fox	male	adult	BLK	shot	abnormal	vacuolization/demyelination	CDV ((RT-)qPCR)
16410041158	2016-02-01	red fox	male	adult	MSH	shot	not specified	no significant findings	none
16410041202	2016-01-31	red fox	male	adult	HZ	shot	not specified	no significant findings	none
16410042635	2016-01-30	red fox	male	adult	MSH	shot	not specified	no significant findings	none
16410046261	2016-02-05	red fox	female	adult	HAL	not specified	not specified	non-suppurative encephalitis, vacuolization/demyelination	CDV ((RT-)qPCR)
16410046765	2016-02-07	red fox	male	adult	BK	shot	not specified	no significant findings	none
16410046766	2016-02-06	red fox	female	adult	BK	shot	not specified	vacuolization/demyelination	CDV ((RT-)qPCR)
16410046767	2016-02-06	red fox	male	adult	BK	shot	not specified	vacuolization/demyelination	FoxCV ((RT-)qPCR)
16410046768	2016-02-06	raccoon	female	adult	MSH	shot	not specified	no significant findings	CPV-2 ((RT-)qPCR)
16410048006	2016-02-06	red fox	male	adult	BK	shot	not specified	vacuolization/demyelination	none
16410048007	2016-02-07	raccoon	female	adult	SAW	shot	not specified	no significant findings	none
16410049103	2016-02-09	red fox	male	adult	SK	found dead	not specified	vacuolization/demyelination	none
16410050819	2016-02-09	raccoon	female	adult	HZ	shot	not specified	no significant findings	none
16410050820	2016-02-11	red fox	female	adult	ABI	found dead	not specified	no significant findings	none

Continued on the following page

Lab-ID	Date of death or discovery	Species	Gender	Age	Administrative District	Cause of death	Behavior	Histopathological findings in the brain	Pathogens detected (method)
16410050821	2016-02-11	red fox	male	adult	ABI	not specified	not specified	vacuolization/demyelination	none
16410054917	2016-02-11	red fox	male	adult	HAL	not specified	not specified	no significant findings	none
16410057359	2016-02-13	red fox	male	adult	HAL	shot	abnormal	gliosis, vacuolization/demyelination	none
16410057361	2016-02-15	red fox	female	adult	HAL	shot	abnormal	non-suppurative meningoencephalitis, gliosis, satellitosis, neuronophagia, neuronal necrosis, vacuolization/demyelination	CDV ((RT-)qPCR)
16410057362	2016-02-15	raccoon	male	adult	HAL	shot	not specified	non-suppurative meningitis, gliosis	CDV ((RT-)qPCR)
16410057363	2016-02-14	badger	female	adult	BLK	shot	abnormal	non-suppurative meningoencephalitis, gliosis, satellitosis	CPV-2 ((RT-)qPCR)
16410058457	2016-02-14	red fox	male	adult	SK	shot	abnormal	gliosis, satellitosis, neuronophagia, neuronal necrosis	CDV ((RT-)qPCR)
16410060140	2016-02-14	red fox	female	adult	SAW	not specified	not specified	non-suppurative meningoencephalitis, vacuolization/demyelination	none
16410060141	2016-02-16	red fox	female	adult	SAW	shot	not specified	no significant findings	none
16410060153	2016-02-16	red fox	male	adult	SAW	shot	not specified	no significant findings	none
16410060154	2016-02-16	red fox	male	adult	SAW	shot	not specified	no significant findings	none
16410060155	2016-02-16	red fox	female	adult	SAW	shot	not specified	no significant findings	none
16410069477	2016-02-22	raccoon	male	adult	ABI	shot	not specified	non-suppurative meningitis, gliosis, vacuolization/demyelination	none
16410069478	2016-02-22	red fox	male	adult	SAW	shot	not specified	gliosis, vacuolization/demyelination	none
16410069578	2016-02-22	red fox	female	adult	SAW	shot	not specified	vacuolization/demyelination	none
16410069579	2016-02-22	red fox	male	adult	SAW	shot	not specified	gliosis, vacuolization/demyelination	none
16410071044	2016-02-21	red fox	female	adult	BK	shot	not specified	no significant findings	CDV ((RT-)qPCR)
16410071045	2016-02-23	red fox	male	adult	HAL	not specified	not specified	no significant findings	none
16410073208	2016-02-23	red fox	female	adult	BK	shot	not specified	no significant findings	none

Continued on the following page

Lab-ID	Date of death or discovery	Species	Gender	Age	Administrative District	Cause of death	Behavior	Histopathological findings in the brain	Pathogens detected (method)
16410073209	2016-02-19	raccoon	female	adult	SK	shot	not specified	no significant findings	CDV/CPV-2 ((RT-)qPCR)
16410075468	2016-02-27	red fox	female	adult	SDL	shot	not specified	no significant findings	CDV ((RT-)qPCR)
16410075565	2016-02-23	red fox	male	adult	BLK	shot	not specified	no significant findings	CDV ((RT-)qPCR)
16410075566	2016-02-25	red fox	male	adult	HZ	shot	not specified	no significant findings	CDV/FoxCV ((RT-)qPCR)
16410077630	2016-02-28	red fox	female	adult	SK	shot	abnormal	non-suppurative encephalitis, gliosis, vacuolization/demyelination	CDV ((RT-)qPCR)
16410079273	2016-02-28	red fox	male	adult	BLK	shot	not specified	no significant findings	CDV ((RT-)qPCR)
16410079277	2016-02-28	red fox	female	adult	SK	found dead	abnormal	no significant findings	CDV ((RT-)qPCR)
16410079283	2016-03-01	red fox	male	adult	SK	found dead	not specified	non-suppurative encephalitis, gliosis, satellitosis	CDV ((RT-)qPCR)
16410081900	2016-03-01	red fox	male	adult	HAL	not specified	not specified	gliosis, vacuolization/demyelination	none
16410087112	2016-03-05	red fox	male	adult	WB	shot	abnormal	non-suppurative meningitis, gliosis, satellitosis, neuronophagia, neuronal necrosis, vacuolization/demyelination	CDV/FoxCV ((RT-)qPCR)
16410087113	2016-03-04	red fox	female	adult	ABI	found dead	normal	non-suppurative meningitis, gliosis, vacuolization/demyelination	CDV/FoxCV ((RT-)qPCR)
16410089589	2016-03-06	red fox	female	adult	SAW	shot	not specified	no significant findings	none
16410089590	2016-03-07	raccoon	female	adult	SAW	shot	not specified	no significant findings	none
16410093955	2016-03-10	red fox	male	adult	SK	shot	not specified	non-suppurative meningitis	none
16410095926	2016-03-10	red fox	male	adult	DE	found dead	abnormal	no significant findings	none
16410096032	2016-03-10	red fox	male	adult	SK	shot	not specified	non-suppurative meningoencephalitis, vacuolization/demyelination	CDV ((RT-)qPCR)
16410096033	2016-03-10	red fox	male	adult	WB	shot	abnormal	vacuolization/demyelination	CDV ((RT-)qPCR)
16410096034	2016-03-10	red fox	male	adult	WB	shot	abnormal	vacuolization/demyelination	CDV ((RT-)qPCR)
16410097450	2016-03-11	raccoon	male	adult	SAW	shot	not specified	non-suppurative encephalitis, gliosis, vacuolization/demyelination	CDV ((RT-)qPCR)

Continued on the following page

Lab-ID	Date of death or discovery	Species	Gender	Age	Administrative District	Cause of death	Behavior	Histopathological findings in the brain	Pathogens detected (method)
16410099588	2016-03-14	red fox	female	adult	SK	shot	not specified	no significant findings	none
16410103436	2016-03-15	raccoon	male	adult	ABI	shot	not specified	no significant findings	CPV-2 ((RT-)qPCR)
16410103437	2016-03-13	raccoon dog	female	adult	JL	shot	not specified	mixed meningoencephalitis, vacuolization/demyelination, malacia	CDV ((RT-)qPCR)
16410108666	2016-02-26	red fox	female	adult	SAW	shot	not specified	no significant findings	none
16410110704	2016-03-21	raccoon	female	adult	SAW	shot	not specified	no significant findings	none
16410110705	2016-03-21	raccoon dog	male	adult	SK	shot	not specified	non-suppurative meningitis	CDV ((RT-)qPCR), beta-hemolytic *Escherichia coli* (BE)
16410110710	2016-03-21	red fox	female	adult	SK	shot	not specified	no significant findings	CDV/FoxCV ((RT-)qPCR)
16410110711	2016-03-19	red fox	female	adult	SK	shot	not specified	gliosis, satellitosis	CDV ((RT-)qPCR)
16410110712	2016-03-19	red fox	male	adult	SK	shot	not specified	non-suppurative meningoencephalitis, malacia	CDV ((RT-)qPCR)
16410110816	2016-03-21	red fox	female	adult	SK	shot	not specified	no significant findings	CDV ((RT-)qPCR)
16410113128	2016-03-20	raccoon dog	male	adult	ABI	shot	not specified	non-suppurative meningitis, gliosis	CDV ((RT-)qPCR)
16410116103	2016-03-22	red fox	male	adult	SK	shot	abnormal	non-suppurative encephalitis, gliosis, vacuolization/demyelination	CDV ((RT-)qPCR)
16410116104	2016-03-22	red fox	male	adult	ABI	shot	not specified	no significant findings	CDV ((RT-)qPCR)
16410118066	2016-03-24	raccoon	male	adult	SAW	shot	not specified	gliosis, satellitosis	none
16410121101	2016-03-28	raccoon	female	adult	SLK	shot	abnormal	vacuolization/demyelination	CDV ((RT-)qPCR)
16410121102	2016-03-29	raccoon	male	adult	SAW	shot	not specified	no significant findings	CDV ((RT-)qPCR)
16410129116	2016-03-19	red fox	female	adult	SK	not specified	not specified	non-suppurative encephalitis	none
16410133021	2016-04-04	raccoon	male	adult	MSH	shot	not specified	non-suppurative meningoencephalitis	none
16410133022	2016-03-31	red fox	female	adult	SAW	found dead	not specified	no significant findings	none
16410136743	2016-04-03	red fox	female	adult	BLK	found dead	normal	non-suppurative meningoencephalitis, gliosis	CDV ((RT-)qPCR)

Continued on the following page

Lab-ID	Date of death or discovery	Species	Gender	Age	Administrative District	Cause of death	Behavior	Histopathological findings in the brain	Pathogens detected (method)
16410139251	2016-04-06	red fox	male	adult	BLK	found dead	not specified	gliosis, vacuolization/demyelination	none
16410141501	2016-04-08	red fox	male	adult	WB	shot	not specified	no significant findings	none
16410141502	2016-04-06	red fox	female	adult	ABI	shot	not specified	no significant findings	none
16410146162	2016-04-11	red fox	female	adult	SK	shot	not specified	non-suppurative meningoencephalitis, gliosis	CDV ((RT-)qPCR)
16410146163	2016-04-10	marten	female	adult	ABI	shot	not specified	no significant findings	none
16410149406	2016-04-13	red fox	female	adult	SAW	shot	not specified	no significant findings	none
16410151256	2016-04-12	red fox	female	adult	HZ	shot	not specified	no significant findings	none
16410151257	2016-04-13	raccoon	female	adult	SK	shot	not specified	no significant findings	none
16410153493	2016-03-16	red fox	female	adult	ABI	found dead	not specified	no significant findings	none
16410155472	2016-04-17	red fox	female	adult	SK	shot	not specified	no significant findings	CDV ((RT-)qPCR), *Salmonella enterica* subsp. *enterica* (BE)
16410160068	2016-04-19	raccoon	male	adult	ABI	shot	not specified	no significant findings	none
16410162856	2016-04-20	red fox	female	adult	SAW	found dead	not specified	no significant findings	none
16410162978	2016-04-21	raccoon	male	adult	SAW	shot	not specified	no significant findings	none
16410163018	2016-04-16	raccoon	male	adult	SAW	shot	not specified	no significant findings	none
16410163039	2016-04-20	raccoon	male	adult	SAW	shot	not specified	no significant findings	none
16410164079	2016-04-22	red fox	male	adult	MSH	shot	abnormal	no significant findings	none
16410169905	2016-04-26	red fox	male	adult	HAL	not specified	not specified	non-suppurative meningoencephalitis, gliosis, satellitosis, vacuolization/demyelination	CDV ((RT-)qPCR)
16410169906	2016-04-25	red fox	male	adult	SK	shot	not specified	no significant findings	none
16410169907	2016-04-26	red fox	female	adult	HZ	shot	not specified	no significant findings	none
16410181246	2016-04-26	red fox	female	adult	SAW	shot	not specified	no significant findings	none
16410181247	2016-05-01	red fox	male	adult	BLK	shot	not specified	no significant findings	none

Continued on the following page

Lab-ID	Date of death or discovery	Species	Gender	Age	Administrative District	Cause of death	Behavior	Histopathological findings in the brain	Pathogens detected (method)
16410183668	2016-05-02	red fox	male	adult	BLK	found dead	not specified	no significant findings	nonhemolytic *Escherichia coli* (BE)
16410189438	2016-05-06	red fox	male	adult	BLK	shot	not specified	non-suppurative meningoencephalitis, gliosis	CDV ((RT-)qPCR)
16410191423	2016-05-11	red fox	female	adult	SDL	shot	not specified	non-suppurative encephalitis, vacuolization/demyelination	CDV/FoxCV ((RT-)qPCR)
16410192663	2016-05-10	red fox	male	adult	HZ	shot	not specified	no significant findings	none
16410192664	2016-05-11	red fox	male	adult	MD	shot	abnormal	no significant findings	CDV ((RT-)qPCR)
16410194786	2016-05-10	red fox	female	adult	BLK	shot	not specified	no significant findings	none
16410196502	2016-05-14	red fox	male	juvenile	BLK	shot	not specified	no significant findings	none
16410197079	2016-05-15	red fox	female	adult	SDL	shot	not specified	no significant findings	none
16410197251	2016-05-17	marten	male	adult	SDL	found dead	normal	no significant findings	none
16410201711	2016-05-19	red fox	male	adult	ABI	shot	abnormal	gliosis, vacuolization/demyelination	CDV ((RT-)qPCR)
16410202355	2016-05-19	marten	male	adult	BLK	shot	not specified	no significant findings	none
16410205493	2016-05-22	red fox	male	adult	SK	shot	not specified	non-suppurative meningoencephalitis	CDV ((RT-)qPCR)
16410205494	2016-05-21	red fox	female	adult	MSH	shot	not specified	no significant findings	none
16410207453	2016-05-23	red fox	male	juvenile	MD	shot	not specified	no significant findings	CDV ((RT-)qPCR)
16410207454	2016-05-21	red fox	male	adult	SAW	shot	not specified	no significant findings	none
16410214419	2016-05-30	raccoon	male	adult	BLK	shot	not specified	no significant findings	none
16410214439	2016-05-31	red fox	male	adult	MSH	shot	abnormal	mixed meningoencephalitis, satellitosis	none
16410214440	2016-05-28	red fox	male	adult	ABI	shot	abnormal	non-suppurative meningoencephalitis, gliosis, vacuolization/demyelination	CDV ((RT-)qPCR)
16410215137	2016-06-01	marten	male	juvenile	SDL	shot	not specified	no significant findings	none
16410215893	2016-05-29	raccoon	male	adult	SAW	shot	not specified	non-suppurative encephalitis, gliosis, satellitosis	none
16410215894	2016-06-01	red fox	female	juvenile	DE	found dead	not specified	no significant findings	none

Continued on the following page

Lab-ID	Date of death or discovery	Species	Gender	Age	Administrative District	Cause of death	Behavior	Histopathological findings in the brain	Pathogens detected (method)
16410216953	2016-06-02	red fox	male	juvenile	SAW	found dead	not specified	no significant findings	none
16410218308	2016-06-02	red fox	male	juvenile	BLK	shot	not specified	no significant findings	none
16410220097	2016-06-03	red fox	female	juvenile	SK	shot	not specified	gliosis	none
16410223029	2016-06-07	red fox	male	adult	BLK	shot	not specified	no significant findings	nonhemolytic *Escherichia coli* (BE)
16410225304	2016-06-10	red fox	female	adult	BK	shot	not specified	no significant findings	none
16410226947	2016-06-13	red fox	female	adult	DE	shot	not specified	non-suppurative meningitis	none
16410229536	2016-06-14	red fox	male	adult	SK	shot	not specified	no significant findings	CDV ((RT-)qPCR)
16410229537	2016-06-13	raccoon dog	male	adult	MSH	shot	not specified	non-suppurative encephalitis	none
16410231435	2016-06-15	raccoon	male	adult	HZ	shot	not specified	no significant findings	CPV-2 ((RT-)qPCR)
16410232284	2016-06-17	red fox	male	adult	MSH	shot	not specified	no significant findings	none
16410234950	2016-06-16	raccoon	male	adult	ABI	shot	not specified	no significant findings	none
16410238243	2016-06-19	red fox	male	juvenile	BLK	shot	not specified	no significant findings	none
16410238244	2016-06-21	red fox	female	juvenile	BLK	shot	not specified	no significant findings	none
16410240113	2016-06-27	red fox	female	adult	BLK	shot	not specified	non-suppurative encephalitis, gliosis, satellitosis	CDV ((RT-)qPCR)
16410240114	2016-06-28	red fox	male	juvenile	SK	shot	not specified	non-suppurative meningoencephalitis, gliosis, vacuolization/demyelination	CDV ((RT-)qPCR)
16410240115	2016-06-26	red fox	male	adult	SAW	shot	not specified	no significant findings	none
16410243716	2016-06-30	red fox	male	adult	MSH	shot	not specified	vacuolization/demyelination	none
16410243720	2016-06-30	red fox	male	adult	MSH	shot	not specified	no significant findings	none
16410244731	2016-07-03	red fox	female	juvenile	SAW	shot	not specified	gliosis, satellitosis, neuronophagia, neuronal necrosis, vacuolization/demyelination	FoxCV ((RT-)qPCR)
16410244732	2016-07-04	red fox	female	juvenile	DE	shot	not specified	non-suppurative meningitis, gliosis, satellitosis	FoxCV ((RT-)qPCR)
16410245858	2016-07-04	red fox	female	juvenile	SK	shot	not specified	non-suppurative meningoencephalitis, gliosis	CDV ((RT-)qPCR)

Continued on the following page

Lab-ID	Date of death or discovery	Species	Gender	Age	Administrative District	Cause of death	Behavior	Histopathological findings in the brain	Pathogens detected (method)
16410247565	2016-07-04	red fox	male	juvenile	SAW	shot	not specified	no significant findings	none
16410247566	2016-07-05	red fox	male	juvenile	DE	found dead	not specified	non-suppurative meningoencephalitis, vacuolization/demyelination	none
16410248815	2016-07-05	red fox	female	juvenile	BLK	shot	not specified	non-suppurative meningoencephalitis	none
16410249837	2016-07-11	red fox	female	juvenile	HZ	shot	not specified	no significant findings	none
16410249838	2016-07-11	red fox	male	adult	HZ	shot	not specified	non-suppurative meningoencephalitis, gliosis	none
16410249839	2016-07-10	red fox	male	adult	SLK	shot	abnormal	non-suppurative meningitis	CDV ((RT-)qPCR)
16410251304	2016-07-10	raccoon	female	adult	SAW	shot	not specified	no significant findings	none
16410252370	2016-07-12	red fox	male	adult	BK	shot	abnormal	non-suppurative meningoencephalitis	CDV/FoxCV ((RT-)qPCR)
16410253013	2016-07-09	raccoon	male	juvenile	SAW	shot	not specified	no significant findings	none
16410253022	2016-07-05	raccoon	male	juvenile	SAW	shot	not specified	no significant findings	none
16410253023	2016-07-12	raccoon	male	adult	SK	shot	not specified	no significant findings	none
16410255214	2016-07-18	raccoon	male	adult	SDL	found dead	not specified	gliosis	CDV ((RT-)qPCR)
16410256692	2016-07-02	red fox	female	adult	SAW	shot	not specified	no significant findings	none
16410256693	2016-07-18	red fox	male	adult	SK	shot	not specified	no significant findings	CDV ((RT-)qPCR)
16410256694	2016-07-15	raccoon	male	juvenile	SAW	shot	not specified	no significant findings	none
16410256697	2016-07-15	raccoon	male	juvenile	SAW	shot	not specified	no significant findings	none
16410256698	2016-07-15	raccoon	female	juvenile	SAW	shot	not specified	no significant findings	none
16410257695	2016-07-18	red fox	male	adult	HZ	shot	not specified	non-suppurative encephalitis, vacuolization/demyelination	none
16410257697	2016-07-18	red fox	female	adult	HZ	shot	not specified	no significant findings	none
16410257699	2016-07-18	red fox	male	adult	WB	shot	abnormal	gliosis, vacuolization/demyelination	CDV/FoxCV ((RT-)qPCR)
16410257701	2016-07-18	raccoon	male	adult	SAW	shot	not specified	no significant findings	none
16410257702	2016-07-18	red fox	female	adult	SLK	shot	not specified	no significant findings	none
16410259062	2016-07-19	raccoon	female	adult	SAW	shot	not specified	no significant findings	none

Continued on the following page

Lab-ID	Date of death or discovery	Species	Gender	Age	Administrative District	Cause of death	Behavior	Histopathological findings in the brain	Pathogens detected (method)
16410260400	2016-07-20	red fox	male	adult	HZ	shot	not specified	no significant findings	none
16410262816	2016-07-22	raccoon	male	adult	HZ	shot	not specified	no significant findings	none
16410262817	2016-07-25	red fox	male	adult	MSH	shot	not specified	non-suppurative meningoencephalitis, vacuolization/demyelination	none
16410264542	2016-07-25	red fox	male	adult	HZ	shot	not specified	no significant findings	none
16410264543	2016-07-24	red fox	female	adult	SK	shot	not specified	no significant findings	none
16410266316	2016-07-25	red fox	female	adult	BLK	shot	not specified	no significant findings	none
16410266317	2016-07-26	raccoon	female	juvenile	SAW	shot	not specified	no significant findings	none
16410266318	2016-07-15	raccoon	female	juvenile	SAW	shot	not specified	no significant findings	none
16410266427	2016-07-26	raccoon	female	juvenile	SAW	shot	not specified	no significant findings	none
16410267522	2016-07-24	red fox	male	adult	BLK	shot	normal	no significant findings	none
16410269753	2016-07-28	red fox	male	juvenile	HZ	shot	not specified	no significant findings	none
16410270299	2016-07-30	red fox	female	adult	JL	shot	not specified	non-suppurative encephalitis, vacuolization/demyelination	none
16410270300	2016-07-31	red fox	male	adult	BLK	shot	not specified	granulomatous encephalitis, vacuolization/demyelination	larvae of nematodes (HE)
16410271549	2016-08-02	red fox	female	adult	SAW	shot	not specified	no significant findings	none
16410272765	2016-08-03	red fox	female	juvenile	SK	shot	not specified	non-suppurative meningitis	FoxCV ((RT-)qPCR)
16410272766	2016-08-02	raccoon dog	male	juvenile	HZ	shot	not specified	non-suppurative meningitis	none
16410273588	2016-08-02	red fox	female	adult	HZ	shot	normal	granulomatous meningitis	larvae of nematodes (HE)
16410275021	2016-08-04	red fox	male	adult	MSH	shot	not specified	no significant findings	none
16410275031	2016-08-08	red fox	male	juvenile	SDL	shot	abnormal	no significant findings	none
16410275639	2016-08-06	raccoon	female	juvenile	SAW	shot	not specified	gliosis, vacuolization/demyelination	none
16410275640	2016-08-06	raccoon	male	adult	HZ	shot	not specified	non-suppurative encephalitis, gliosis, satellitosis, vacuolization/demyelination	none
16410275641	2016-08-07	red fox	male	adult	SK	shot	not specified	granulomatous encephalitis	larvae of nematodes (HE)
16410275642	2016-08-08	red fox	male	juvenile	HZ	shot	not specified	no significant findings	none

Continued on the following page

Lab-ID	Date of death or discovery	Species	Gender	Age	Administrative District	Cause of death	Behavior	Histopathological findings in the brain	Pathogens detected (method)
16410275643	2016-08-06	red fox	female	juvenile	HZ	shot	not specified	no significant findings	none
16410277068	2016-08-06	red fox	male	adult	SLK	shot	normal	no significant findings	none
16410277069	2016-08-07	red fox	male	adult	BLK	shot	not specified	no significant findings	none
16410278794	2016-08-09	red fox	female	adult	WB	shot	abnormal	gliosis, satellitosis, neuronophagia, neuronal necrosis	CDV/FoxCV ((RT-)qPCR), *Streptococcus canis* (BE)
16410279392	2016-07-27	raccoon	female	juvenile	SAW	shot	not specified	no significant findings	none
16410279393	2016-08-11	raccoon	male	juvenile	SK	shot	not specified	gliosis, satellitosis	none
16410279463	2016-07-27	raccoon	female	juvenile	SAW	shot	not specified	no significant findings	none
16410280454	2016-08-10	red fox	female	adult	HZ	shot	not specified	non-suppurative meningitis, gliosis, vacuolization/demyelination	none
16410280455	2016-08-12	red fox	male	adult	DE	found dead	not specified	no significant findings	none
16410282081	2016-08-15	red fox	male	juvenile	HZ	shot	not specified	no significant findings	none
16410282082	2016-08-16	red fox	female	juvenile	SK	shot	not specified	no significant findings	CPV-2 ((RT-)qPCR)
16410282083	2016-08-14	red fox	female	adult	SAW	shot	not specified	no significant findings	none
16410282084	2016-08-11	raccoon	male	adult	BLK	shot	not specified	no significant findings	none
16410283790	2016-08-15	red fox	male	adult	MSH	shot	not specified	no significant findings	none
16410283791	2016-08-16	red fox	female	adult	HZ	shot	not specified	no significant findings	none
16410283792	2016-08-17	red fox	male	adult	BK	not specified	not specified	no significant findings	none
16410284513	2016-08-17	red fox	male	adult	HZ	shot	normal	no significant findings	none
16410284514	2016-08-17	red fox	male	adult	SK	shot	not specified	mixed meningoencephalitis, gliosis	none
16410284515	2016-08-17	red fox	male	adult	WB	found dead	not specified	no significant findings	CDV/FoxCV ((RT-)qPCR)
16410290714	2016-08-22	red fox	female	juvenile	BLK	shot	not specified	no significant findings	CDV ((RT-)qPCR)
16410290715	2016-08-21	red fox	male	adult	SAW	shot	not specified	no significant findings	none
16410292005	2016-08-18	red fox	female	adult	HZ	shot	not specified	no significant findings	none
16410294463	2016-08-22	red fox	male	adult	SAW	shot	not specified	no significant findings	none

Continued on the following page

Lab-ID	Date of death or discovery	Species	Gender	Age	Administrative District	Cause of death	Behavior	Histopathological findings in the brain	Pathogens detected (method)
16410296816	2016-08-24	raccoon	female	adult	SAW	shot	not specified	mixed encephalitis, gliosis	none
16410296817	2016-08-23	red fox	female	adult	SK	shot	not specified	no significant findings	none
16410297185	2016-08-26	raccoon dog	male	adult	SAW	shot	not specified	granulomatous encephalitis	larvae of nematodes (HE)
16410298554	2016-08-28	red fox	male	adult	MSH	shot	not specified	no significant findings	none
16410299421	2016-08-28	raccoon dog	male	juvenile	SAW	shot	not specified	no significant findings	none
16410302999	2016-08-31	badger	male	adult	BLK	shot	not specified	no significant findings	none
16410303000	2016-08-31	raccoon	female	adult	SAW	shot	not specified	no significant findings	none
16410306436	2016-09-05	raccoon	female	adult	SK	shot	not specified	no significant findings	none
16410307925	2016-09-05	red fox	female	adult	SAW	shot	not specified	no significant findings	none
16410307933	2016-09-05	red fox	male	adult	SAW	shot	not specified	no significant findings	none
16410308863	2016-09-07	red fox	male	adult	MSH	shot	not specified	mixed meningoencephalitis, gliosis, satellitosis, neuronophagia, neuronal necrosis, vacuolization/demyelination	none
16410308864	2016-09-07	red fox	male	juvenile	MSH	shot	not specified	no significant findings	none
16410308865	2016-09-07	red fox	male	adult	SAW	shot	not specified	no significant findings	none
16410308868	2016-09-07	red fox	male	adult	SAW	shot	not specified	no significant findings	none
16410308873	2016-09-06	raccoon	female	adult	SK	shot	not specified	no significant findings	none
16410315096	2016-09-14	raccoon	male	adult	SAW	shot	not specified	no significant findings	CPV-2 ((RT-)qPCR)
16410316434	2016-09-14	red fox	female	adult	MSH	shot	not specified	gliosis, satellitosis, neuronophagia, neuronal necrosis	none
16410316435	2016-09-14	red fox	male	adult	MSH	shot	not specified	no significant findings	none
16410316436	2016-09-15	red fox	female	adult	SAW	shot	not specified	no significant findings	none
16410316437	2016-09-15	red fox	male	adult	SAW	shot	not specified	no significant findings	none
16410317777	2016-09-15	red fox	male	adult	HZ	shot	not specified	no significant findings	none
16410317782	2016-09-15	red fox	female	adult	HZ	shot	not specified	no significant findings	none
16410319537	2016-09-20	badger	male	adult	SAW	shot	not specified	no significant findings	CDV ((RT-)qPCR)
16410320569	2016-09-21	marten	male	adult	SAW	shot	not specified	no significant findings	CPV-2 ((RT-)qPCR)

Continued on the following page

Lab-ID	Date of death or discovery	Species	Gender	Age	Administrative District	Cause of death	Behavior	Histopathological findings in the brain	Pathogens detected (method)
16410320570	2016-09-20	red fox	female	adult	BLK	shot	not specified	no significant findings	CPV-2 ((RT-)qPCR)
16410320571	2016-09-20	red fox	male	adult	SK	shot	not specified	no significant findings	CDV/CPV-2 ((RT-)qPCR)
16410321555	2016-09-21	red fox	male	adult	BLK	shot	not specified	no significant findings	none
16410321556	2016-09-21	red fox	female	juvenile	SAW	shot	not specified	gliosis	none
16410321557	2016-09-20	red fox	female	adult	SAW	not specified	not specified	no significant findings	none
16410324059	2016-09-24	red fox	male	adult	HZ	shot	not specified	no significant findings	none
16410324069	2016-09-18	red fox	female	adult	WB	shot	not specified	non-suppurative meningoencephalitis	CDV/FoxCV ((RT-)qPCR)
16410325303	2016-09-26	raccoon	female	adult	SK	shot	not specified	no significant findings	none
16410326999	2016-09-27	red fox	male	adult	SLK	shot	not specified	no significant findings	none
16410327000	2016-09-27	red fox	female	adult	SAW	shot	not specified	non-suppurative meningitis	CDV ((RT-)qPCR)
16410330066	2016-09-29	red fox	male	adult	SK	shot	abnormal	no significant findings	CDV ((RT-)qPCR)
16410330931	2016-10-03	red fox	female	adult	SAW	shot	not specified	no significant findings	CDV ((RT-)qPCR)
16410330932	2016-10-03	raccoon	male	juvenile	SAW	shot	not specified	vacuolization/demyelination	CDV ((RT-)qPCR)
16410330936	2016-09-29	raccoon dog	female	adult	SAW	shot	not specified	no significant findings	none
16410332044	2016-10-04	red fox	female	adult	MSH	shot	not specified	non-suppurative encephalitis, satellitosis	CDV ((RT-)qPCR)
16410334636	2016-10-04	red fox	male	adult	BLK	shot	not specified	no significant findings	none
16410335664	2016-10-07	red fox	male	adult	BK	shot	abnormal	gliosis	CDV ((RT-)qPCR)
16410337150	2016-10-08	raccoon	male	adult	ABI	shot	not specified	no significant findings	CDV/CPV-2 ((RT-)qPCR)
16410338436	2016-10-09	red fox	male	adult	HZ	found dead	not specified	no significant findings	CDV ((RT-)qPCR)
16410338437	2016-10-09	red fox	male	adult	HZ	shot	not specified	no significant findings	CDV ((RT-)qPCR)
16410338438	2016-10-09	raccoon	female	adult	SK	shot	not specified	no significant findings	CDV ((RT-)qPCR)
16410340705	2016-10-11	red fox	male	adult	HZ	shot	not specified	non-suppurative meningitis	CDV ((RT-)qPCR)
16410341491	2016-10-13	red fox	male	adult	SK	shot	not specified	non-suppurative encephalitis	none
16410345139	2016-10-14	red fox	female	adult	HZ	shot	not specified	no significant findings	none
16410345140	2016-10-16	red fox	male	adult	BK	shot	abnormal	no significant findings	none

Continued on the following page

Lab-ID	Date of death or discovery	Species	Gender	Age	Administrative District	Cause of death	Behavior	Histopathological findings in the brain	Pathogens detected (method)
16410345141	2016-10-15	red fox	male	adult	HZ	shot	not specified	non-suppurative encephalitis, gliosis, satellitosis, neuronal necrosis, vacuolization/demyelination	none
16410345142	2016-10-15	red fox	female	adult	HZ	shot	not specified	no significant findings	none
16410345143	2016-10-15	raccoon	female	adult	HZ	shot	not specified	no significant findings	none
16410347177	2016-10-14	red fox	male	adult	BK	shot	normal	no significant findings	none
16410347178	2016-10-14	red fox	male	adult	BK	shot	normal	no significant findings	none
16410347179	2016-10-14	red fox	male	adult	SK	shot	normal	non-suppurative meningitis, vacuolization/demyelination	CDV ((RT-)qPCR)
16410347182	2016-10-15	red fox	male	adult	SAW	shot	not specified	no significant findings	none
16410347183	2016-10-16	red fox	male	adult	SAW	shot	not specified	no significant findings	none
16410347184	2016-10-16	red fox	male	adult	SAW	shot	not specified	no significant findings	none
16410352866	2016-10-20	raccoon	male	adult	BK	shot	normal	no significant findings	FoxCV ((RT-)qPCR)
16410352867	2016-10-20	red fox	male	juvenile	MSH	shot	abnormal	no significant findings	FoxCV ((RT-)qPCR)
16410354972	2016-10-21	raccoon dog	female	adult	SAW	shot	not specified	no significant findings	none
16410354973	2016-10-23	red fox	male	adult	ABI	shot	not specified	no significant findings	none
16410354974	2016-10-24	red fox	male	adult	BK	not specified	not specified	no significant findings	FoxCV ((RT-)qPCR)
16410354975	2016-10-24	red fox	male	adult	BK	not specified	not specified	no significant findings	none
16410354976	2016-10-24	red fox	male	adult	WB	shot	not specified	no significant findings	none
16410357433	2016-10-23	raccoon dog	male	adult	SAW	shot	not specified	no significant findings	none
16410357434	2016-10-22	red fox	female	adult	SAW	shot	not specified	no significant findings	none
16410363339	2016-10-24	red fox	male	juvenile	HZ	found dead	not specified	non-suppurative meningitis, gliosis, vacuolization/demyelination	CDV ((RT-)qPCR)
16410363340	2016-10-25	red fox	male	adult	ABI	found dead	not specified	non-suppurative meningitis	CDV ((RT-)qPCR)
16410364735	2016-10-27	red fox	male	adult	SAW	shot	not specified	no significant findings	none

Continued on the following page

Lab-ID	Date of death or discovery	Species	Gender	Age	Administrative District	Cause of death	Behavior	Histopathological findings in the brain	Pathogens detected (method)
16410364736	2016-10-27	raccoon dog	male	adult	SAW	shot	not specified	non-suppurative encephalitis	none
16410364737	2016-10-27	raccoon dog	female	adult	SAW	shot	not specified	no significant findings	none
16410366714	2016-10-29	red fox	female	adult	HZ	shot	not specified	non-suppurative meningitis	none
16410366772	2016-10-29	red fox	female	adult	HZ	shot	not specified	no significant findings	none
16410366773	2016-10-29	red fox	male	adult	HZ	shot	not specified	no significant findings	none
16410366774	2016-10-30	red fox	male	adult	SAW	shot	not specified	no significant findings	none
16410366792	2016-10-31	red fox	male	adult	SK	shot	not specified	non-suppurative meningitis, gliosis, vacuolization/demyelination	none
16410366868	2016-11-01	red fox	male	adult	SDL	shot	not specified	no significant findings	none
16410368159	2016-10-31	raccoon	female	adult	SK	shot	not specified	no significant findings	FoxCV ((RT-)qPCR)
16410368160	2016-10-31	raccoon	female	adult	JL	shot	not specified	no significant findings	none
16410368161	2016-10-31	red fox	male	adult	MSH	shot	not specified	no significant findings	CDV ((RT-)qPCR)
16410368162	2016-11-01	red fox	female	adult	MSH	shot	not specified	non-suppurative meningitis	CDV ((RT-)qPCR)
16410368163	2016-11-01	red fox	male	adult	MSH	shot	not specified	non-suppurative encephalitis, gliosis, satellitosis, vacuolization/demyelination	CDV ((RT-)qPCR)
16410370725	2016-11-02	raccoon	female	adult	SAW	shot	not specified	no significant findings	none
16410370726	2016-10-29	red fox	female	adult	HZ	shot	normal	vacuolization/demyelination	none
16410370727	2016-10-29	red fox	male	adult	HZ	shot	normal	no significant findings	none
16410373275	2016-11-03	raccoon	female	adult	SK	shot	not specified	no significant findings	none
16410373276	2016-11-03	red fox	female	adult	SAW	shot	not specified	no significant findings	none
16410373280	2016-11-03	red fox	male	adult	SAW	shot	not specified	no significant findings	none
16410374457	2016-11-06	raccoon	female	adult	BK	shot	not specified	no significant findings	CPV-2 ((RT-)qPCR)
16410374458	2016-11-04	red fox	male	adult	HZ	shot	not specified	no significant findings	none
16410374459	2016-11-04	red fox	female	adult	HZ	shot	not specified	no significant findings	none
16410374460	2016-11-05	red fox	female	adult	HZ	shot	not specified	no significant findings	none
16410374461	2016-11-06	red fox	male	adult	BK	shot	not specified	no significant findings	none
16410374462	2016-11-06	red fox	male	adult	BK	shot	not specified	no significant findings	FoxCV ((RT-)qPCR)

Continued on the following page

Lab-ID	Date of death or discovery	Species	Gender	Age	Administrative District	Cause of death	Behavior	Histopathological findings in the brain	Pathogens detected (method)
16410374463	2016-11-05	red fox	female	adult	BLK	shot	not specified	gliosis, vacuolization/demyelination	none
16410374469	2016-11-05	red fox	female	adult	BLK	shot	not specified	no significant findings	none
16410377034	2016-11-05	red fox	male	adult	HZ	shot	not specified	no significant findings	none
16410377035	2016-11-05	red fox	male	adult	HZ	shot	not specified	non-suppurative meningitis	none
16410377036	2016-11-05	red fox	male	adult	HZ	shot	not specified	no significant findings	none
16410377037	2016-11-05	red fox	female	adult	HZ	shot	not specified	no significant findings	none
16410377042	2016-11-08	red fox	female	adult	SK	shot	abnormal	non-suppurative meningoencephalitis, vacuolization/demyelination	CDV ((RT-)qPCR)
16410377043	2016-11-06	raccoon	female	adult	BK	shot	not specified	no significant findings	none
16410377045	2016-11-06	red fox	female	adult	BK	shot	not specified	no significant findings	FoxCV ((RT-)qPCR)
16410377046	2016-11-07	red fox	female	adult	MSH	shot	not specified	no significant findings	FoxCV ((RT-)qPCR)
16410377047	2016-11-08	red fox	male	adult	MSH	shot	abnormal	gliosis	CDV ((RT-)qPCR)
16410377048	2016-11-08	red fox	male	adult	SDL	shot	abnormal	no significant findings	CDV ((RT-)qPCR)
16410379193	2016-11-08	raccoon	female	adult	JL	shot	not specified	no significant findings	none
16410379194	2016-11-09	red fox	male	adult	MSH	shot	not specified	no significant findings	CDV ((RT-)qPCR)
16410379195	2016-11-08	red fox	female	adult	SAW	found dead	not specified	no significant findings	CDV ((RT-)qPCR)
16410379476	2016-11-08	raccoon	female	adult	JL	shot	not specified	no significant findings	none
16410380865	2016-11-09	red fox	male	adult	SLK	shot	not specified	gliosis, satellitosis, neuronophagia, neuronal necrosis	CDV ((RT-)qPCR)
16410380866	2016-11-09	red fox	male	adult	SK	shot	not specified	no significant findings	CDV ((RT-)qPCR)
16410380867	2016-11-09	red fox	female	adult	HZ	shot	not specified	non-suppurative meningoencephalitis, gliosis	CDV ((RT-)qPCR)
16410380868	2016-11-09	red fox	male	adult	HZ	shot	not specified	no significant findings	none
16410380869	2016-11-09	red fox	male	adult	HZ	shot	not specified	no significant findings	none
16410380870	2016-11-09	red fox	male	adult	MSH	shot	not specified	non-suppurative meningitis, gliosis, satellitosis, neuronophagia, neuronal necrosis	none

Continued on the following page

Lab-ID	Date of death or discovery	Species	Gender	Age	Administrative District	Cause of death	Behavior	Histopathological findings in the brain	Pathogens detected (method)
16410380967	2016-11-09	red fox	male	adult	MSH	shot	not specified	no significant findings	none
16410383372	2016-11-11	red fox	female	adult	MSH	shot	not specified	non-suppurative meningitis	FoxCV ((RT-)qPCR)
16410383373	2016-11-11	red fox	male	adult	MSH	shot	not specified	non-suppurative meningoencephalitis, gliosis, satellitosis	none
16410383374	2016-11-11	red fox	female	adult	MSH	shot	not specified	no significant findings	FoxCV ((RT-)qPCR)
16410383375	2016-11-11	red fox	female	adult	MSH	shot	not specified	no significant findings	none
16410383380	2016-11-11	red fox	male	adult	MSH	shot	not specified	no significant findings	none
16410383381	2016-10-15	red fox	female	adult	SAW	shot	not specified	no significant findings	FoxCV ((RT-)qPCR)
16410383382	2016-11-01	raccoon dog	female	adult	ABI	shot	not specified	gliosis, satellitosis, neuronophagia	CDV/FoxCV ((RT-)qPCR)
16410384280	2016-11-13	red fox	female	adult	SAW	shot	not specified	no significant findings	none
16410384281	2016-11-11	red fox	female	adult	SK	shot	not specified	no significant findings	CPV-2 ((RT-)qPCR)
16410384282	2016-11-11	red fox	male	adult	SK	shot	not specified	no significant findings	none
16410384283	2016-11-13	red fox	female	adult	BK	shot	not specified	granulomatous encephalitis, neuronal necrosis	larvae of nematodes (HE)
16410384380	2016-11-11	red fox	female	adult	MSH	shot	not specified	no significant findings	none
16410384381	2016-11-11	red fox	male	adult	MSH	shot	not specified	non-suppurative meningitis	none
16410384382	2016-11-11	red fox	male	adult	MSH	shot	not specified	no significant findings	none
16410384383	2016-11-11	red fox	female	adult	MSH	shot	not specified	no significant findings	none
16410384384	2016-11-11	red fox	female	adult	MSH	shot	not specified	non-suppurative meningoencephalitis	none
16410384385	2016-11-14	raccoon	female	adult	MSH	shot	not specified	no significant findings	none
16410385759	2016-11-15	red fox	male	adult	SAW	shot	not specified	no significant findings	none
16410385958	2016-11-14	red fox	male	adult	MSH	shot	not specified	no significant findings	none
16410385959	2016-11-14	raccoon	female	adult	MSH	shot	not specified	granulomatous encephalitis	CDV ((RT-)qPCR), larvae of nematodes (HE)
16410385960	2016-11-14	red fox	female	adult	MSH	shot	not specified	no significant findings	CDV ((RT-)qPCR)
16410385965	2016-11-13	red fox	female	adult	MSH	shot	not specified	non-suppurative encephalitis	none
16410385966	2016-11-11	red fox	male	adult	HZ	shot	not specified	non-suppurative encephalitis, gliosis	none

Continued on the following page

Lab-ID	Date of death or discovery	Species	Gender	Age	Administrative District	Cause of death	Behavior	Histopathological findings in the brain	Pathogens detected (method)
16410385967	2016-11-11	red fox	male	adult	HZ	shot	not specified	no significant findings	none
16410385968	2016-11-14	raccoon	female	adult	SAW	shot	not specified	no significant findings	none
16410385969	2016-11-14	raccoon	female	adult	SAW	shot	not specified	no significant findings	none
16410388461	2016-11-12	red fox	male	adult	HZ	shot	not specified	no significant findings	none
16410388462	2016-11-12	red fox	male	adult	HZ	shot	not specified	no significant findings	none
16410388463	2016-11-12	red fox	female	adult	HZ	shot	not specified	mixed meningoencephalitis	larvae of nematodes (HE)
16410388464	2016-11-12	red fox	female	adult	HZ	shot	not specified	no significant findings	none
16410390505	2016-11-15	red fox	male	adult	MSH	shot	not specified	no significant findings	none
16410390602	2016-11-12	red fox	male	adult	HZ	shot	not specified	no significant findings	none
16410390603	2016-11-11	red fox	female	adult	HZ	shot	not specified	non-suppurative encephalitis, gliosis, satellitosis	none
16410390604	2016-11-12	red fox	male	adult	HZ	shot	not specified	no significant findings	none
16410390605	2016-11-13	red fox	female	adult	BK	shot	not specified	no significant findings	none
16410390606	2016-11-13	red fox	female	adult	BK	shot	not specified	non-suppurative meningoencephalitis, gliosis	none
16410394425	2016-11-17	red fox	female	adult	MSH	shot	not specified	no significant findings	none
16410394426	2016-11-18	red fox	female	adult	BLK	found dead	not specified	no significant findings	none
16410394427	2016-11-17	raccoon	female	adult	SK	shot	not specified	no significant findings	none
16410398923	2016-11-20	red fox	male	adult	MSH	shot	not specified	no significant findings	none
16410398924	2016-11-19	red fox	female	adult	HZ	shot	not specified	no significant findings	none
16410398925	2016-11-19	red fox	female	adult	BK	shot	not specified	no significant findings	none
16410398926	2016-11-19	red fox	female	adult	BK	shot	not specified	no significant findings	none
16410398929	2016-11-20	red fox	male	adult	HZ	shot	not specified	non-suppurative encephalitis, gliosis	none
16410398930	2016-11-18	red fox	female	adult	MSH	shot	not specified	no significant findings	none
16410398931	2016-11-20	red fox	female	adult	ABI	shot	not specified	no significant findings	none
16410398932	2016-11-21	raccoon	female	adult	BLK	shot	not specified	non-suppurative encephalitis, gliosis	CPV-2 ((RT-)qPCR)

Continued on the following page

Lab-ID	Date of death or discovery	Species	Gender	Age	Administrative District	Cause of death	Behavior	Histopathological findings in the brain	Pathogens detected (method)
16410402528	2016-11-22	raccoon	female	adult	ABI	shot	not specified	no significant findings	none
16410402625	2016-11-22	red fox	female	adult	SAW	shot	not specified	no significant findings	CDV ((RT-)qPCR)
16410402626	2016-11-22	marten	male	adult	SAW	shot	not specified	no significant findings	none
16410403923	2016-11-24	red fox	male	adult	MSH	shot	not specified	no significant findings	CDV ((RT-)qPCR)
16410413105	2016-11-24	red fox	male	adult	SK	shot	not specified	no significant findings	none
16410413106	2016-11-25	red fox	female	adult	SK	shot	not specified	no significant findings	CDV ((RT-)qPCR)
16410414507	2016-11-25	red fox	male	adult	HZ	shot	not specified	vacuolization/demyelination	none
16410414606	2016-11-25	red fox	male	adult	HZ	shot	not specified	no significant findings	none
16410414608	2016-11-26	red fox	male	adult	HZ	shot	not specified	no significant findings	none
16410414610	2016-11-28	red fox	male	adult	MSH	shot	not specified	non-suppurative meningitis, vacuolization/demyelination	CDV ((RT-)qPCR)
16410414614	2016-11-28	red fox	male	adult	SK	shot	not specified	no significant findings	none
16410417055	2016-11-26	red fox	male	adult	HZ	shot	not specified	no significant findings	none
16410417056	2016-11-26	red fox	male	adult	HZ	shot	not specified	no significant findings	none
16410417057	2016-11-23	red fox	male	adult	HZ	shot	not specified	no significant findings	none
16410417058	2016-11-28	red fox	female	adult	MSH	shot	not specified	no significant findings	none
16410417059	2016-11-28	red fox	male	adult	MSH	shot	not specified	no significant findings	none
16410421068	2016-11-30	red fox	male	adult	HZ	shot	not specified	non-suppurative meningoencephalitis, gliosis	CDV ((RT-)qPCR)
16410421070	2016-11-30	red fox	male	adult	HZ	shot	not specified	non-suppurative meningoencephalitis, gliosis, satellitosis, neuronophagia, neuronal necrosis, vacuolization/demyelination	none
16410421072	2016-11-29	red fox	female	adult	HZ	shot	not specified	no significant findings	CDV ((RT-)qPCR)
16410421075	2016-11-30	red fox	male	adult	DE	shot	not specified	no significant findings	none
16410421092	2016-11-30	red fox	male	adult	BLK	shot	abnormal	no significant findings	none
16410421093	2016-11-29	raccoon dog	male	adult	SAW	shot	not specified	no significant findings	none

Continued on the following page

Lab-ID	Date of death or discovery	Species	Gender	Age	Administrative District	Cause of death	Behavior	Histopathological findings in the brain	Pathogens detected (method)
16410423548	2016-12-03	red fox	male	adult	SAW	shot	not specified	no significant findings	CPV-2 ((RT-)qPCR)
16410423549	2016-12-03	red fox	male	adult	SAW	shot	not specified	no significant findings	none
16410423550	2016-12-03	red fox	male	adult	SAW	shot	not specified	no significant findings	CPV-2 ((RT-)qPCR)
16410423551	2016-12-03	red fox	female	adult	SAW	shot	not specified	no significant findings	none
16410423552	2016-12-03	red fox	male	adult	SAW	shot	not specified	no significant findings	none
16410423553	2016-12-03	red fox	male	adult	SAW	shot	not specified	no significant findings	CDV ((RT-)qPCR)
16410423554	2016-12-03	red fox	male	adult	SAW	shot	not specified	no significant findings	CPV-2 ((RT-)qPCR)
16410424052	2016-12-02	red fox	male	adult	HZ	shot	not specified	no significant findings	CPV-2 ((RT-)qPCR)
16410424054	2016-12-02	red fox	male	adult	HZ	shot	not specified	gliosis, satellitosis, neuronophagia, neuronal necrosis	none
16410424056	2016-12-02	red fox	male	adult	HZ	shot	not specified	no significant findings	none
16410424057	2016-12-02	red fox	female	adult	HZ	shot	not specified	no significant findings	none
16410424059	2016-12-02	red fox	female	adult	HZ	shot	not specified	non-suppurative meningoencephalitis	none
16410424061	2016-12-02	red fox	female	adult	HZ	shot	not specified	non-suppurative encephalitis, gliosis, satellitosis, neuronophagia, neuronal necrosis	none
16410424062	2016-12-03	red fox	female	adult	MSH	shot	not specified	no significant findings	none
16410426737	2016-12-05	red fox	male	adult	HZ	shot	not specified	no significant findings	none
16410426757	2016-12-01	red fox	female	adult	BK	shot	not specified	no significant findings	none
16410426879	2016-12-06	red fox	female	adult	ABI	shot	abnormal	granulomatous encephalitis	CDV ((RT-)qPCR), larvae of nematodes (HE)
16410426880	2016-12-05	red fox	male	adult	SK	shot	abnormal	mixed meningoencephalitis, satellitosis	none
16410426882	2016-12-04	red fox	female	adult	MSH	shot	not specified	no significant findings	none
16410426883	2016-12-03	red fox	male	adult	SAW	shot	not specified	no significant findings	FoxCV ((RT-)qPCR)
16410426886	2016-12-03	red fox	female	adult	SAW	shot	not specified	no significant findings	none
16410426890	2016-12-03	red fox	male	adult	SDL	shot	not specified	no significant findings	none
16410429655	2016-12-03	red fox	male	adult	SAW	shot	abnormal	no significant findings	FoxCV ((RT-)qPCR)

Continued on the following page

Lab-ID	Date of death or discovery	Species	Gender	Age	Administrative District	Cause of death	Behavior	Histopathological findings in the brain	Pathogens detected (method)
16410429742	2016-12-02	raccoon dog	male	adult	SAW	shot	not specified	no significant findings	none
16410429963	2016-12-05	red fox	female	adult	SAW	shot	not specified	no significant findings	none
16410433430	2016-12-03	red fox	male	adult	ABI	shot	normal	no significant findings	CDV/FoxCV ((RT-)qPCR)
16410433431	2016-12-07	red fox	male	adult	HZ	shot	abnormal	non-suppurative encephalitis	CDV ((RT-)qPCR), *Salmonella enterica* subsp. *enterica* (BE)
16410433433	2016-12-07	red fox	female	adult	SK	shot	abnormal	non-suppurative meningitis, gliosis, satellitosis, neuronophagia	CDV ((RT-)qPCR)
16410436813	2016-12-09	red fox	male	adult	ABI	found dead	not specified	no significant findings	CDV/FoxCV ((RT-)qPCR)
16410438702	2016-12-11	red fox	female	adult	SK	shot	not specified	non-suppurative meningitis	none
16410438703	2016-12-10	red fox	male	adult	HZ	shot	abnormal	no significant findings	CDV ((RT-)qPCR)
16410438715	2016-12-08	marten	male	adult	BK	shot	abnormal	non-suppurative encephalitis	none
16410441522	2016-12-10	red fox	male	adult	SAW	shot	abnormal	non-suppurative meningitis	CDV/FoxCV ((RT-)qPCR)
16410441523	2016-12-12	red fox	male	adult	SK	shot	not specified	no significant findings	CPV-2 ((RT-)qPCR)
16410441524	2016-12-12	red fox	female	adult	BLK	shot	not specified	vacuolization/demyelination	none
16410441625	2016-12-12	red fox	female	adult	BLK	shot	not specified	no significant findings	none
16410441639	2016-12-12	raccoon	male	adult	ABI	found dead	abnormal	no significant findings	CDV ((RT-)qPCR)
16410441640	2016-12-09	raccoon	female	adult	SAW	shot	not specified	no significant findings	none
16410441675	2016-12-12	red fox	female	adult	BLK	shot	not specified	non-suppurative meningoencephalitis, gliosis, vacuolization/demyelination	CDV ((RT-)qPCR)
16410441676	2016-12-12	red fox	female	adult	BLK	shot	not specified	non-suppurative meningitis, vacuolization/demyelination	none
16410441773	2016-12-09	raccoon	male	adult	SAW	shot	not specified	no significant findings	none
16410445875	2016-12-15	red fox	female	adult	SAW	shot	not specified	no significant findings	none
16410445877	2016-12-15	red fox	female	adult	SAW	shot	not specified	non-suppurative meningitis	none
16410447822	2016-12-14	red fox	female	adult	MSH	shot	not specified	no significant findings	CPV-2 ((RT-)qPCR)
16410448955	2016-12-16	red fox	female	adult	HZ	shot	not specified	no significant findings	none

Continued on the following page

Lab-ID	Date of death or discovery	Species	Gender	Age	Administrative District	Cause of death	Behavior	Histopathological findings in the brain	Pathogens detected (method)
16410448956	2016-12-16	red fox	female	adult	HZ	shot	not specified	non-suppurative meningoencephalitis	none
16410448957	2016-12-16	red fox	female	adult	MSH	shot	not specified	non-suppurative meningoencephalitis, vacuolization/demyelination	CDV ((RT-)qPCR)
16410448958	2016-12-16	red fox	male	adult	MSH	shot	not specified	no significant findings	none
16410448959	2016-12-17	red fox	male	adult	SAW	shot	not specified	no significant findings	none
16410448960	2016-12-17	red fox	male	adult	SAW	shot	not specified	no significant findings	none
16410451813	2016-12-20	red fox	male	adult	SLK	found dead	not specified	no significant findings	CDV ((RT-)qPCR)
16410451837	2016-12-18	red fox	male	adult	BLK	shot	not specified	no significant findings	FoxCV ((RT-)qPCR)
16410455151	2016-12-19	red fox	female	adult	SAW	shot	not specified	no significant findings	none
16410459817	2016-12-22	red fox	male	adult	MSH	shot	abnormal	no significant findings	CDV ((RT-)qPCR)
16410461371	2016-12-24	red fox	male	adult	HZ	shot	abnormal	no significant findings	CDV ((RT-)qPCR)
16410463201	2016-12-25	red fox	female	adult	SAW	shot	abnormal	no significant findings	none
16410463298	2016-12-28	red fox	male	adult	SDL	shot	not specified	no significant findings	none
16410464409	2016-12-29	red fox	female	adult	ABI	shot	abnormal	granulomatous encephalitis, gliosis, vacuolization/demyelination	CDV/CPV-2 ((RT-)qPCR)
17410000156	2016-12-30	red fox	male	adult	SK	found dead	not specified	no significant findings	none
17410002190	2017-01-03	red fox	male	adult	SAW	shot	not specified	non-suppurative meningitis, gliosis, satellitosis, neuronophagia, vacuolization/demyelination	none
17410002191	2017-01-03	raccoon dog	female	adult	MSH	shot	not specified	non-suppurative meningoencephalitis, gliosis	CDV ((RT-)qPCR)
17410002967	2017-01-04	red fox	male	adult	JL	shot	not specified	no significant findings	none
17410002968	2017-01-02	red fox	female	adult	BLK	found dead	not specified	non-suppurative meningitis, vacuolization/demyelination	none
17410002969	2017-01-04	red fox	male	adult	MSH	shot	not specified	no significant findings	none
17410005956	2017-01-07	red fox	female	juvenile	HZ	shot	not specified	no significant findings	none

Continued on the following page

Lab-ID	Date of death or discovery	Species	Gender	Age	Administrative District	Cause of death	Behavior	Histopathological findings in the brain	Pathogens detected (method)
17410005957	2017-01-07	red fox	male	adult	HZ	shot	not specified	no significant findings	none
17410005958	2017-01-06	red fox	female	adult	HZ	shot	not specified	no significant findings	none
17410005959	2017-01-07	red fox	male	adult	BLK	shot	not specified	no significant findings	none
17410005961	2017-01-06	red fox	male	adult	SAW	shot	not specified	no significant findings	FoxCV ((RT-)qPCR)
17410007543	2017-01-09	red fox	male	adult	SAW	shot	not specified	no significant findings	CPV-2 ((RT-)qPCR)
17410007546	2017-01-09	red fox	female	adult	SAW	shot	not specified	no significant findings	CPV-2 ((RT-)qPCR)
17410007547	2017-01-07	red fox	male	adult	SAW	shot	not specified	no significant findings	none
17410007548	2017-01-07	red fox	male	adult	SAW	shot	not specified	no significant findings	none
17410007549	2017-01-07	red fox	male	adult	BLK	shot	not specified	no significant findings	none
17410007550	2017-01-09	red fox	female	adult	MSH	shot	abnormal	non-suppurative meningoencephalitis, gliosis, satellitosis	CDV ((RT-)qPCR)
17410007551	2017-01-07	red fox	female	adult	HZ	shot	not specified	no significant findings	CDV ((RT-)qPCR)
17410007552	2017-01-06	red fox	female	adult	HZ	shot	not specified	no significant findings	CDV ((RT-)qPCR)
17410007553	2017-01-08	red fox	male	adult	MSH	shot	not specified	no significant findings	none
17410007554	2017-01-06	red fox	male	adult	MSH	shot	not specified	non-suppurative meningitis, gliosis	none
17410007555	2017-01-09	red fox	male	adult	SK	shot	not specified	no significant findings	none
17410009809	2017-01-10	red fox	female	adult	SK	shot	not specified	no significant findings	none
17410009815	2017-01-07	red fox	male	adult	SAW	shot	not specified	no significant findings	none
17410009816	2017-01-07	red fox	male	adult	SAW	shot	not specified	no significant findings	none
17410012231	2017-01-12	red fox	male	adult	SK	shot	not specified	no significant findings	none
17410015841	2017-01-13	red fox	male	adult	MSH	shot	not specified	granulomatous encephalitis, vacuolization/demyelination	larvae of nematodes (HE)
17410015842	2017-01-13	red fox	male	adult	MSH	shot	not specified	mixed meningoencephalitis, vacuolization/demyelination	larvae of nematodes (HE)
17410015845	2017-01-13	red fox	male	adult	MSH	shot	not specified	no significant findings	none
17410015846	2017-01-13	red fox	female	adult	MSH	shot	not specified	granulomatous encephalitis, gliosis, vacuolization/demyelination	CDV ((RT-)qPCR), larvae of nematodes (HE)

Continued on the following page

Lab-ID	Date of death or discovery	Species	Gender	Age	Administrative District	Cause of death	Behavior	Histopathological findings in the brain	Pathogens detected (method)
17410015847	2017-01-13	red fox	female	adult	MSH	shot	not specified	non-suppurative meningitis, gliosis, satellitosis, vacuolization/demyelination	CDV ((RT-)qPCR)
17410016586	2017-01-10	red fox	male	adult	SAW	shot	not specified	no significant findings	none
17410016587	2017-01-14	red fox	male	adult	SAW	shot	not specified	vacuolization/demyelination	FoxCV ((RT-)qPCR)
17410016591	2017-01-16	red fox	female	adult	MSH	shot	not specified	non-suppurative meningitis, gliosis, vacuolization/demyelination	CDV ((RT-)qPCR)
17410016593	2017-01-14	red fox	female	adult	SK	shot	not specified	no significant findings	none
17410016594	2017-01-15	red fox	male	adult	SK	shot	not specified	no significant findings	CDV ((RT-)qPCR)
17410016595	2017-01-14	red fox	female	adult	SK	shot	not specified	non-suppurative meningoencephalitis, gliosis	none
17410016596	2017-01-14	red fox	male	adult	SK	shot	not specified	non-suppurative encephalitis	none
17410016597	2017-01-14	red fox	female	adult	SAW	shot	not specified	vacuolization/demyelination	none
17410016598	2017-01-14	red fox	male	adult	SAW	shot	not specified	vacuolization/demyelination	CDV ((RT-)qPCR)
17410016599	2017-01-15	red fox	male	adult	SK	shot	not specified	non-suppurative meningitis, vacuolization/demyelination	none
17410018321	2017-01-14	red fox	male	adult	SAW	shot	not specified	no significant findings	CDV ((RT-)qPCR)
17410018322	2017-01-14	red fox	female	adult	SAW	shot	not specified	no significant findings	CDV ((RT-)qPCR)
17410018323	2017-01-16	red fox	female	adult	BLK	shot	not specified	no significant findings	none
17410018324	2017-01-17	red fox	female	adult	BLK	shot	not specified	no significant findings	none
17410018329	2017-01-14	red fox	female	adult	SAW	shot	not specified	non-suppurative encephalitis, gliosis, satellitosis	CDV ((RT-)qPCR)
17410020393	2017-01-14	red fox	female	adult	SAW	shot	not specified	non-suppurative encephalitis	none
17410020394	2017-01-16	red fox	male	adult	MSH	shot	not specified	no significant findings	none
17410020491	2017-01-17	red fox	male	adult	JL	shot	not specified	no significant findings	*Salmonella enterica* subsp. *diarizonae* (BE)
17410021677	2017-01-14	red fox	female	adult	ABI	shot	not specified	no significant findings	none
17410021678	2017-01-17	red fox	female	adult	SK	shot	not specified	no significant findings	CDV ((RT-)qPCR)

Continued on the following page

Lab-ID	Date of death or discovery	Species	Gender	Age	Administrative District	Cause of death	Behavior	Histopathological findings in the brain	Pathogens detected (method)
17410021679	2017-01-18	red fox	male	adult	MSH	shot	not specified	non-suppurative meningoencephalitis, gliosis	none
17410022776	2017-01-19	red fox	male	adult	MSH	shot	abnormal	non-suppurative meningitis	CDV ((RT-)qPCR)
17410022777	2017-01-18	red fox	female	adult	MSH	shot	not specified	no significant findings	CDV ((RT-)qPCR)
17410022778	2017-01-19	red fox	female	adult	MSH	found dead	abnormal	no significant findings	CDV ((RT-)qPCR)
17410024525	2017-01-21	red fox	female	adult	SDL	shot	not specified	no significant findings	none
17410024526	2017-01-21	red fox	male	adult	SDL	shot	not specified	no significant findings	none
17410024527	2017-01-21	red fox	female	adult	SDL	shot	not specified	no significant findings	none
17410024528	2017-01-21	red fox	female	adult	SDL	shot	not specified	no significant findings	none
17410024529	2017-01-21	red fox	female	adult	SDL	shot	not specified	no significant findings	none
17410024625	2017-01-21	red fox	female	adult	SDL	shot	not specified	no significant findings	none
17410024626	2017-01-21	red fox	male	adult	SDL	shot	not specified	no significant findings	none
17410025653	2017-01-20	red fox	female	adult	HZ	shot	not specified	non-suppurative meningoencephalitis, gliosis, vacuolization/demyelination	none
17410025654	2017-01-22	red fox	male	adult	HZ	shot	abnormal	gliosis, satellitosis	CDV ((RT-)qPCR)
17410025655	2017-01-20	red fox	female	adult	SAW	shot	not specified	no significant findings	CDV ((RT-)qPCR)
17410025656	2017-01-20	red fox	male	adult	ABI	shot	not specified	non-suppurative meningoencephalitis, vacuolization/demyelination	CDV/FoxCV ((RT-)qPCR)
17410025657	2017-01-21	red fox	male	adult	MSH	shot	abnormal	non-suppurative meningitis	CDV ((RT-)qPCR)
17410025658	2017-01-22	red fox	male	adult	MSH	shot	not specified	no significant findings	CDV ((RT-)qPCR)
17410025659	2017-01-22	red fox	male	adult	MSH	shot	not specified	no significant findings	CDV/FoxCV ((RT-)qPCR)
17410027001	2017-01-16	red fox	female	adult	MSH	shot	abnormal	no significant findings	none
17410027002	2017-01-20	red fox	male	adult	HZ	shot	not specified	no significant findings	none
17410027005	2017-01-23	red fox	male	adult	MSH	found dead	abnormal	gliosis, satellitosis, vacuolization/demyelination	CDV ((RT-)qPCR)
17410027009	2017-01-23	red fox	male	adult	MSH	shot	not specified	gliosis, vacuolization/demyelination	CDV ((RT-)qPCR)

Continued on the following page

Lab-ID	Date of death or discovery	Species	Gender	Age	Administrative District	Cause of death	Behavior	Histopathological findings in the brain	Pathogens detected (method)
17410027010	2017-01-23	red fox	male	adult	BK	found dead	not specified	no significant findings	none
17410027011	2017-01-22	red fox	male	adult	SAW	shot	not specified	no significant findings	none
17410027012	2017-01-23	red fox	male	adult	ABI	shot	abnormal	vacuolization/demyelination	CDV ((RT-)qPCR)
17410029237	2017-01-24	red fox	female	adult	MSH	shot	abnormal	non-suppurative meningitis	CDV ((RT-)qPCR)
17410029238	2017-01-25	red fox	female	adult	MSH	shot	abnormal	non-suppurative meningitis, gliosis	CDV ((RT-)qPCR)
17410029239	2017-01-24	red fox	male	adult	MSH	found dead	abnormal	vacuolization/demyelination	CDV ((RT-)qPCR)
17410031486	2017-01-22	red fox	male	adult	MSH	shot	abnormal	non-suppurative meningitis	CDV ((RT-)qPCR)
17410031487	2017-01-25	raccoon	male	adult	BK	shot	not specified	non-suppurative encephalitis, gliosis, vacuolization/demyelination	CDV ((RT-)qPCR)
17410031489	2017-01-23	red fox	male	adult	BK	shot	not specified	no significant findings	none
17410031494	2017-01-25	red fox	male	adult	MSH	shot	abnormal	granulomatous encephalitis, gliosis, vacuolization/demyelination	CDV ((RT-)qPCR), larvae of nematodes (HE)
17410031498	2017-01-26	red fox	female	adult	SK	shot	not specified	gliosis, satellitosis	CDV ((RT-)qPCR)
17410031503	2017-01-25	red fox	female	adult	MSH	found dead	not specified	no significant findings	none
17410031504	2017-01-24	red fox	female	adult	MSH	shot	not specified	no significant findings	CDV ((RT-)qPCR)
17410034554	2017-01-28	red fox	female	adult	MSH	shot	not specified	non-suppurative meningitis, gliosis, satellitosis, neuronophagia, neuronal necrosis, vacuolization/demyelination	CDV ((RT-)qPCR)
17410034575	2017-01-28	red fox	male	adult	MSH	shot	not specified	non-suppurative meningitis	CDV ((RT-)qPCR)
17410034576	2017-01-28	red fox	female	adult	MSH	shot	not specified	non-suppurative encephalitis, gliosis, satellitosis	CDV ((RT-)qPCR)
17410034577	2017-01-29	raccoon	male	adult	SK	shot	not specified	no significant findings	CPV-2 ((RT-)qPCR)
17410034578	2017-01-28	raccoon	male	adult	SAW	shot	not specified	no significant findings	none
17410034579	2017-01-28	red fox	female	adult	SAW	shot	not specified	vacuolization/demyelination	none
17410034580	2017-01-28	red fox	male	adult	HZ	shot	not specified	non-suppurative meningitis, gliosis, vacuolization/demyelination	none

Continued on the following page

Lab-ID	Date of death or discovery	Species	Gender	Age	Administrative District	Cause of death	Behavior	Histopathological findings in the brain	Pathogens detected (method)
17410034581	2017-01-29	red fox	male	adult	HZ	shot	not specified	vacuolization/demyelination	none
17410034582	2017-01-29	red fox	male	adult	HZ	shot	not specified	no significant findings	none
17410034679	2017-01-30	red fox	female	adult	HZ	shot	not specified	no significant findings	none
17410035803	2017-01-29	raccoon	male	adult	BLK	shot	not specified	no significant findings	CPV-2 ((RT-)qPCR)
17410035804	2017-01-31	red fox	female	adult	SK	shot	not specified	non-suppurative meningitis	none
17410035806	2017-01-29	red fox	male	adult	SAW	shot	not specified	no significant findings	none
17410035807	2017-01-29	red fox	female	adult	BLK	shot	not specified	gliosis, vacuolization/demyelination	none
17410035808	2017-01-27	red fox	female	adult	SAW	shot	not specified	no significant findings	none
17410037560	2017-01-28	red fox	female	adult	BLK	found dead	not specified	no significant findings	none
17410037561	2017-01-29	red fox	female	adult	HZ	shot	not specified	non-suppurative meningoencephalitis	none
17410037562	2017-01-31	red fox	female	adult	MSH	shot	not specified	no significant findings	CDV ((RT-)qPCR)
17410039063	2017-02-01	red fox	male	adult	SAW	shot	abnormal	granulomatous encephalitis, gliosis, satellitosis	CDV ((RT-)qPCR), *T. gondii* (IHC)
17410039064	2017-02-01	red fox	male	adult	SK	shot	not specified	no significant findings	CDV ((RT-)qPCR)
17410039065	2017-02-01	red fox	male	adult	DE	shot	not specified	no significant findings	CDV ((RT-)qPCR)
17410040231	2017-01-29	raccoon	male	adult	BLK	shot	not specified	no significant findings	none
17410040232	2017-01-28	red fox	male	adult	WB	shot	not specified	no significant findings	none
17410040237	2017-02-04	red fox	male	adult	SDL	shot	not specified	no significant findings	none
17410040239	2017-02-04	red fox	female	adult	SDL	shot	not specified	no significant findings	none
17410040240	2017-02-04	raccoon dog	female	adult	SDL	shot	not specified	no significant findings	FoxCV ((RT-)qPCR)
17410040241	2017-02-04	red fox	male	adult	SDL	shot	not specified	no significant findings	none
17410040965	2017-02-04	red fox	female	adult	SDL	shot	not specified	no significant findings	FoxCV ((RT-)qPCR)
17410040973	2017-02-04	red fox	male	adult	SDL	shot	not specified	no significant findings	none
17410042354	2017-02-03	red fox	female	adult	SK	shot	abnormal	non-suppurative meningoencephalitis	CDV/FoxCV ((RT-)qPCR)
17410042355	2017-02-02	raccoon	male	adult	SAW	shot	not specified	no significant findings	none

Continued on the following page

Lab-ID	Date of death or discovery	Species	Gender	Age	Administrative District	Cause of death	Behavior	Histopathological findings in the brain	Pathogens detected (method)
17410044052	2017-02-02	red fox	female	adult	SAW	shot	not specified	non-suppurative encephalitis, vacuolization/demyelination	CDV ((RT-)qPCR)
17410044053	2017-02-03	red fox	female	adult	BLK	shot	abnormal	no significant findings	CDV ((RT-)qPCR)
17410044054	2017-02-07	red fox	male	adult	SAW	not specified	not specified	no significant findings	CDV ((RT-)qPCR)
17410045545	2017-02-07	red fox	male	adult	SAW	shot	not specified	no significant findings	none
17410046720	2017-02-08	red fox	female	adult	HZ	found dead	not specified	no significant findings	CDV ((RT-)qPCR)
17410048825	2017-02-10	red fox	female	adult	MSH	shot	abnormal	non-suppurative meningitis, gliosis	CDV/CPV-2 ((RT-)qPCR)
17410048826	2017-02-09	red fox	female	adult	MSH	shot	not specified	no significant findings	CDV/FoxCV ((RT-)qPCR)
17410049574	2017-02-14	red fox	female	adult	SDL	shot	not specified	granulomatous encephalitis, gliosis	CDV/CPV-2/FoxCV ((RT-)qPCR)
17410050576	2017-02-14	red fox	female	adult	JL	shot	not specified	non-suppurative encephalitis	CDV ((RT-)qPCR)
17410050579	2017-02-10	red fox	female	adult	MSH	shot	not specified	no significant findings	CDV ((RT-)qPCR)
17410050580	2017-02-13	red fox	male	adult	WB	found dead	not specified	gliosis, vacuolization/demyelination	CDV ((RT-)qPCR)
17410050581	2017-02-10	red fox	female	adult	WB	shot	not specified	no significant findings	CDV ((RT-)qPCR)
17410050582	2017-02-11	red fox	female	adult	BLK	shot	not specified	no significant findings	none
17410050583	2017-02-11	red fox	male	adult	MSH	shot	not specified	vacuolization/demyelination	CPV-2 ((RT-)qPCR)
17410050596	2017-02-13	red fox	male	adult	WB	found dead	not specified	no significant findings	CDV ((RT-)qPCR)
17410051204	2017-02-13	raccoon	female	adult	SK	shot	not specified	no significant findings	none
17410051205	2017-02-11	red fox	female	adult	BK	shot	not specified	non-suppurative encephalitis, vacuolization/demyelination	CDV ((RT-)qPCR)
17410051206	2017-02-11	red fox	male	adult	BK	shot	not specified	no significant findings	CDV ((RT-)qPCR)
17410051207	2017-02-13	red fox	male	adult	MSH	shot	abnormal	non-suppurative meningitis	CDV ((RT-)qPCR)
17410051209	2017-02-14	red fox	male	adult	HZ	shot	not specified	no significant findings	none
17410051210	2017-02-13	red fox	male	adult	BK	shot	not specified	no significant findings	CDV ((RT-)qPCR)

Continued on the following page

Lab-ID	Date of death or discovery	Species	Gender	Age	Administrative District	Cause of death	Behavior	Histopathological findings in the brain	Pathogens detected (method)
17410053098	2017-02-11	raccoon dog	male	adult	SAW	shot	not specified	no significant findings	FoxCV ((RT-)qPCR)
17410053099	2017-02-13	red fox	female	adult	MSH	found dead	not specified	no significant findings	CDV ((RT-)qPCR)
17410053100	2017-02-11	red fox	female	adult	SAW	shot	not specified	no significant findings	CDV ((RT-)qPCR)
17410053101	2017-02-11	red fox	female	adult	SAW	shot	not specified	no significant findings	CDV ((RT-)qPCR)
17410053103	2017-02-11	red fox	male	adult	SAW	shot	not specified	no significant findings	none
17410053104	2017-02-11	red fox	female	adult	SAW	shot	not specified	non-suppurative meningitis, vacuolization/demyelination	CDV ((RT-)qPCR)
17410053105	2017-02-11	red fox	female	adult	SAW	shot	not specified	vacuolization/demyelination	none
17410053106	2017-02-14	red fox	female	adult	MSH	shot	not specified	no significant findings	CDV ((RT-)qPCR)
17410053107	2017-02-13	red fox	female	adult	MSH	shot	abnormal	non-suppurative meningitis	CDV ((RT-)qPCR)
17410055888	2017-02-16	red fox	female	adult	SK	found dead	not specified	non-suppurative meningitis	CDV ((RT-)qPCR)
17410055904	2017-02-15	red fox	female	adult	BK	shot	not specified	gliosis, satellitosis, neuronophagia, neuronal necrosis	CDV ((RT-)qPCR)
17410059574	2017-02-19	red fox	male	adult	SAW	shot	not specified	no significant findings	none
17410059575	2017-02-19	red fox	male	adult	BK	shot	not specified	no significant findings	CDV ((RT-)qPCR)
17410059576	2017-02-20	raccoon	female	adult	SK	shot	not specified	non-suppurative meningitis, vacuolization/demyelination	none
17410061544	2017-02-18	red fox	male	adult	SAW	shot	not specified	no significant findings	CDV ((RT-)qPCR)
17410061546	2017-02-18	red fox	male	adult	SAW	shot	not specified	vacuolization/demyelination	none
17410061547	2017-02-18	red fox	female	adult	HZ	shot	not specified	no significant findings	CDV ((RT-)qPCR)
17410061550	2017-02-17	red fox	male	adult	BK	shot	not specified	no significant findings	CDV ((RT-)qPCR)
17410061569	2017-02-20	red fox	female	adult	MSH	shot	not specified	vacuolization/demyelination	CDV ((RT-)qPCR)
17410061572	2017-02-20	raccoon	male	adult	MSH	shot	not specified	no significant findings	CDV ((RT-)qPCR)
17410061576	2017-02-18	red fox	male	adult	SAW	shot	not specified	vacuolization/demyelination	none
17410066529	2017-02-22	red fox	female	adult	HZ	found dead	not specified	no significant findings	CDV ((RT-)qPCR)

Continued on the following page

Lab-ID	Date of death or discovery	Species	Gender	Age	Administrative District	Cause of death	Behavior	Histopathological findings in the brain	Pathogens detected (method)
17410066530	2017-02-20	red fox	male	adult	SAW	shot	not specified	non-suppurative meningitis, gliosis, vacuolization/demyelination	CDV ((RT-)qPCR)
17410066535	2017-02-22	red fox	female	adult	HZ	found dead	not specified	non-suppurative meningitis, gliosis, vacuolization/demyelination	CDV ((RT-)qPCR)
17410066536	2017-02-21	red fox	female	adult	SLK	found dead	not specified	no significant findings	CDV ((RT-)qPCR)
17410068144	2017-02-23	red fox	female	adult	HZ	shot	abnormal	no significant findings	CDV ((RT-)qPCR)
17410068145	2017-02-19	red fox	female	adult	WB	shot	not specified	non-suppurative meningitis, gliosis, vacuolization/demyelination	CDV ((RT-)qPCR), *Salmonella enterica* subsp. *enterica* ser. Enteritidis (BE)
17410070106	2017-02-26	raccoon	female	adult	SAW	shot	not specified	no significant findings	none
17410070107	2017-02-26	raccoon	male	adult	SK	shot	not specified	no significant findings	none
17410070108	2017-02-26	red fox	female	adult	SAW	shot	not specified	eosinophilic meningitis, vacuolization/demyelination	none
17410070109	2017-02-25	red fox	male	adult	SAW	shot	not specified	no significant findings	CDV ((RT-)qPCR)
17410070111	2017-02-26	red fox	male	adult	SAW	shot	not specified	no significant findings	none
17410070112	2017-02-25	red fox	female	adult	SAW	shot	not specified	non-suppurative meningoencephalitis, gliosis, satellitosis, neuronophagia, neuronal necrosis, vacuolization/demyelination	CDV ((RT-)qPCR)
17410070113	2017-02-15	red fox	female	adult	SAW	shot	not specified	no significant findings	FoxCV ((RT-)qPCR), *Yersinia enterocolitica* (BE)
17410071426	2017-02-27	raccoon	female	adult	SAW	shot	not specified	no significant findings	*Streptococcus canis* (BE)
17410071429	2017-02-27	raccoon	male	adult	HZ	shot	not specified	non-suppurative encephalitis	none
17410071430	2017-02-25	raccoon	male	adult	HZ	shot	not specified	no significant findings	none
17410071431	2017-02-26	raccoon	female	adult	HZ	shot	not specified	no significant findings	none
17410071432	2017-02-26	red fox	male	adult	HZ	shot	not specified	no significant findings	CDV ((RT-)qPCR)
17410071433	2017-02-28	red fox	male	adult	ABI	found dead	not specified	non-suppurative meningoencephalitis, gliosis	CDV ((RT-)qPCR)

Continued on the following page

Lab-ID	Date of death or discovery	Species	Gender	Age	Administrative District	Cause of death	Behavior	Histopathological findings in the brain	Pathogens detected (method)
17410071434	2017-02-26	red fox	female	adult	MSH	found dead	not specified	non-suppurative meningoencephalitis	CDV ((RT-)qPCR)
17410071435	2017-02-25	red fox	male	adult	HZ	found dead	not specified	non-suppurative meningoencephalitis	CDV ((RT-)qPCR)
17410072604	2017-02-27	red fox	male	adult	BK	shot	abnormal	no significant findings	CDV ((RT-)qPCR)
17410072605	2017-03-01	red fox	male	adult	MSH	shot	abnormal	no significant findings	CDV ((RT-)qPCR)
17410072606	2017-03-01	red fox	male	adult	BLK	found dead	not specified	vacuolization/demyelination	CDV ((RT-)qPCR)
17410072607	2017-02-28	red fox	male	adult	BLK	shot	not specified	no significant findings	CDV ((RT-)qPCR)
17410072608	2017-02-28	raccoon	male	adult	JL	shot	not specified	no significant findings	CDV ((RT-)qPCR)
17410072618	2017-02-28	raccoon	male	adult	JL	shot	not specified	no significant findings	none
17410075256	2017-03-01	raccoon	female	adult	HZ	shot	abnormal	gliosis	CDV ((RT-)qPCR)
17410075257	2017-03-02	red fox	female	adult	BK	not specified	not specified	vacuolization/demyelination	none
17410079985	2017-03-02	red fox	female	adult	SK	found dead	not specified	non-suppurative meningoencephalitis, gliosis	CDV ((RT-)qPCR)
17410079986	2017-03-03	red fox	female	adult	BLK	found dead	not specified	mixed meningoencephalitis	CDV ((RT-)qPCR)
17410079991	2017-03-02	red fox	female	adult	MSH	shot	not specified	non-suppurative meningitis, gliosis	CDV ((RT-)qPCR)
17410080616	2017-03-06	red fox	male	adult	SAW	shot	not specified	non-suppurative meningoencephalitis, gliosis, satellitosis	CDV ((RT-)qPCR)
17410082242	2017-03-06	raccoon	male	adult	SK	shot	not specified	no significant findings	CPV-2 ((RT-)qPCR)
17410082243	2017-03-06	red fox	female	adult	HAL	found dead	not specified	no significant findings	none
17410082244	2017-03-06	red fox	male	adult	MSH	found dead	not specified	gliosis, vacuolization/demyelination	CDV ((RT-)qPCR)
17410083852	2017-03-04	raccoon	female	adult	HZ	shot	not specified	non-suppurative meningitis, gliosis, vacuolization/demyelination	CDV ((RT-)qPCR)
17410083853	2017-03-06	red fox	male	adult	BLK	found dead	not specified	no significant findings	none

Continued on the following page

Lab-ID	Date of death or discovery	Species	Gender	Age	Administrative District	Cause of death	Behavior	Histopathological findings in the brain	Pathogens detected (method)
17410083854	2017-03-06	red fox	female	adult	SAW	shot	abnormal	non-suppurative meningitis	CDV ((RT-)qPCR)
17410084950	2017-03-08	red fox	male	adult	SDL	shot	not specified	gliosis, vacuolization/demyelination	CDV ((RT-)qPCR), *L. monocytogenes* (BE)
17410085363	2017-03-08	red fox	female	adult	HAL	not specified	not specified	non-suppurative meningitis, vacuolization/demyelination	CDV ((RT-)qPCR)
17410085364	2017-03-07	red fox	female	adult	SK	shot	not specified	non-suppurative meningitis, vacuolization/demyelination	CDV ((RT-)qPCR)
17410085365	2017-03-07	red fox	female	adult	MSH	shot	abnormal	non-suppurative meningoencephalitis, vacuolization/demyelination	CDV ((RT-)qPCR)
17410085366	2017-03-07	red fox	female	adult	SLK	found dead	not specified	non-suppurative meningoencephalitis	CDV ((RT-)qPCR)
17410089057	2017-02-28	red fox	female	adult	SAW	not specified	not specified	no significant findings	none
17410089058	2017-03-09	red fox	male	adult	ABI	not specified	not specified	non-suppurative meningitis, vacuolization/demyelination	CDV ((RT-)qPCR)
17410089059	2017-03-08	raccoon dog	female	adult	SAW	not specified	not specified	no significant findings	CPV-2 ((RT-)qPCR)
17410090894	2017-03-10	red fox	female	adult	BLK	shot	not specified	no significant findings	CDV ((RT-)qPCR)
17410094170	2017-03-11	red fox	male	adult	MSH	shot	not specified	no significant findings	CDV ((RT-)qPCR)
17410095952	2017-03-12	raccoon	male	adult	HZ	shot	not specified	vacuolization/demyelination	none
17410095953	2017-03-13	raccoon	male	adult	ABI	shot	not specified	no significant findings	none
17410095954	2017-03-13	red fox	male	adult	SK	shot	abnormal	vacuolization/demyelination	CDV ((RT-)qPCR)
17410095955	2017-03-11	red fox	female	adult	HZ	shot	not specified	vacuolization/demyelination	CDV ((RT-)qPCR)
17410095956	2017-03-14	raccoon	female	adult	MSH	shot	abnormal	mixed meningoencephalitis, gliosis	CDV ((RT-)qPCR)
17410102147	2017-03-15	raccoon	female	adult	BLK	shot	not specified	vacuolization/demyelination	none
17410102148	2017-03-15	red fox	male	adult	MSH	shot	not specified	non-suppurative meningoencephalitis, gliosis	CDV ((RT-)qPCR)
17410102149	2017-03-15	red fox	male	adult	MSH	shot	not specified	no significant findings	CDV ((RT-)qPCR)

Continued on the following page

Lab-ID	Date of death or discovery	Species	Gender	Age	Administrative District	Cause of death	Behavior	Histopathological findings in the brain	Pathogens detected (method)
17410102154	2017-03-12	red fox	male	adult	SK	shot	not specified	non-suppurative meningitis, vacuolization/demyelination	CDV ((RT-)qPCR)
17410105664	2017-03-14	red fox	female	adult	BLK	shot	not specified	granulomatous encephalitis	larvae of nematodes (HE)
17410105665	2017-03-16	red fox	male	adult	BLK	shot	abnormal	non-suppurative meningitis	CDV/FoxCV ((RT-)qPCR)
17410105666	2017-03-16	badger	male	adult	BLK	found dead	not specified	non-suppurative meningitis	CDV/FoxCV ((RT-)qPCR)
17410105667	2017-03-16	raccoon dog	female	adult	SAW	shot	not specified	no significant findings	none
17410105773	2017-03-16	raccoon dog	male	adult	SAW	shot	not specified	no significant findings	none
17410107937	2017-03-20	raccoon	female	adult	SAW	shot	not specified	no significant findings	CDV ((RT-)qPCR)
17410107938	2017-03-18	red fox	female	adult	MSH	shot	not specified	no significant findings	CDV ((RT-)qPCR)
17410108000	2017-03-18	red fox	female	adult	MSH	shot	not specified	no significant findings	CDV ((RT-)qPCR)
17410108029	2017-03-19	red fox	male	adult	MSH	shot	not specified	no significant findings	CDV ((RT-)qPCR)
17410111161	2017-03-16	red fox	male	adult	SK	shot	not specified	non-suppurative meningitis	CDV ((RT-)qPCR)
17410111162	2017-03-16	red fox	male	adult	SK	found dead	not specified	non-suppurative meningoencephalitis, gliosis	CDV ((RT-)qPCR)
17410111163	2017-03-20	red fox	female	adult	HAL	shot	not specified	no significant findings	CDV ((RT-)qPCR)
17410111164	2017-03-14	raccoon	male	adult	SK	shot	not specified	no significant findings	CDV/CPV-2 ((RT-)qPCR)
17410113191	2017-03-14	badger	male	adult	HZ	found dead	not specified	no significant findings	CDV ((RT-)qPCR)
17410113192	2017-03-20	red fox	male	adult	BK	found dead	not specified	non-suppurative meningoencephalitis, gliosis	CDV ((RT-)qPCR)
17410113193	2017-03-22	badger	male	adult	MSH	found dead	not specified	non-suppurative meningoencephalitis, gliosis	CDV ((RT-)qPCR)
17410116739	2017-03-23	red fox	male	adult	MSH	found dead	abnormal	non-suppurative meningitis, gliosis, vacuolization/demyelination	CDV ((RT-)qPCR)
17410116740	2017-03-22	red fox	male	adult	MSH	found dead	not specified	no significant findings	CDV ((RT-)qPCR)
17410116752	2017-03-22	red fox	female	adult	SAW	shot	abnormal	no significant findings	CDV ((RT-)qPCR)
17410118023	2017-03-23	red fox	male	adult	HZ	shot	not specified	gliosis, satellitosis	none

Continued on the following page

Lab-ID	Date of death or discovery	Species	Gender	Age	Administrative District	Cause of death	Behavior	Histopathological findings in the brain	Pathogens detected (method)
17410119396	2017-03-25	red fox	male	adult	SAW	shot	abnormal	no significant findings	CDV ((RT-)qPCR), *Pasteurella canis* (BE)
17410119401	2017-03-26	red fox	female	adult	SLK	shot	not specified	no significant findings	CDV ((RT-)qPCR)
17410119402	2017-03-27	raccoon	male	adult	MD	shot	not specified	gliosis	none
17410122501	2017-03-24	red fox	male	adult	HZ	shot	not specified	no significant findings	none
17410122522	2017-03-27	red fox	female	adult	SAW	found dead	not specified	non-suppurative meningitis, vacuolization/demyelination	none
17410122553	2017-03-26	red fox	female	adult	SAW	shot	not specified	no significant findings	none
17410122555	2017-03-24	red fox	male	adult	SAW	shot	abnormal	non-suppurative meningitis, gliosis, vacuolization/demyelination	CDV ((RT-)qPCR)
17410125678	2017-03-28	red fox	male	adult	HZ	shot	not specified	mixed meningoencephalitis, gliosis	CDV ((RT-)qPCR)
17410128735	2017-03-28	red fox	male	adult	BK	found dead	not specified	no significant findings	CDV ((RT-)qPCR)
17410128737	2017-03-29	red fox	female	adult	BLK	shot	abnormal	no significant findings	none
17410128743	2017-03-29	red fox	female	adult	SLK	found dead	not specified	non-suppurative meningitis, gliosis, satellitosis, neuronophagia, vacuolization/demyelination	CDV ((RT-)qPCR)
17410128744	2017-03-29	red fox	male	adult	MSH	shot	not specified	no significant findings	CDV ((RT-)qPCR)
17410132910	2017-03-30	red fox	female	adult	HAL	found dead	not specified	no significant findings	none
17410132911	2017-03-30	red fox	female	adult	MSH	shot	abnormal	non-suppurative meningitis, gliosis	CDV ((RT-)qPCR)
17410132912	2017-03-30	red fox	female	adult	WB	found dead	not specified	no significant findings	CDV ((RT-)qPCR)
17410133009	2017-03-29	red fox	female	adult	HZ	shot	abnormal	non-suppurative meningitis, gliosis	CDV ((RT-)qPCR)
17410135365	2017-03-30	red fox	male	adult	MD	found dead	not specified	non-suppurative meningitis	CDV ((RT-)qPCR), *L. monocytogenes* (BE)
17410135366	2017-04-02	red fox	female	adult	SLK	found dead	not specified	no significant findings	CDV ((RT-)qPCR)
17410135367	2017-04-02	red fox	male	adult	HZ	shot	not specified	non-suppurative meningitis, vacuolization/demyelination	CDV ((RT-)qPCR)

Continued on the following page

Lab-ID	Date of death or discovery	Species	Gender	Age	Administrative District	Cause of death	Behavior	Histopathological findings in the brain	Pathogens detected (method)
17410135368	2017-04-03	red fox	female	adult	HZ	shot	not specified	non-suppurative meningitis	CDV ((RT-)qPCR)
17410137408	2017-04-04	red fox	male	adult	BK	found dead	not specified	no significant findings	none
17410137419	2017-04-02	red fox	male	adult	SK	shot	not specified	non-suppurative meningitis	none
17410137420	2017-04-04	red fox	male	adult	SAW	shot	not specified	no significant findings	CDV ((RT-)qPCR)
17410137421	2017-04-03	red fox	female	adult	MD	shot	not specified	non-suppurative encephalitis, vacuolization/demyelination, malacia	CDV ((RT-)qPCR)
17410137422	2017-04-01	red fox	male	adult	HZ	shot	not specified	no significant findings	CDV ((RT-)qPCR)
17410142941	2017-04-06	red fox	female	adult	HZ	shot	not specified	non-suppurative encephalitis, gliosis, satellitosis, neuronophagia, neuronal necrosis	CDV ((RT-)qPCR)
17410142942	2017-04-05	red fox	male	adult	WB	found dead	not specified	vacuolization/demyelination	CDV ((RT-)qPCR)
17410142943	2017-04-05	red fox	female	adult	MSH	shot	abnormal	gliosis, satellitosis, neuronophagia, vacuolization/demyelination	CDV ((RT-)qPCR)
17410145098	2017-04-06	red fox	male	adult	BK	found dead	not specified	no significant findings	CDV ((RT-)qPCR)
17410145099	2017-04-05	red fox	male	adult	HZ	shot	not specified	no significant findings	CDV ((RT-)qPCR)
17410147155	2017-04-07	red fox	female	adult	SLK	shot	abnormal	no significant findings	CDV ((RT-)qPCR)
17410147156	2017-04-09	red fox	female	adult	SK	shot	abnormal	no significant findings	CDV ((RT-)qPCR)
17410147157	2017-04-08	red fox	female	adult	SLK	shot	abnormal	no significant findings	CDV ((RT-)qPCR)
17410147159	2017-04-07	red fox	female	adult	HZ	shot	not specified	non-suppurative encephalitis, gliosis, neuronophagia, neuronal necrosis, vacuolization/demyelination	CDV ((RT-)qPCR)
17410148875	2017-04-10	red fox	female	adult	SAW	shot	abnormal	gliosis	CDV ((RT-)qPCR)
17410152291	2017-04-12	red fox	female	adult	SK	shot	not specified	gliosis, vacuolization/demyelination	CDV ((RT-)qPCR)
17410152292	2017-04-12	red fox	female	adult	HZ	shot	abnormal	no significant findings	CDV ((RT-)qPCR)
17410154699	2017-04-15	red fox	male	adult	HZ	found dead	abnormal	non-suppurative encephalitis	CDV ((RT-)qPCR)

Continued on the following page

Lab-ID	Date of death or discovery	Species	Gender	Age	Administrative District	Cause of death	Behavior	Histopathological findings in the brain	Pathogens detected (method)
17410154737	2017-04-17	red fox	male	adult	MSH	shot	not specified	no significant findings	none
17410154738	2017-04-13	red fox	female	adult	BLK	shot	abnormal	non-suppurative encephalitis	none
17410160429	2017-04-21	red fox	male	adult	SLK	shot	not specified	no significant findings	CDV/CPV-2 ((RT-)qPCR)
17410163708	2017-04-21	red fox	female	adult	BLK	shot	abnormal	vacuolization/demyelination	CDV ((RT-)qPCR)
17410163709	2017-04-22	red fox	male	adult	BLK	shot	not specified	non-suppurative meningitis, gliosis, vacuolization/demyelination	CDV ((RT-)qPCR)
17410163710	2017-04-24	red fox	male	adult	SK	shot	not specified	non-suppurative encephalitis, vacuolization/demyelination	CDV ((RT-)qPCR)
17410166019	2017-04-25	raccoon	female	adult	HZ	shot	not specified	non-suppurative meningitis, gliosis, vacuolization/demyelination	CDV ((RT-)qPCR)
17410166020	2017-04-24	red fox	female	adult	HZ	shot	not specified	non-suppurative meningoencephalitis, gliosis, satellitosis, vacuolization/demyelination	CDV ((RT-)qPCR)
17410166021	2017-04-24	red fox	male	adult	SLK	shot	abnormal	vacuolization/demyelination	CDV ((RT-)qPCR)
17410169464	2017-04-25	red fox	female	adult	SK	shot	not specified	non-suppurative meningitis	CDV ((RT-)qPCR)
17410169466	2017-04-25	red fox	male	adult	SLK	shot	abnormal	no significant findings	CDV/CPV-2 ((RT-)qPCR)
17410175312	2017-04-28	red fox	male	adult	MSH	shot	not specified	non-suppurative encephalitis	CDV ((RT-)qPCR)
17410177366	2017-05-01	red fox	male	adult	SK	shot	abnormal	no significant findings	none
17410177367	2017-04-29	red fox	male	adult	SK	shot	abnormal	non-suppurative encephalitis	FoxCV ((RT-)qPCR)
17410177368	2017-05-01	raccoon	male	adult	HZ	shot	not specified	gliosis	FoxCV ((RT-)qPCR)
17410178820	2017-04-30	raccoon	female	adult	HZ	shot	not specified	non-suppurative encephalitis, gliosis	none
17410183741	2017-05-03	raccoon	male	adult	SAW	found dead	not specified	no significant findings	none
17410183742	2017-05-04	red fox	female	juvenile	HZ	shot	not specified	non-suppurative meningoencephalitis, gliosis	none
17410183743	2017-05-04	raccoon	male	adult	HZ	shot	not specified	gliosis, vacuolization/demyelination	none
17410187800	2017-05-09	red fox	female	adult	BLK	found dead	not specified	granulomatous encephalitis, gliosis	CDV ((RT-)qPCR)

Continued on the following page

Lab-ID	Date of death or discovery	Species	Gender	Age	Administrative District	Cause of death	Behavior	Histopathological findings in the brain	Pathogens detected (method)
17410189710	2017-05-09	raccoon	female	adult	BLK	shot	abnormal	mixed meningoencephalitis, gliosis, satellitosis, neuronophagia	CDV/CPV-2 ((RT-)qPCR)
17410192310	2017-05-08	red fox	male	adult	JL	shot	not specified	no significant findings	none
17410192311	2017-05-11	red fox	female	adult	MD	shot	not specified	non-suppurative encephalitis	CDV ((RT-)qPCR)
17410192312	2017-05-10	red fox	female	adult	MSH	shot	not specified	non-suppurative meningitis	none
17410192313	2017-05-10	red fox	female	adult	SAW	shot	not specified	no significant findings	CDV ((RT-)qPCR)
17410192504	2017-05-10	raccoon	female	adult	HZ	shot	not specified	no significant findings	CDV ((RT-)qPCR)
17410194691	2017-05-10	red fox	male	adult	SAW	shot	not specified	non-suppurative meningitis	CDV ((RT-)qPCR)
17410194693	2017-05-10	raccoon	male	adult	MSH	shot	abnormal	granulomatous encephalitis, gliosis	CDV ((RT-)qPCR)
17410194694	2017-05-08	raccoon	male	adult	MSH	shot	abnormal	granulomatous encephalitis, gliosis, satellitosis	CDV ((RT-)qPCR)
17410195727	2017-05-13	red fox	female	adult	HZ	shot	abnormal	vacuolization/demyelination	none
17410195728	2017-05-15	red fox	female	adult	HZ	shot	not specified	no significant findings	CDV ((RT-)qPCR)
17410197695	2017-05-12	red fox	female	adult	SK	shot	abnormal	non-suppurative meningoencephalitis, gliosis	FoxCV ((RT-)qPCR)
17410201083	2017-05-15	red fox	male	adult	BK	shot	not specified	non-suppurative meningitis	CDV ((RT-)qPCR)
17410203606	2017-05-14	red fox	female	adult	BLK	shot	not specified	no significant findings	FoxCV ((RT-)qPCR)
17410203703	2017-05-18	red fox	male	adult	SAW	shot	not specified	non-suppurative meningoencephalitis	FoxCV ((RT-)qPCR)
17410203705	2017-05-18	raccoon	female	adult	MSH	shot	abnormal	no significant findings	none
17410203706	2017-05-18	raccoon	male	adult	MSH	shot	abnormal	no significant findings	none
17410206753	2017-05-21	raccoon	male	adult	SK	shot	not specified	vacuolization/demyelination	none
17410208329	2017-05-23	red fox	male	adult	SLK	shot	abnormal	non-suppurative meningitis, gliosis, vacuolization/demyelination	CDV/FoxCV ((RT-)qPCR)
17410208330	2017-05-24	red fox	male	adult	SK	shot	not specified	non-suppurative meningoencephalitis, gliosis, satellitosis	CDV ((RT-)qPCR)
17410208331	2017-05-23	raccoon	male	adult	BLK	shot	not specified	non-suppurative meningoencephalitis	none
17410208332	2017-05-24	raccoon	male	adult	HZ	shot	not specified	no significant findings	none
17410213040	2017-05-27	raccoon	male	adult	SK	shot	not specified	non-suppurative encephalitis	none

Continued on the following page

Lab-ID	Date of death or discovery	Species	Gender	Age	Administrative District	Cause of death	Behavior	Histopathological findings in the brain	Pathogens detected (method)
17410213041	2017-05-29	raccoon	female	adult	HZ	shot	not specified	no significant findings	none
17410213042	2017-05-30	raccoon	male	adult	HZ	shot	not specified	no significant findings	CDV ((RT-)qPCR)
17410213043	2017-05-28	red fox	female	adult	SAW	found dead	not specified	non-suppurative meningitis	CDV/CPV-2 ((RT-)qPCR)
17410217258	2017-05-30	raccoon	male	adult	SAW	shot	not specified	no significant findings	none
17410217259	2017-05-27	raccoon	female	adult	SAW	shot	not specified	no significant findings	none
17410217923	2017-05-31	red fox	male	adult	BLK	shot	not specified	non-suppurative meningitis	CPV-2/FoxCV ((RT-)qPCR)
17410218740	2017-06-01	marten	male	adult	BK	found dead	not specified	non-suppurative encephalitis	*Streptococcus canis* (BE)
17410218741	2017-06-01	raccoon	female	adult	BLK	shot	not specified	no significant findings	CPV-2 ((RT-)qPCR)
17410219958	2017-06-04	raccoon	male	juvenile	BLK	shot	not specified	no significant findings	none
17410219959	2017-06-03	red fox	male	adult	HZ	found dead	not specified	no significant findings	CDV ((RT-)qPCR)
17410219960	2017-06-02	red fox	male	adult	SAW	shot	not specified	no significant findings	none
17410225598	2017-06-11	marten	female	adult	BLK	shot	abnormal	non-suppurative encephalitis, gliosis	none
17410225599	2017-06-11	red fox	male	adult	WB	shot	abnormal	no significant findings	none
17410227453	2017-06-12	raccoon	male	adult	HZ	shot	not specified	no significant findings	none
17410229335	2017-06-13	raccoon	female	adult	MSH	shot	not specified	no significant findings	none
17410229336	2017-06-13	red fox	male	juvenile	MSH	shot	not specified	no significant findings	CPV-2 ((RT-)qPCR)
17410230861	2017-06-14	raccoon	male	adult	SAW	shot	not specified	no significant findings	CDV/FoxCV ((RT-)qPCR)
17410231849	2017-06-14	raccoon	female	adult	HZ	shot	not specified	no significant findings	none
17410233196	2017-06-18	raccoon	male	juvenile	SAW	shot	not specified	no significant findings	none
17410233197	2017-06-18	raccoon	female	juvenile	HZ	shot	not specified	vacuolization/demyelination	none
17410233198	2017-06-19	raccoon	female	juvenile	BLK	found dead	not specified	no significant findings	none
17410233202	2017-06-18	raccoon	female	juvenile	SAW	shot	not specified	no significant findings	none
17410234679	2017-06-19	raccoon	male	juvenile	SLK	shot	not specified	no significant findings	none
17410236053	2017-06-21	raccoon	female	adult	SLK	shot	not specified	no significant findings	CPV-2 ((RT-)qPCR)

Continued on the following page

Lab-ID	Date of death or discovery	Species	Gender	Age	Administrative District	Cause of death	Behavior	Histopathological findings in the brain	Pathogens detected (method)
17410236054	2017-06-20	red fox	male	adult	WB	found dead	not specified	non-suppurative meningoencephalitis	CDV/FoxCV ((RT-)qPCR)
17410239185	2017-06-22	raccoon	female	adult	HZ	shot	not specified	no significant findings	CDV ((RT-)qPCR)
17410246476	2017-06-27	red fox	male	juvenile	HZ	shot	not specified	non-suppurative meningoencephalitis	none
17410246477	2017-06-27	red fox	female	juvenile	HZ	shot	not specified	granulomatous encephalitis, gliosis	CDV ((RT-)qPCR), *T. gondii* (IHC)
17410247825	2017-07-01	raccoon	female	adult	WB	shot	not specified	no significant findings	none
17410253198	2017-07-06	red fox	female	adult	HZ	shot	not specified	non-suppurative encephalitis, gliosis	none
17410253745	2017-07-09	red fox	male	juvenile	WB	shot	not specified	no significant findings	none
17410253746	2017-07-08	red fox	male	adult	HZ	shot	abnormal	no significant findings	CDV ((RT-)qPCR)
17410253747	2017-07-08	raccoon	male	adult	HZ	shot	not specified	no significant findings	none
17410254906	2017-07-07	red fox	female	adult	SAW	shot	not specified	non-suppurative encephalitis	CDV ((RT-)qPCR)
17410254912	2017-07-11	raccoon	female	adult	WB	found dead	not specified	no significant findings	none
17410254940	2017-07-10	raccoon	female	adult	BLK	found dead	not specified	gliosis, satellitosis	CPV-2 ((RT-)qPCR)
17410258490	2017-07-13	raccoon	male	adult	JL	shot	not specified	no significant findings	none
17410258491	2017-07-13	raccoon	male	adult	JL	shot	not specified	no significant findings	none
17410258492	2017-07-13	raccoon	female	adult	JL	shot	not specified	no significant findings	none
17410259340	2017-07-17	raccoon	male	juvenile	HZ	shot	not specified	no significant findings	none
17410259342	2017-07-17	raccoon	female	juvenile	HZ	shot	not specified	no significant findings	none
17410259344	2017-07-17	raccoon	male	juvenile	HZ	shot	not specified	no significant findings	none
17410260558	2017-07-18	red fox	male	juvenile	SAW	shot	not specified	no significant findings	CDV ((RT-)qPCR)
17410261379	2017-07-18	raccoon	female	juvenile	HZ	found dead	abnormal	no significant findings	none
17410262531	2017-07-20	badger	male	adult	SAW	shot	abnormal	gliosis, vacuolization/demyelination	none
17410265076	2017-07-21	marten	female	adult	BLK	found dead	not specified	non-suppurative meningitis	none

Continued on the following page

Lab-ID	Date of death or discovery	Species	Gender	Age	Administrative District	Cause of death	Behavior	Histopathological findings in the brain	Pathogens detected (method)
17410265094	2017-07-20	raccoon	male	adult	HAL	not specified	not specified	no significant findings	none
17410266122	2017-07-23	red fox	female	adult	SAW	shot	not specified	no significant findings	CDV ((RT-)qPCR)
17410266124	2017-07-21	red fox	female	adult	HZ	not specified	not specified	non-suppurative meningoencephalitis, gliosis	CDV ((RT-)qPCR)
17410266125	2017-07-22	red fox	female	adult	HZ	shot	not specified	non-suppurative meningoencephalitis, gliosis, satellitosis	CDV ((RT-)qPCR)
17410266126	2017-07-23	red fox	male	adult	HZ	shot	not specified	non-suppurative meningoencephalitis, gliosis, satellitosis	CDV ((RT-)qPCR)
17410267256	2017-07-24	red fox	female	adult	SK	shot	not specified	gliosis, satellitosis, neuronophagia	none
17410267257	2017-07-24	raccoon	female	adult	HZ	shot	abnormal	no significant findings	CDV ((RT-)qPCR)
17410270950	2017-07-24	red fox	male	juvenile	SAW	shot	not specified	no significant findings	FoxCV ((RT-)qPCR)
17410271676	2017-07-27	raccoon	male	adult	BLK	shot	not specified	non-suppurative encephalitis	none
17410272721	2017-07-30	red fox	male	adult	SAW	shot	not specified	vacuolization/demyelination	none
17410272741	2017-07-30	red fox	female	juvenile	HZ	shot	not specified	non-suppurative meningoencephalitis	none
17410272743	2017-07-30	red fox	male	juvenile	HZ	shot	not specified	vacuolization/demyelination	none
17410272754	2017-07-30	red fox	female	adult	HZ	shot	not specified	vacuolization/demyelination	none
17410272755	2017-07-30	red fox	female	juvenile	HZ	shot	not specified	no significant findings	none
17410273917	2017-08-01	red fox	female	adult	SAW	shot	not specified	gliosis, satellitosis	none
17410276449	2017-08-02	red fox	male	adult	HZ	shot	not specified	non-suppurative encephalitis, gliosis, satellitosis	CDV ((RT-)qPCR)
17410276450	2017-08-02	raccoon dog	female	adult	HZ	shot	not specified	no significant findings	none
17410276451	2017-08-02	raccoon dog	female	adult	HZ	shot	not specified	no significant findings	none
17410277414	2017-08-03	raccoon	male	adult	HZ	shot	not specified	no significant findings	none
17410277415	2017-08-03	red fox	female	adult	HZ	shot	not specified	non-suppurative meningitis	CDV ((RT-)qPCR)
17410277423	2017-08-05	raccoon	female	adult	JL	shot	not specified	no significant findings	none
17410277424	2017-08-05	badger	female	adult	JL	shot	not specified	no significant findings	none
17410277425	2017-08-05	badger	male	adult	JL	shot	not specified	no significant findings	none

Continued on the following page

Lab-ID	Date of death or discovery	Species	Gender	Age	Administrative District	Cause of death	Behavior	Histopathological findings in the brain	Pathogens detected (method)
17410277857	2017-08-04	red fox	male	adult	SAW	shot	not specified	no significant findings	none
17410277858	2017-08-04	red fox	male	adult	HZ	shot	abnormal	non-suppurative encephalitis, gliosis, satellitosis	none
17410277859	2017-08-07	red fox	male	juvenile	BLK	shot	abnormal	gliosis	CDV ((RT-)qPCR)
17410277860	2017-08-07	raccoon	male	juvenile	BLK	shot	not specified	no significant findings	none
17410278848	2017-08-07	red fox	female	juvenile	SK	shot	not specified	mixed encephalitis	none
17410281519	2017-08-08	raccoon	male	adult	SAW	shot	not specified	no significant findings	none
17410281520	2017-08-09	red fox	female	adult	HZ	shot	not specified	no significant findings	none
17410283486	2017-08-13	raccoon dog	male	adult	SAW	shot	not specified	gliosis	none
17410283487	2017-08-13	raccoon	female	adult	HZ	shot	not specified	no significant findings	nonhemolytic *Escherichia coli* (BE)
17410283488	2017-08-13	red fox	male	adult	HZ	shot	not specified	no significant findings	CDV ((RT-)qPCR)
17410283489	2017-08-13	raccoon	male	juvenile	HZ	shot	not specified	no significant findings	none
17410283491	2017-08-13	raccoon	female	adult	HZ	shot	not specified	non-suppurative meningitis	none
17410284686	2017-08-15	raccoon	female	juvenile	SAW	found dead	not specified	no significant findings	none
17410284724	2017-08-12	raccoon	female	adult	SK	shot	not specified	no significant findings	none
17410284725	2017-08-12	red fox	male	adult	BLK	shot	not specified	no significant findings	none
17410284726	2017-08-12	badger	male	adult	SAW	shot	not specified	no significant findings	none
17410286357	2017-08-16	raccoon dog	male	adult	SAW	shot	not specified	non-suppurative meningitis	none
17410288130	2017-08-17	red fox	male	adult	BLK	shot	not specified	no significant findings	none
17410288131	2017-08-16	red fox	male	adult	HZ	shot	not specified	no significant findings	CDV ((RT-)qPCR)
17410289933	2017-08-16	red fox	male	adult	SLK	shot	not specified	no significant findings	FoxCV ((RT-)qPCR)
17410289934	2017-08-16	red fox	male	adult	BK	shot	not specified	gliosis, vacuolization/demyelination	none
17410291411	2017-08-22	red fox	male	adult	DE	shot	not specified	no significant findings	none
17410291412	2017-08-14	raccoon	male	adult	SAW	shot	not specified	no significant findings	none
17410291413	2017-08-19	raccoon	male	adult	HZ	shot	not specified	vacuolization/demyelination	none

Continued on the following page

Lab-ID	Date of death or discovery	Species	Gender	Age	Administrative District	Cause of death	Behavior	Histopathological findings in the brain	Pathogens detected (method)
17410291552	2017-08-23	red fox	female	adult	SDL	shot	not specified	gliosis	CPV-2/FoxCV ((RT-)qPCR)
17410294512	2017-08-24	red fox	female	juvenile	HZ	shot	not specified	vacuolization/demyelination	CDV ((RT-)qPCR)
17410294513	2017-08-23	raccoon	female	adult	BK	shot	not specified	no significant findings	CPV-2 ((RT-)qPCR)
17410295582	2017-08-24	raccoon	male	juvenile	HZ	shot	not specified	no significant findings	none
17410295583	2017-08-24	raccoon	male	juvenile	HZ	shot	not specified	no significant findings	none
17410295584	2017-08-24	raccoon	female	juvenile	HZ	shot	not specified	no significant findings	none
17410295585	2017-08-24	raccoon	male	adult	HZ	shot	not specified	gliosis, vacuolization/demyelination	CPV-2 ((RT-)qPCR)
17410298145	2017-08-28	red fox	male	adult	BK	shot	not specified	no significant findings	none
17410298146	2017-08-28	raccoon	male	juvenile	HZ	shot	not specified	mixed encephalitis	none
17410299362	2017-08-29	raccoon	male	juvenile	SAW	shot	not specified	no significant findings	none
17410302147	2017-09-01	raccoon dog	female	adult	SAW	shot	not specified	no significant findings	none
17410303046	2017-09-04	red fox	male	adult	HZ	shot	not specified	vacuolization/demyelination	none
17410304018	2017-09-02	red fox	female	adult	SAW	shot	not specified	no significant findings	CDV ((RT-)qPCR)
17410304020	2017-09-04	red fox	male	adult	BLK	shot	not specified	gliosis	none
17410304101	2017-09-05	red fox	male	adult	BK	shot	not specified	non-suppurative meningitis, gliosis, vacuolization/demyelination	none
17410304119	2017-09-05	red fox	male	adult	BK	shot	not specified	gliosis, satellitosis, vacuolization/demyelination	none
17410305375	2017-09-05	raccoon	female	adult	SAW	shot	not specified	no significant findings	none
17410305376	2017-09-05	red fox	male	adult	DE	shot	not specified	no significant findings	CDV/FoxCV ((RT-)qPCR)
17410305377	2017-09-04	raccoon	male	adult	HZ	shot	not specified	non-suppurative encephalitis	none
17410308284	2017-09-08	red fox	female	adult	BLK	shot	not specified	mixed meningoencephalitis, vacuolization/demyelination	CDV/FoxCV ((RT-)qPCR), *T. gondii* (IHC)
17410308285	2017-09-08	red fox	male	adult	SDL	shot	not specified	non-suppurative encephalitis, gliosis, satellitosis	none
17410309415	2017-09-10	red fox	male	adult	BK	shot	not specified	no significant findings	none
17410309416	2017-09-10	raccoon	male	adult	BK	shot	not specified	no significant findings	none

Continued on the following page

Lab-ID	Date of death or discovery	Species	Gender	Age	Administrative District	Cause of death	Behavior	Histopathological findings in the brain	Pathogens detected (method)
17410309417	2017-09-08	red fox	female	adult	BK	shot	not specified	no significant findings	none
17410312008	2017-09-13	red fox	female	adult	BLK	shot	not specified	no significant findings	none
17410316636	2017-09-18	red fox	male	adult	BK	shot	not specified	non-suppurative meningoencephalitis	none
17410316637	2017-09-19	red fox	male	adult	MSH	shot	not specified	no significant findings	none
17410316638	2017-09-18	red fox	male	adult	BK	shot	not specified	no significant findings	none
17410316652	2017-09-18	red fox	male	adult	BLK	shot	not specified	granulomatous encephalitis, vacuolization/demyelination	CDV ((RT-)qPCR), larvae of nematodes (HE)
17410316653	2017-09-16	red fox	male	adult	BLK	shot	not specified	suppurative meningoencephalitis	*Streptococcus canis* (BE)
17410318648	2017-09-19	raccoon dog	male	adult	BK	shot	not specified	non-suppurative meningitis, gliosis, satellitosis	none
17410321358	2017-09-21	red fox	male	adult	SK	shot	not specified	non-suppurative encephalitis, gliosis	FoxCV ((RT-)qPCR)
17410322778	2017-09-16	raccoon dog	female	adult	SAW	shot	not specified	no significant findings	none
17410322779	2017-09-16	raccoon dog	male	adult	SAW	shot	not specified	no significant findings	none
17410323684	2017-09-24	red fox	female	adult	BK	shot	not specified	non-suppurative meningoencephalitis, gliosis, satellitosis, neuronophagia	CDV ((RT-)qPCR)
17410324695	2017-09-24	red fox	female	adult	HAL	shot	abnormal	non-suppurative meningoencephalitis, gliosis	CDV ((RT-)qPCR)
17410328750	2017-09-27	red fox	female	adult	BK	shot	not specified	gliosis	none
17410328752	2017-09-27	raccoon dog	female	adult	BK	shot	not specified	no significant findings	none
17410332062	2017-10-01	red fox	male	adult	BK	shot	not specified	vacuolization/demyelination	none
17410332063	2017-10-01	red fox	male	adult	BK	shot	not specified	no significant findings	none
17410332064	2017-10-01	red fox	female	adult	HZ	found dead	not specified	no significant findings	none
17410332065	2017-10-01	raccoon	male	adult	BK	shot	not specified	no significant findings	CPV-2 ((RT-)qPCR)
17410332066	2017-10-01	raccoon	male	adult	BK	shot	not specified	no significant findings	none
17410334639	2017-10-04	red fox	female	adult	BLK	shot	not specified	non-suppurative meningitis	none
17410334640	2017-10-04	raccoon	female	adult	SK	shot	not specified	no significant findings	none
17410336258	2017-10-05	raccoon	female	adult	BK	shot	not specified	no significant findings	none

Continued on the following page

Lab-ID	Date of death or discovery	Species	Gender	Age	Administrative District	Cause of death	Behavior	Histopathological findings in the brain	Pathogens detected (method)
17410336259	2017-10-05	marten	male	adult	ABI	found dead	not specified	no significant findings	none
17410337257	2017-10-06	red fox	female	adult	BLK	shot	not specified	no significant findings	none
17410337258	2017-10-09	red fox	female	adult	HZ	shot	not specified	non-suppurative meningitis, gliosis, satellitosis, vacuolization/demyelination	none
17410337289	2017-10-06	raccoon	female	adult	MD	shot	not specified	no significant findings	none
17410339095	2017-10-09	raccoon dog	female	adult	SAW	shot	abnormal	gliosis	CDV ((RT-)qPCR)
17410339096	2017-10-09	red fox	female	adult	SAW	shot	not specified	non-suppurative encephalitis, vacuolization/demyelination	none
17410339198	2017-10-08	raccoon	male	adult	SAW	shot	not specified	no significant findings	none
17410339201	2017-10-10	red fox	male	adult	BLK	shot	not specified	no significant findings	none
17410339204	2017-10-10	raccoon	male	adult	SK	shot	not specified	gliosis	none
17410339210	2017-10-09	red fox	female	adult	SAW	shot	not specified	vacuolization/demyelination	none
17410340294	2017-10-10	red fox	male	adult	SAW	shot	not specified	no significant findings	none
17410342915	2017-10-11	raccoon	male	juvenile	SK	shot	not specified	gliosis, vacuolization/demyelination	none
17410342937	2017-10-09	marten	female	adult	BLK	shot	not specified	no significant findings	none
17410344497	2017-10-12	red fox	male	adult	HZ	found dead	not specified	non-suppurative meningitis, vacuolization/demyelination	none
17410345874	2017-10-16	red fox	male	adult	SLK	shot	abnormal	gliosis, vacuolization/demyelination	FoxCV ((RT-)qPCR)
17410347212	2017-10-16	red fox	male	adult	BLK	shot	not specified	no significant findings	CDV ((RT-)qPCR)
17410347213	2017-10-14	raccoon dog	female	adult	ABI	shot	not specified	non-suppurative meningitis, gliosis, vacuolization/demyelination	FoxCV ((RT-)qPCR)
17410348605	2017-10-17	red fox	male	juvenile	WB	found dead	not specified	no significant findings	CDV ((RT-)qPCR)
17410348606	2017-10-17	red fox	male	adult	HZ	shot	not specified	no significant findings	CDV ((RT-)qPCR)
17410350913	2017-10-18	raccoon	male	adult	BLK	shot	not specified	granulomatous encephalitis	larvae of nematodes (HE)
17410351107	2017-10-19	raccoon	male	adult	BLK	found dead	not specified	non-suppurative meningitis, vacuolization/demyelination	none

Continued on the following page

Lab-ID	Date of death or discovery	Species	Gender	Age	Administrative District	Cause of death	Behavior	Histopathological findings in the brain	Pathogens detected (method)
17410353154	2017-10-20	red fox	female	adult	ABI	found dead	not specified	gliosis	none
17410354107	2017-10-21	red fox	male	adult	BK	shot	not specified	non-suppurative meningoencephalitis, gliosis, satellitosis	none
17410354112	2017-10-21	red fox	male	adult	BK	shot	not specified	non-suppurative meningoencephalitis	none
17410354113	2017-10-21	red fox	male	adult	BK	shot	not specified	non-suppurative meningoencephalitis	none
17410354114	2017-10-21	raccoon	male	juvenile	HZ	shot	not specified	no significant findings	none
17410354115	2017-10-23	raccoon	male	adult	HZ	shot	not specified	no significant findings	CPV-2 ((RT-)qPCR)
17410354116	2017-10-21	red fox	male	adult	ABI	shot	not specified	non-suppurative meningitis	none
17410354117	2017-10-20	red fox	male	adult	BLK	shot	not specified	no significant findings	none
17410357220	2017-10-19	red fox	male	adult	SK	found dead	not specified	no significant findings	none
17410364433	2017-10-25	raccoon	female	adult	ABI	shot	not specified	no significant findings	CPV-2 ((RT-)qPCR)
17410367037	2017-10-30	raccoon	male	adult	ABI	shot	not specified	no significant findings	CPV-2 ((RT-)qPCR)
17410367041	2017-10-30	raccoon	male	adult	ABI	shot	not specified	no significant findings	none
17410367043	2017-10-28	red fox	male	adult	HZ	shot	not specified	no significant findings	none
17410367044	2017-10-28	red fox	male	adult	BK	shot	not specified	vacuolization/demyelination	FoxCV ((RT-)qPCR)
17410367055	2017-10-30	raccoon	female	adult	ABI	shot	not specified	no significant findings	none
17410370013	2017-11-01	red fox	male	adult	BK	shot	not specified	no significant findings	none
17410370014	2017-11-01	raccoon	male	adult	BK	shot	not specified	no significant findings	none
17410370857	2017-11-04	red fox	male	adult	SDL	shot	not specified	no significant findings	CPV-2 ((RT-)qPCR)
17410370861	2017-11-04	red fox	female	adult	SDL	shot	not specified	non-suppurative meningoencephalitis	none
17410370862	2017-11-04	red fox	male	adult	SDL	shot	not specified	no significant findings	CPV-2 ((RT-)qPCR)
17410370868	2017-11-04	red fox	male	adult	SDL	shot	not specified	no significant findings	none
17410373587	2017-11-06	red fox	female	adult	JL	shot	not specified	non-suppurative meningoencephalitis, gliosis	CDV ((RT-)qPCR)
17410373614	2017-11-06	red fox	female	adult	HZ	shot	not specified	non-suppurative meningitis, vacuolization/demyelination	none

Continued on the following page

Lab-ID	Date of death or discovery	Species	Gender	Age	Administrative District	Cause of death	Behavior	Histopathological findings in the brain	Pathogens detected (method)
17410373617	2017-11-05	red fox	female	adult	BK	shot	not specified	non-suppurative meningitis, vacuolization/demyelination	none
17410373618	2017-11-05	red fox	male	adult	BK	shot	not specified	non-suppurative meningitis	none
17410373620	2017-11-04	red fox	male	adult	BLK	shot	not specified	non-suppurative meningoencephalitis	none
17410373623	2017-11-04	red fox	female	adult	BLK	shot	not specified	no significant findings	none
17410373631	2017-11-04	red fox	male	adult	BLK	shot	not specified	non-suppurative meningitis	none
17410373640	2017-11-04	red fox	male	adult	BLK	shot	not specified	no significant findings	none
17410373642	2017-11-05	raccoon	male	adult	BK	shot	not specified	no significant findings	none
17410373644	2017-11-05	raccoon	male	adult	BK	shot	not specified	non-suppurative encephalitis, gliosis	none
17410375293	2017-11-04	red fox	male	adult	HZ	shot	not specified	no significant findings	none
17410375313	2017-11-04	red fox	male	adult	HZ	shot	not specified	gliosis	none
17410375314	2017-11-04	red fox	male	adult	HZ	shot	not specified	no significant findings	none
17410375317	2017-11-07	raccoon	female	adult	HZ	shot	not specified	non-suppurative meningitis	CPV-2 ((RT-)qPCR)
17410375318	2017-11-04	raccoon	male	adult	SAW	shot	not specified	no significant findings	CPV-2 ((RT-)qPCR)
17410376592	2017-11-03	red fox	male	adult	HZ	shot	not specified	no significant findings	none
17410376601	2017-11-03	raccoon	male	adult	HZ	shot	not specified	vacuolization/demyelination	none
17410379112	2017-11-08	red fox	male	adult	SAW	shot	not specified	no significant findings	FoxCV ((RT-)qPCR)
17410379281	2017-11-09	red fox	male	adult	BLK	found dead	not specified	mixed meningoencephalitis	CDV/FoxCV ((RT-)qPCR), *T. gondii* (IHC)
17410379282	2017-11-06	raccoon	female	adult	BK	shot	not specified	non-suppurative meningoencephalitis, gliosis	CDV/FoxCV ((RT-)qPCR), *L. monocytogenes* (BE)
17410381221	2017-11-09	red fox	male	adult	MSH	shot	not specified	no significant findings	none
17410381322	2017-11-09	red fox	female	adult	MSH	shot	not specified	no significant findings	none
17410384213	2017-11-11	raccoon	female	adult	BK	shot	not specified	gliosis	none
17410384241	2017-11-11	raccoon	male	adult	BK	shot	not specified	no significant findings	none
17410384242	2017-11-13	red fox	female	adult	BK	shot	not specified	no significant findings	none
17410384247	2017-11-13	red fox	male	adult	BK	shot	not specified	no significant findings	CPV-2 ((RT-)qPCR)

Continued on the following page

Lab-ID	Date of death or discovery	Species	Gender	Age	Administrative District	Cause of death	Behavior	Histopathological findings in the brain	Pathogens detected (method)
17410384248	2017-11-13	red fox	male	adult	BK	shot	not specified	no significant findings	none
17410384249	2017-11-13	red fox	female	adult	HZ	shot	not specified	no significant findings	none
17410386189	2017-11-11	red fox	male	adult	HZ	shot	abnormal	no significant findings	none
17410386191	2017-11-12	red fox	female	adult	SAW	shot	not specified	no significant findings	none
17410386195	2017-11-12	red fox	male	adult	SAW	shot	not specified	no significant findings	none
17410386196	2017-11-12	red fox	female	adult	SAW	shot	not specified	no significant findings	none
17410386302	2017-11-11	red fox	male	adult	BK	shot	not specified	non-suppurative meningitis	none
17410386303	2017-11-11	red fox	female	adult	BK	shot	not specified	vacuolization/demyelination	none
17410386329	2017-11-12	red fox	male	adult	BK	shot	not specified	gliosis	none
17410386330	2017-11-12	red fox	female	adult	BK	shot	not specified	non-suppurative encephalitis	none
17410386402	2017-11-12	badger	female	adult	BK	shot	not specified	non-suppurative meningoencephalitis	none
17410387895	2017-11-14	red fox	male	adult	BK	shot	not specified	non-suppurative encephalitis, gliosis	CPV-2/FoxCV ((RT-)qPCR)
17410387896	2017-11-11	red fox	male	adult	BK	shot	not specified	no significant findings	CPV-2/FoxCV ((RT-)qPCR)
17410387897	2017-11-10	red fox	male	adult	HZ	shot	not specified	non-suppurative meningitis	CPV-2/FoxCV ((RT-)qPCR)
17410387898	2017-11-12	red fox	male	adult	BK	shot	not specified	gliosis	CPV-2/FoxCV ((RT-)qPCR)
17410388152	2017-11-11	red fox	female	adult	HZ	shot	not specified	non-suppurative meningitis, vacuolization/demyelination	CDV ((RT-)qPCR)
17410388153	2017-11-11	red fox	female	adult	HZ	shot	not specified	non-suppurative meningitis	CPV-2/FoxCV ((RT-)qPCR)
17410394534	2017-11-16	red fox	male	adult	SAW	shot	not specified	no significant findings	none
17410397834	2017-11-20	red fox	male	adult	SAW	shot	not specified	no significant findings	CPV-2 ((RT-)qPCR)
17410397835	2017-11-18	red fox	female	adult	SAW	shot	not specified	no significant findings	none
17410397836	2017-11-20	red fox	female	adult	SAW	shot	not specified	no significant findings	none
17410397837	2017-11-20	red fox	male	adult	SAW	shot	not specified	no significant findings	none
17410397838	2017-11-19	raccoon dog	male	adult	SAW	shot	abnormal	non-suppurative encephalitis	none
17410400467	2017-11-18	red fox	male	adult	HZ	shot	not specified	vacuolization/demyelination	CDV ((RT-)qPCR)
17410400469	2017-11-18	red fox	male	adult	HZ	shot	not specified	no significant findings	none

Continued on the following page

Lab-ID	Date of death or discovery	Species	Gender	Age	Administrative District	Cause of death	Behavior	Histopathological findings in the brain	Pathogens detected (method)
17410400472	2017-11-18	red fox	female	adult	HZ	shot	not specified	no significant findings	CPV-2 ((RT-)qPCR)
17410400474	2017-11-18	red fox	female	adult	HZ	shot	not specified	no significant findings	CDV ((RT-)qPCR)
17410400476	2017-11-18	red fox	female	adult	HZ	shot	not specified	non-suppurative meningoencephalitis, gliosis, vacuolization/demyelination	CDV/CPV-2 ((RT-)qPCR)
17410400478	2017-11-18	red fox	male	adult	MSH	shot	not specified	granulomatous encephalitis, vacuolization/demyelination	CDV/CPV-2 ((RT-)qPCR), larvae of nematodes (HE)
17410400479	2017-11-19	red fox	female	adult	SLK	shot	abnormal	non-suppurative meningoencephalitis, gliosis, satellitosis, neuronophagia	CDV ((RT-)qPCR)
17410400480	2017-11-18	raccoon	female	adult	ABI	shot	not specified	non-suppurative meningitis, gliosis	none
17410402720	2017-11-18	red fox	female	adult	HZ	shot	not specified	no significant findings	CPV-2 ((RT-)qPCR)
17410402955	2017-11-17	red fox	male	adult	HZ	shot	not specified	non-suppurative meningoencephalitis, vacuolization/demyelination	CPV-2 ((RT-)qPCR)
17410402989	2017-11-17	red fox	male	adult	HZ	shot	not specified	no significant findings	CPV-2 ((RT-)qPCR)
17410403131	2017-11-17	red fox	male	adult	HZ	shot	not specified	no significant findings	CPV-2 ((RT-)qPCR)
17410403132	2017-11-21	raccoon	female	adult	ABI	shot	not specified	no significant findings	CPV-2 ((RT-)qPCR)
17410408543	2017-11-22	red fox	male	adult	HZ	shot	not specified	non-suppurative meningoencephalitis, gliosis	CDV ((RT-)qPCR), *L. monocytogenes* (BE)
17410411766	2017-11-23	badger	male	adult	DE	shot	not specified	no significant findings	none
17410413366	2017-11-25	red fox	male	adult	SAW	shot	not specified	non-suppurative meningoencephalitis	none
17410413367	2017-11-27	red fox	male	adult	HZ	shot	not specified	no significant findings	CDV ((RT-)qPCR)
17410413369	2017-11-26	raccoon	male	adult	BK	shot	not specified	no significant findings	CDV ((RT-)qPCR)
17410415651	2017-11-28	red fox	female	adult	HZ	shot	not specified	no significant findings	none
17410415652	2017-11-28	red fox	male	adult	HZ	shot	not specified	no significant findings	none
17410418717	2017-11-28	red fox	male	adult	BLK	found dead	not specified	no significant findings	CDV/FoxCV ((RT-)qPCR)
17410418718	2017-11-28	red fox	male	adult	BLK	found dead	not specified	vacuolization/demyelination	CDV/FoxCV ((RT-)qPCR)
17410418719	2017-11-27	raccoon	female	adult	HZ	shot	abnormal	non-suppurative meningoencephalitis, gliosis	CDV/FoxCV ((RT-)qPCR)

Continued on the following page

Lab-ID	Date of death or discovery	Species	Gender	Age	Administrative District	Cause of death	Behavior	Histopathological findings in the brain	Pathogens detected (method)
17410425222	2017-11-30	red fox	female	adult	MD	shot	abnormal	non-suppurative encephalitis	FoxCV ((RT-)qPCR)
17410426866	2017-12-01	red fox	female	adult	SAW	shot	not specified	no significant findings	none
17410427841	2017-12-03	red fox	male	adult	BLK	found dead	not specified	non-suppurative meningitis, gliosis, vacuolization/demyelination	CDV ((RT-)qPCR)
17410429916	2017-12-05	raccoon	male	adult	SAW	shot	not specified	no significant findings	CPV-2 ((RT-)qPCR)
17410430253	2017-12-02	red fox	female	adult	SAW	shot	not specified	no significant findings	none
17410430272	2017-12-04	red fox	male	adult	DE	shot	abnormal	no significant findings	CDV ((RT-)qPCR)
17410430275	2017-12-02	red fox	female	adult	SAW	shot	not specified	no significant findings	none
17410430277	2017-12-01	red fox	male	adult	HZ	shot	not specified	no significant findings	CDV ((RT-)qPCR)
17410430417	2017-12-01	red fox	female	adult	HZ	shot	not specified	no significant findings	none
17410430422	2017-12-02	raccoon	female	adult	HZ	shot	not specified	no significant findings	CDV ((RT-)qPCR)
17410430425	2017-12-02	red fox	male	adult	SDL	shot	abnormal	non-suppurative meningitis, vacuolization/demyelination	CDV/CPV-2 ((RT-)qPCR)
17410432745	2017-12-02	red fox	female	adult	HZ	shot	not specified	non-suppurative meningoencephalitis, gliosis, satellitosis	CDV ((RT-)qPCR)
17410432746	2017-12-02	red fox	female	adult	HZ	shot	not specified	no significant findings	none
17410432747	2017-12-03	red fox	male	adult	HZ	shot	not specified	gliosis	none
17410432748	2017-12-02	red fox	male	adult	HZ	shot	not specified	no significant findings	none
17410440098	2017-12-06	red fox	male	adult	SAW	shot	not specified	no significant findings	CDV ((RT-)qPCR)
17410440099	2017-12-05	red fox	male	adult	SAW	shot	not specified	no significant findings	CDV ((RT-)qPCR)
17410440100	2017-12-07	red fox	male	adult	SAW	shot	not specified	no significant findings	CDV ((RT-)qPCR)
17410441800	2017-12-10	red fox	male	adult	BLK	shot	not specified	non-suppurative meningitis	CPV-2 ((RT-)qPCR)
17410441803	2017-12-10	red fox	female	adult	BLK	shot	not specified	non-suppurative meningitis	none
17410444086	2017-12-12	red fox	male	adult	BK	shot	not specified	granulomatous encephalitis	larvae of nematodes (HE)
17410450272	2017-12-14	red fox	male	adult	SAW	shot	not specified	non-suppurative meningoencephalitis, gliosis, satellitosis, neuronophagia	none
17410453650	2017-12-12	red fox	female	adult	SAW	shot	not specified	no significant findings	FoxCV ((RT-)qPCR)

Continued on the following page

Lab-ID	Date of death or discovery	Species	Gender	Age	Administrative District	Cause of death	Behavior	Histopathological findings in the brain	Pathogens detected (method)
17410453652	2017-12-15	red fox	male	adult	BK	shot	not specified	non-suppurative meningitis	none
17410456316	2017-12-18	raccoon	male	adult	BLK	shot	not specified	no significant findings	none
17410456325	2017-12-18	raccoon	female	adult	BLK	shot	not specified	no significant findings	CPV-2 ((RT-)qPCR)
17410456326	2017-12-16	red fox	male	adult	SAW	shot	not specified	non-suppurative meningoencephalitis, gliosis	CDV ((RT-)qPCR)
17410458025	2017-12-20	red fox	male	adult	SAW	shot	not specified	non-suppurative meningitis	CPV-2 ((RT-)qPCR)
17410458026	2017-12-20	red fox	female	adult	SAW	shot	not specified	non-suppurative meningoencephalitis, gliosis	CDV ((RT-)qPCR)
17410461648	2017-12-22	red fox	female	adult	SAW	shot	not specified	no significant findings	none
17410461822	2017-12-27	raccoon dog	male	adult	SDL	shot	abnormal	non-suppurative meningoencephalitis, gliosis	CDV ((RT-)qPCR)
17410462803	2017-12-26	raccoon	female	adult	BK	shot	not specified	non-suppurative meningitis	none
17410462804	2017-12-27	red fox	male	adult	SK	shot	not specified	no significant findings	none

Table S2. Histopathological degree of meningitis and encephalitis. Abbreviation: High Power Field (HPF) = one visual field at 400x magnification

Degree	Localization	
	perivascular (brain and meninges)	periventricular/parenchymatous (brain)
abscent	no free inflammation cells/HPF	
minimal	occasionally up to 5 perivascularly located inflammatory cells/HPF	occasionally up to 5 inflammatory cells/HPF; the inflammation cells can occur singularly or as multifocal minimal inflammation cell foci
mild	6 - 30 perivascularly located inflammatory cells/HPF; single to double-layer	6 - 30 inflammatory cells/HPF; the inflammation cells can occur singularly and/or as multifocal mild inflammation cell foci or mild diffusely distributed; additional focal moderate focus possible
moderate	31 - 80 perivascularly located inflammatory cells/HPF; two- to three-layer	31 - 80 inflammatory cells/HPF; the inflammation cells can occur as multifocal moderate inflammation cell foci or moderate diffusely distributed; additionally focally a mild or a marked focus possible
marked	> 80 perivascularly located inflammatory cells/HPF; three- to multi-layer	> 80 inflammatory cells/HPF; the inflammation cells can appear as multifocal marked inflammation cell foci or marked diffusely distributed

Table S3. Temperature profiles and references of (RT-)qPCR for detection of suid herpesvirus 1 (SuHV-1), West nile virus (WNV), Borna disease virus 1 (BoDV-1), canine distemper virus (CDV), canid alphaherpesvirus 1 (CaHV-1), canine parvovirus type 2, 2a, 2b or 2c (CPV-2), fox circovirus (FoxCV) and *Neospora caninum* (*N. caninum*)

Pathogen	Function	Temperature (° C)	Time (min:sec)	Cycles	Reference
SuHV-1	Activation Polymerase	95	15:00		Wernike et al. (5)
	Denaturation	95	00:15	40	
	Annealing	54	00:22		
	Elongation	72	00:30		
WNV	Reverse Transcription	50	10:00		Eiden et al. (6)
	Inactivation Reverse Transcriptase/Activation Polymerase	95	05:00		
	Denaturation	95	00:10	42	
	Annealing	55	00:30		
	Elongation	72	00:30		
BoDV-1	Reverse Transcription	50	10:00		Schlottau et al. (7)
	Inactivation Reverse Transcriptase/Activation Polymerase	95	01:00		
	Denaturation	95	00:10	45	
	Annealing	57	00:30		
	Elongation	68	00:30		
CDV	Reverse Transcription	50	20:00		Elia et al. (8)
	Inactivation Reverse Transcriptase/Activation Polymerase	95	05:00		
	Denaturation	95	00:15	40	
	Annealing and Elongation	56	00:40		
CaHV-1	Activation Polymerase	95	15:00		Decaro et al. (9)
	Denaturation	95	00:45	45	
	Annealing und Elongation	60	01:00		
CPV-2	Activation Polymerase	95	15:00		Streck et al. (10)
	Denaturation	95	00:30	40	
	Annealing	58	00:30		
	Elongation	72	00:30		
FoxCV	Activation Polymerase	95	15:00		Bexton et al. (11)
	Denaturation	94	01:00	40	
	Annealing and Elongation	60	01:00		
N. caninum	Activation Polymerase	95	15:00		Constantin et al. (12)
	Denaturation	95	00:15	40	
	Annealing	54	00:20		
	Elongation	72	00:30		

REFERENCES

1 .Beineke A, Baumgärtner W, Wohlsein P. Cross-species transmission of canine distemper virus-an update. *One Heal.* **1** (2015) 49–59. doi:10.1016/j.onehlt.2015.09.002.

2 .Origgi FC, Plattet P, Sattler U, Robert N, Casaubon J, Mavrot F, et al. Emergence of canine distemper virus strains with modified molecular signature and enhanced neuronal tropism leading to high mortality in wild carnivores. *Vet. Pathol.* **49** (2012) 913–929. doi:10.1177/0300985812436743.

3 .van Moll P, Alldinger S, Baumgärtner W, Adami M. Distemper in wild carnivores: an epidemiological, histological and immunocytochemical study. *Vet. Microbiol.* **44** (1995) 193–199. doi:10.1016/0378-1135(95)00012-Y.

4 .Loots AK, Mitchell E, Dalton DL, Kotzé A, Venter EH. Advances in canine distemper virus pathogenesis research: a wildlife perspective. *J. Gen. Virol.* **98** (2017) 311–321. doi:10.1099/jgv.0.000666.

5 .Wernike K, Beer M, Freuling CM, Klupp B, Mettenleiter TC, Müller T, et al. Molecular double-check strategy for the identification and characterization of Suid herpesvirus 1. *J. Virol. Methods* **209** (2014) 110–115. doi:10.1016/j.jviromet.2014.08.022.

6 .Eiden M, Vina-Rodriguez A, Hoffmann B, Ziegler U, Groschup MH. Two new real-time quantitative reverse transcription polymerase chain reaction assays with unique target sites for the specific and sensitive detection of lineages 1 and 2 West Nile virus strains. *J. Vet. Diagnostic Investig.* **22** (2010) 748–753.

7 .Schlottau K, Forth L, Angstwurm K, Höper D, Zecher D, Liesche F, et al. Fatal encephalitic Borna disease virus 1 in solid-organ transplant recipients. *N. Engl. J. Med.* **379** (2018) 1377–1379. doi:10.1056/NEJMc1803115.

8 .Elia G, Decaro N, Martella V, Cirone F, Lucente MS, Lorusso E, et al. Detection of canine distemper virus in dogs by real-time RT-PCR. *J. Virol. Methods* **136** (2006) 171–176. doi:10.1016/j.jviromet.2006.05.004.

9 .Decaro N, Amorisco F, Desario C, Lorusso E, Camero M, Bellacicco AL, et al. Development and validation of a real-time PCR assay for specific and sensitive detection of canid herpesvirus 1. *J. Virol. Methods* **169** (2010) 176–180. doi:10.1016/j.jviromet.2010.07.021.

10 .Streck AF, Rüster D, Truyen U, Homeier T. An updated TaqMan real-time PCR for canine and feline parvoviruses. *J. Virol. Methods* **193** (2013) 6–8. doi:10.1016/j.jviromet.2013.04.025.

11 .Bexton S, Wiersma LC, Getu S, van Run PR, Verjans GM, Schipper D, et al. Detection of circovirus in foxes with meningoencephalitis, United Kingdom, 2009-2013. *Emerg. Infect. Dis.* **21** (2015) 1205–1208. doi:10.3201/eid2107.150228.

12 .Constantin EM, Schares G, Grossmann E, Sauter K, Romig T, Hartmann S. Studies on the role of the red fox (Vulpes vulpes) as a potential definitive host of Neospora caninum. *Berl. Munch. Tierarztl. Wochenschr.* **124** (2011) 148–153. doi:10.2376/0005-9366-124-148. [German].
